# Dual Antitubercular and Antileishmanial Profiles of Quinoxaline Di-*N*-Oxides Containing an Amino Acidic Side Chain

**DOI:** 10.3390/ph17040487

**Published:** 2024-04-11

**Authors:** Juan F. González, María-Auxiliadora Dea-Ayuela, Lena Huck, José María Orduña, Francisco Bolás-Fernández, Elena de la Cuesta, Nazia Haseen, Ashraf Ali Mohammed, J. Carlos Menéndez

**Affiliations:** 1 Unidad de Química Orgánica y Farmacéutica, Departamento de Química en Ciencias Farmacéuticas, Facultad de Farmacia, Universidad Complutense, Plaza de Ramón y Cajal s/n, 28040 Madrid, Spain; jfgonzal@ucm.es (J.F.G.); lenahuck@gmail.com (L.H.); jmordunamendez@gmail.com (J.M.O.); ecuestae@ucm.es (E.d.l.C.); 2 Departamento de Farmacia, Facultad de Ciencias de la Salud, Universidad Cardenal Herrera-CEU, CEU Universities, c/Santiago Ramón y Cajal, Alfara del Patriarca, 46115 Valencia, Spain; mdea@uchceu.es; 3 Departamento de Microbiología y Parasitología, Facultad de Farmacia, Universidad Complutense de Madrid, Plaza de Ramón y Cajal s/n, 28040 Madrid, Spain; francisb@ucm.es; 4 AMIPRO SDN.BHD. Level 3, Bangunan Inkubator Universiti, Sains@USM, Lebuh Bukit Jambul, Bayan Lepas 11900, Pulau Pinang, Malaysia; naziahaseen03@gmail.com (N.H.); ashraf@amiprolab.com (A.A.M.)

**Keywords:** *N*-oxides, antitubercular, antileishmanial, neglected tropical diseases, co-infections, drug design, structure–activity relationships

## Abstract

We present a new category of quinoxaline di-N-oxides (QdNOs) containing amino acid side chains with dual antituberculosis and antileishmanial activity. These compounds were synthesized by combining a regioselective 2,5-piperazinedione opening and a Beirut reaction and were screened for their activity against *Mycobacterium tuberculosis* and the promastigote and amastigote forms of representative species of the *Leishmania* genus. Most QdNOs exhibited promising antitubercular activity with IC_50_ values ranging from 4.28 to 49.95 μM, comparable to clinically established drugs. Structure–activity relationship analysis emphasized the importance of substituents on the aromatic ring and the side chain. Antileishmanial tests showed that some selected compounds exhibited activity comparable to the positive control miltefosine against promastigotes of *Leishmania amazonensis* and *Leishmania donovani*. Notably, some compounds were found to be also more potent and less toxic than miltefosine in intracellular amastigote assays against *Leishmania amazonensis*. The compound showing the best dual antitubercular and leishmanicidal profile and a good selectivity index, **4h**, can be regarded as a hit compound that opens up new opportunities for the development of integrated therapies against co-infections.

## 1. Introduction

Worldwide mortality and disability caused by infectious agents is a serious global health problem and one of the most important scientific challenges of our time [1]. Neglected tropical diseases (NTDs) are a group of protozoan, viral, bacterial or parasitic diseases that are particularly prevalent among the less favored population, mostly in tropical areas. Changes in migration patterns and environmental factors [2] may have complex effects on the distribution of disease vectors of NTDs such as malaria, Chagas disease and leishmaniasis, which may alter their geographical distribution. In particular, leishmaniasis (LH) and tuberculosis (TB) are endemic NTDs that affect a considerable fraction of the population in tropical and subtropical regions.

Worldwide, an estimated 1.6 million new cases of leishmaniasis occur annually, with approximately 12 million patients being currently infected in about 90 countries and about 350 million people being at risk. These cases include per year 700,000 to 1.2 million new cases of cutaneous leishmaniasis, a disfiguring disease, and 200,000 to 400,000 patients with visceral leishmaniasis (kala-azar), which proves fatal when left untreated [3,4].

Currently, there are no approved vaccines for human leishmaniasis [5], and the primary approach to combating the disease is focused on chemotherapy. The present arsenal of drugs available against leishmaniasis is rather small, and moreover, these drugs are limited by disadvantages such as the requirement for prolonged treatments, the need for parenteral routes of administration, high toxicity in many cases and the fact that they are becoming increasingly ineffective due to the appearance of resistant forms of the parasite [6]. Although the global leishmaniasis incidence has remained relatively stable over the past decade, a major problem exists in the immunocompromised population, often co-infected with other NTSs or with the human immunodeficiency virus (HIV) [7,8,9], and thus, due to the increasing numbers of HIV patients, leishmaniasis chemotherapy is further complicated by variations in efficacy associated with immune status. In this context, tuberculosis is also an immunosuppressive condition that affects the treatment of leishmaniasis, which depends on an effective immune response that activates macrophages. Furthermore, tuberculosis may cause progression of latent leishmaniasis to the clinical stage, while visceral leishmaniasis can also reactivate latent tuberculosis. Thus, pulmonary tuberculosis and leishmaniasis (as well as other parasitic diseases) have been shown to be risk factors for each other [10] and due to their geographical overlap, co-infections are very common [11,12,13,14]. The low level of development of the countries with the highest prevalence of both infections makes the simultaneous treatment of these diseases difficult. Therefore, there is an urgent need for affordable new therapies with integrated dual activity against both infections. If successful, these compounds can be regarded as a particular case of multi-target directed ligands, one of the most promising current paradigms in drug discovery [15].

**Figure 1:**
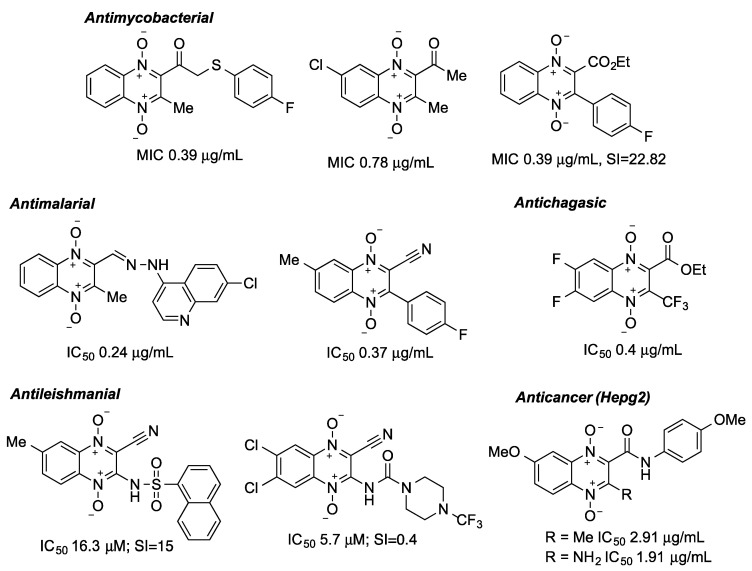
Representative examples of QdNOs with diverse biological activities.

Heterocyclic structures play a crucial role in medicinal chemistry as powerful tools functioning both as therapeutics and as probes, with special emphasis being paid to nitrogen compounds [16,17]. Particularly, quinoxaline-1,4-dioxide (QdNO) is a privileged scaffold with manifold biological properties [18,19,20,21]. Some drugs belonging to the QdNO class of compounds include carbadox, olaquindox, quinocetone, cyadox and mequindox. Furthermore, over the last few years, several studies have uncovered QdNO derivatives that are endowed with antituberculosis [22,23,24], antiviral [25], antichagasic [26,27], antileishmanial [28] and hypoxia-selective antitumor properties [29,30] (Figure 1).

In this context, we present a method for synthesizing a previously unexplored category of quinoxaline-1,4-dioxides that incorporate a glycine side chain, starting from the widely acknowledged 2,5-piperazinedione template [31]. The compounds thus generated were screened for their activity against *Mycobacterium tuberculosis* and were also studied as antileishmanial agents in an effort to obtain a hit compound that can be used as a starting point toward addressing the above-mentioned co-infection problem. The inclusion of amino acid structural fragments is a useful technique in drug design for the modulation of physicochemical properties and exploiting protein transporters to improve membrane permeation [32,33,34]. Furthermore, it provides opportunities for specific drug uptake by bacteria or parasite-specific proteins, which may be useful to achieve selective toxicity. Mycobacteria are characterized by the very low permeability of their wall and are therefore highly dependent on the uptake of nutrients by transporter proteins, although the identity of many of these transporters is still unknown [35].

## 2. Results 

### 2.1. Synthesis

**Scheme 1:**
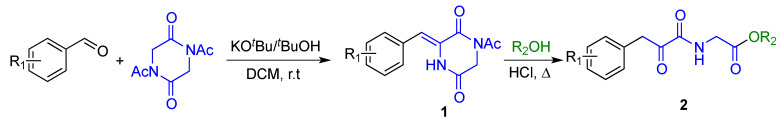
Synthesis of tricarbonyl compounds **2** from 2,5-diketopiperazine.

2,5-Diketopiperazines (DKPs) are the simplest cyclic peptides and are readily accessible from amino acids, representing highly versatile scaffolds in synthesis [31,36,37]. In this context, we [38] and others [39,40] have been actively involved in the development of new DKP-based synthetic methodologies, with a specific focus on the regioselective cleavage of suitably functionalized DKP derivatives. The synthetic strategy to obtain the QdNO compounds **4** and **5** involved a three-step sequence starting from 3-arylmethylene-2,5-piperazinediones **1**. The required compounds **1** were readily available by aldol condensation of 1,4-diacetyl-2,5-piperazinedione with substituted benzaldehydes in the presence of potassium *tert*-butoxide [41], and they were opened by acid-promoted alcoholysis of the 2,5-piperazinedione ring under microwave irradiation or reflux conditions [42] to give the *N*-(3-arylpyruvylamino) esters **2**. In order to study the effect on the bioactivity of substituents with varied electronic effects, chloro, fluoro and trifluoromethyl were used as electron-withdrawing groups, and methyl and methoxy as electron-donating groups. Yields were generally good, independently of the nature of these substituents at the aromatic ring. For the second step, methanol and benzyl alcohol were employed as nucleophiles for the regioselective opening of the DKP ring to furnish compounds **2** (Scheme 1). The scope and yields of these reactions are summarized in Table 1.

**Scheme 2:**
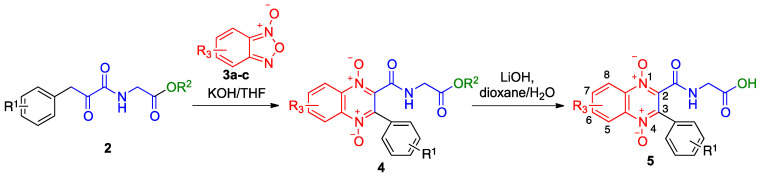
Application of the Beirut reaction to the synthesis of compounds **4** and their hydrolysis to **5**.

The target quinoxaline-1,4-dioxide framework was synthesized using a modification of the Beirut reaction [43,44,45] by treating **2a**–**i** with the suitable benzofuraxan derivatives **3**. Some of the compounds **4** thus obtained were used as starting materials for the synthesis of the acids **5**. After screening several reaction conditions, lithium hydroxide in H_2_O/dioxane ultimately provided the most satisfactory results for the saponification of **4**. As shown in Scheme 2 and Table 2, this short multi-step sequence demonstrated good tolerance for both electron-withdrawing and electron-donating groups.

**Figure 2:**
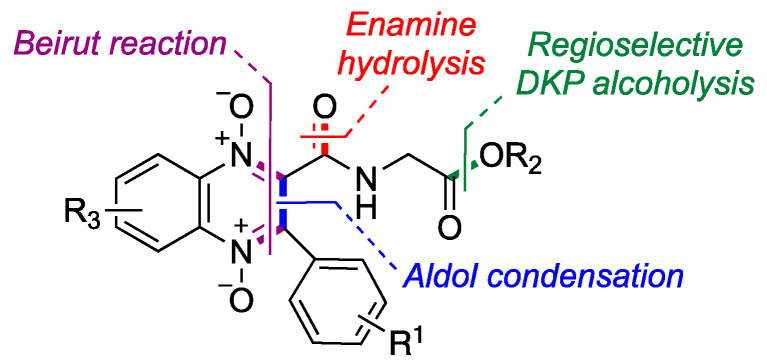
A summary of the bonds generated in our synthetic strategy.

In summary, our method represents a new entry into functionalized quinoxaline-1,4-di-*N*-oxides, generating five bonds over three steps, as summarized in Figure 2.

**Scheme 3:**
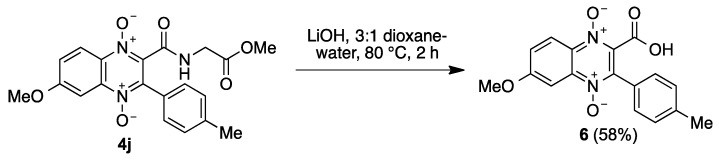
Preparation of the additional compound **6** used as a reference for the biological studies.

For reference purposes, we added to this study compound **6**, an analogue of **4j** lacking the glycine side chain. This compound was prepared by hydrolysis of the amide bond in compound **4j** by treatment with lithium hydroxide in dioxane-water (Scheme 3).

### 2.2. Biology

#### 2.2.1. Antituberculosis Activity

**Figure 3:**
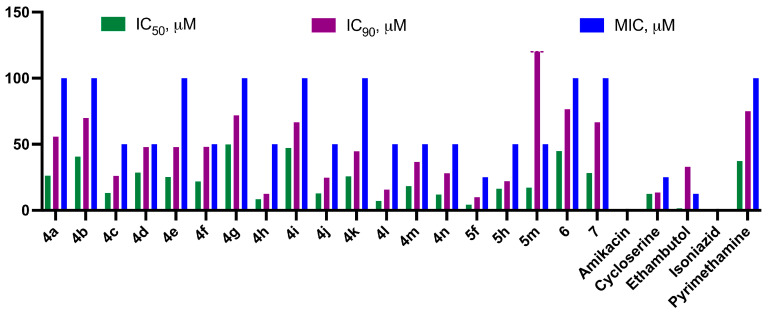
Antibacterial activity against *Mycobacterium tuberculosis* Mtb H37Rv. The numerical data corresponding to this graphic can be found in Table S1 (Supporting Information). None of the compounds showed cytotoxicity in Vero cells at concentrations up to 6.25 μg/mL.

We first examined our compounds for activity against *Mycobacterium tuberculosis* by conducting high-throughput screening of their in vitro activity against the H37Rv strain, using an assay adapted from the microdilution AlamarBlue (AB) broth assay reported by Collins and Franzblau [46]. The results obtained, expressed as IC_50_, IC_90_ and MIC, are shown in Figure 3 and Table S1. Our compounds showed IC_50_ values in the 4.28–49.95 μM range, with compounds **4c**, **4h**, **4l**, **4m**, **4n**, **5f** and **5m** showing the best inhibition results, which are comparable to those found for two antitubercular drugs in clinical use, namely, pyrimetamine (IC_50_: 37.35 μM) and cycloserine (IC_50_: 12.47 μM). Importantly, all compounds showed low cytotoxicity values against Vero cells.

#### 2.2.2. Antileishmanial Activity

**Figure 4:**
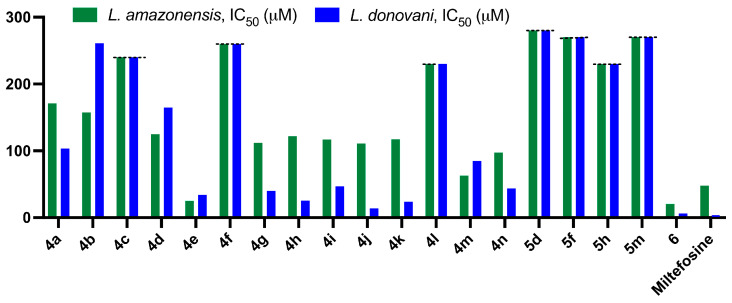
Antileishmanial activity against *Leishmania amazonensis* and *Leishmania donovani* promastigotes (IC_50_, μM). The numerical data corresponding to this graphic can be found in Table S2 (Supporting Information).

**Figure 5:**
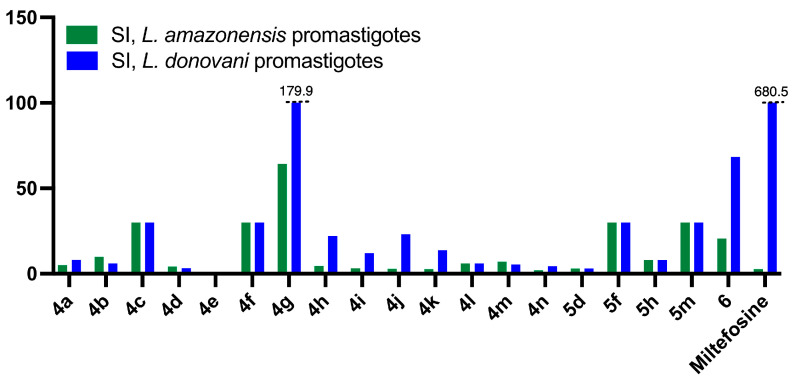
Selectivity indexes, obtained as the ratio between the toxicity against J774 macrophages (CC_50_, μM) and the IC_50_ values against *Leishmania amazonensis* and *Leishmania donovani* promastigotes (IC_50_, μM). The numerical data corresponding to this graphic can be found in Table S2 (Supporting Information).

The QdNO derivatives were also tested against promastigote forms of two *Leishmania* parasites, namely, *L. amazonensis*, which causes cutaneous and mucocutaneous leishmaniasis, and *L. donovani*, which is associated with visceral leishmaniasis. While intracellular amastigote models, which have the advantage of including the effects of the host cell, are normally regarded as the gold standard for in vitro drug discovery against *Leishmania* [47], axenic systems such as promastigotes provide a fast and convenient way to perform the initial screening of a compound library. The results obtained in the study of our compounds against *L. amazonensis* and *L. donovani* promastigotes are summarized in Figure 4 and Figure 5 and Table S2 (Supporting Information). 

**Figure 6:**
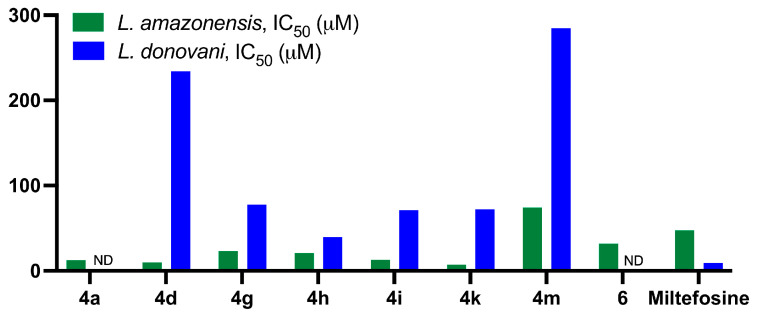
Antileishmanial activity against *Leishmania amazonensis* and *Leishmania donovani* amastigotes (IC_50_, μM). The numerical data corresponding to this graphic can be found in Table S3 (Supporting Information).

**Figure 7:**
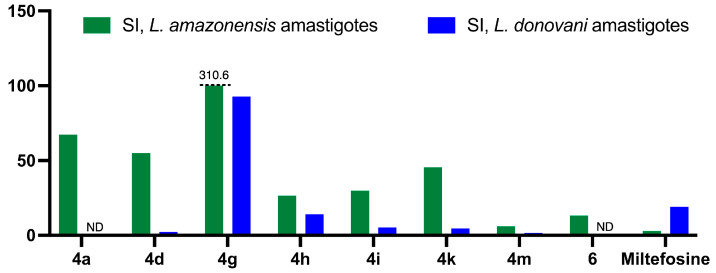
Selectivity indexes, obtained as the ratio between the toxicity against J774 macrophages (CC_50_, μM) and the IC_50_ values against *Leishmania amazonensis* and *Leishmania donovani* amastigotes (IC_50_, μM). The numerical data corresponding to this graphic can be found in Table S3 (Supporting Information).

Based on these results and in order to complete the characterization of our library, we selected compounds **4a**, **4d**, **4g**, **4h**, **4i**, **4k**, **4m** and **6** to be assayed in the intracellular amastigote model against both *L. amazonensis* and *L. donovani*, with the results shown in Figure 6 and Figure 7 and Table S3 (Supporting Information).

## 3. Discussion

Regarding antitubercular structure–activity relationships, the presence of substituents on the aromatic ring in the C-3 position of the quinazoline skeleton increases activity (compare **4a**/**4c** with **4d** and **4f**/**4h** with **4f**). In all cases, compounds bearing this substituent in the *para* position showed better results than their *ortho* or *meta* analogues (compare **4j**/**4k** and **4b** with **4g**). Additionally, the presence of a substituent at the C-6 position of the quinoxaline ring led to an increase in the activity (compare **4a**/**4f** and **4n**; **4l**/**4c** and **4d**/**4m**), and similar results were observed for methyl and methoxy groups (compare **4i**/**4m** and **4h**/**4l**). The presence of the trifluoromethyl group in any of the rings improved the inhibition results (**4c**, **4h** and **4l**). Regarding the glycine side chain, the presence of the carboxylic acid seemed to increase activity compared to the methyl ester (compare compounds **4f**/**5f** and **4i**/**5i**). Compound **6**, with a carboxylic group attached directly to the C-2 position of the heterocyclic skeleton, showed the lowest activity of the whole series, showcasing the relevance of the glycine side chain for antitubercular activity.

Compounds **4c**, **4f**, **4l** and their derivatives **5**, containing a carboxylic acid group at the side chain, were inactive against both *Leishmania* species. An analysis of the results obtained against *L. amazonensis* showed that four of the compounds (**4e**, **4m**, **4n** and **6**) exhibited similar in vitro activity to the drug miltefosine, used as a positive control, with IC_50_ values in the 20–97 μM range (miltefosine gave 47.9 μM). Unfortunately, compound **4e** was highly toxic to macrophages, and therefore, it showed very little selectivity (SI = 0.7). The presence of a para substituted phenyl at C-3 increased activity compared to unsubstituted phenyl (compare **4a**/**4d**/**4b** with **4f**/**4h/4i**/**4j**). Electronic effects were not relevant for the activity values (compare **4a**/**4b**/**4d**, **4g**/**4h**/**4i**/**4k** and **4n**/**4m**/**4l**). The presence of a substituent at C-6 increases the activity, and electron-withdrawing groups are preferred, since CF_3_ leads to a higher activity than CH_3_ and the latter than MeO (compare **4n**/**4f**/**4a** with **4i**/**4m**). The ester group in the glycine side chain increased activity compared to the corresponding carboxylic acid. Finally, compound **6**, bearing a carboxylic group at C-2, showed a slightly increased activity, suggesting that the glycine side chain is counterproductive in terms of antileishmanial activity in the promastigote stage. Nevertheless, its key role in terms of antitubercular properties makes it an essential structural feature for the desired dual activity.

An analysis of the results obtained against *L. donovani* revealed lower inhibition activity in comparison to miltefosine. However, eleven of the tested compounds exhibited interesting inhibition and low toxicity results. An analysis of the structure–activity relationships led to the same conclusions as those of the *L. amazonensis* study. The esters showed better results than their carboxylic acid analogues and the C-3-phenyl-substituted phenyls compounds performed better than their unsubstituted analogues. On the subject of the substituents in the C-6 position, although the electronic effect of the substituent was not relevant for the activity, the presence of a methoxy group increased the selectivity index (compounds **4g**, **4h**, **4i**, **4j** and **4k**).

Compound **4g** showed the best SI value (310.6) and the presence of the methoxy group in compound **4d** (IC_50_ = 9.8 μM; SI = 54) improved the potency and the selectivity index compared to methyl analogue **4m** (IC_50_ = 74.4 μM; SI = 6.1). The QNOs **4k** and **4d** (in this order) were the two most potent compounds in these experiments. Furthermore, **4k** (IC_50_ = 7.2 μM; SI = 330) and **4d** (IC_50_ = 9.8 μM; SI = 539) were considerably more potent and less toxic than the reference antileishmanial drug miltefosine (IC_50_ = 136.1 μM; SI = 2.87). A similar study was performed with *L. donovani* amastigotes, showing lower activities for all compounds and suggesting that they are more promising for cutaneous and mucocutaneous leishmaniasis. Nevertheless, **4h** maintained a respectable activity against *Leishmania donovani* amastigotes and can thus be regarded as the compound with a better-rounded antileishmanial profile.

As an overall conclusion, the best-balanced dual antitubercular/antileishmanial profile corresponds to compound **4h**. It is relevant to note that this compound shows activities in the same order of magnitude for all organisms assayed, a very important feature for a drug aimed at several targets, and can therefore be proposed as a hit for further development.

## 4. Materials and Methods

### 4.1. Synthesis

#### 4.1.1. General Synthetic Experimental Information

NMR spectra were registered on a Bruker Avance 250 spectrometer (250 MHz for ^1^H, 63 MHz for ^13^C) (Bruker, Rivas-Vaciamadrid, Spain) and maintained by the Unidad de Resonancia Magnética Nuclear, UCM. Infrared spectra were obtained by preparing KBr disks for each compound and registering their spectra on a Perkin Elmer Paragon 1000 FT-IR spectrophotometer (Tres Cantos, Spain). Melting points were taken on a Reichert 723 hot stage microscope (Vienna, Austria). CHN combustion elemental analyses were performed by the Unidad de Microanálisis Elemental, UCM, using a Leco CHNS-932 combustion microanalyzer (Tres Cantos, Spain).

#### 4.1.2. General Procedure for the Synthesis of 3-Arylmethylene-2,5-Piperazinediones (**1**)

A solution of 1,4-diacetyl-2,5-piperazinedione (4.41 mmol) and the corresponding aldehyde (4.41 mmol) in DCM (12 mL) was treated dropwise with a 1M solution of potassium *tert*-butoxide in *tert*-butyl alcohol (4.41 mL). The solution was stirred at room temperature for 5 h. Then, 10 mL of a saturated aqueous solution of NH_4_Cl was added to the reacting mixture, and the formed solid was collected by filtration and dried to obtain compounds **1**. Their characterization data are given below.

(*Z*)-1-acetyl-3-benzylidene-2,5-piperazinedione (**1a**). This compound, isolated in 82% yield as a white solid following the general procedure, was known in the literature [42]. Mp: 200–201 °C (lit. 198–200 °C). ^1^H NMR (250 MHz, CDCl_3_) δ: 8.05 (s, 1H), 7.61–7.36 (m, 5H), 7.21 (s, 1H), 4.55 (s, 2H) and 2.69 (s, 3H). ^13^C NMR (63 MHz, CDCl_3_) δ: 172.6, 162.9, 160.1, 132.6, 129.7, 129.5, 128.7, 125.8, 120.1, 46.2 and 27.4. IR (neat, NaCl) ν: 1695 and 1625 cm^−1^.

(*Z*)-1-Acetyl-3-(4-trifluoromethylphenylmethylene)-2,5-piperazinedione (**1b**). This compound was obtained in 73% yield as a yellow solid following the general procedure. Mp: 121–123 °C. ^1^H NMR (250 MHz, CDCl_3_) δ: 7.94 (s, 1H), 7.76 (d, *J* = 8.2 Hz, 2H), 7.54 (d, *J* = 8.0 Hz, 2H), 7.20 (s, 1H), 4.56 (s, 3H) and 2.70 (s, 3H). ^13^C NMR (63 MHz, CDCl_3_) δ: 172.83, 163.63, 160.02, 136.51, 131.2 (q, *^2^J* (*C*,*F*) = 32.8Hz), 129.44, 127.49, 126.8 (q, *^4^J* (*C*,*F*) = 3.8 Hz), 118.54, 46.45 and 27.66. IR (neat, NaCl) ν: 3252, 1699, 1635 and 1440 cm^−1^. Analysis: Calculated for C_14_H_11_F_3_N_2_O_3_: C, 53.85; H, 3.55; N, 8.97. Found: C, 52.90; H, 3.81; N, 8.55.

(*Z*)-1-acetyl-3-(4-fluorophenylmethylene)-2,5-piperazinedione (**1c**). This compound was obtained in 72% yield as a white solid following the general procedure. ^1^H NMR (250 MHz, CDCl_3_) δ: 7.84 (s, 1H), 7.47–7.33 (m, 2H), 7.22–7.07 (m, 3H), 4.52 (s, 2H) and 2.66 (s, 3H). ^13^C NMR (63 MHz, MeOD) δ: 173.9, 171.6, 166.5, 163.4 (d, *^1^J* (*C*,*F*) = 156.3 Hz), 162.6, 132.6 (d, *^3^J* (*C*,*F*) = 8.4 Hz), 130.8, 127.7, 120.1, 116.9 (d, *^2^J* (*C*,*F*) = 22.0 Hz), 46.9 and 27.1. IR (neat, NaCl) ν: 3250, 1702, 1687 and 1627 cm^−1^. Analysis: Calculated for C_13_H_11_F_1_N_2_O_3_: C, 59.54; H, 4.23; N, 10.68. Found: C, 58.63; H, 4.58; N, 11.02.

(*Z*)-1-Acetyl-3-(3-chlorophenylmethylene)-2,5-piperazinedione (**1d**). This compound was obtained in 97% yield as a white solid following the general procedure. Mp: 202–204 °C. ^1^H NMR (250 MHz, CDCl_3_) δ: 7.41 (m, 2H), 7.29 (s, 2H), 7.14 (s, 1H), 4.55 (s, 2H) and 2.69 (s, 3H). ^13^C NMR (63 MHz, CDCl_3_) δ: 172.6, 163.0, 159.8, 135.7, 134.4, 131.0, 129.6, 128.7, 126.7, 118.4, 108.71, 46.2 and 27.4. IR (neat, NaCl) ν: 3224, 1696, 1679 and 1635 cm^−1^. Analysis: Calculated for C_13_H_11_ClN_2_O_3_: C, 56.03; H, 3.98; N, 10.05. Found: C, 43.55; H, 3.89; N, 10.13.

(*Z*)-1-Acetyl-3-(4-methylphenylmethylene)-2,5-piperazinedione (**1e**). This compound, isolated in 65% yield as a white solid following the general procedure, was known in the literature [48]. ^1^H NMR (250 MHz, CDCl_3_) δ: 7.92 (s, 1H), 7.30 (d, *J* = 8.7 Hz, 2H), 7.29 (d, *J* = 8.7 Hz, 2H), 7.16 (s, 2H), 4.51 (s, 2H), 2.65 (s, 3H) and 2.39 (s, 3H). ^13^C NMR (63 MHz, CDCl_3_) δ: 172.69, 162.85, 160.25, 140.02, 130.43, 129.71, 128.68, 125.15, 120.40, 46.24, 27.38 and 21.58. IR (neat): 3271, 1697, 1678, 1628 and 1355 cm^−1^. Analysis: Calculated for C_14_H_14_N_2_O_3_: C, 65.11; H, 5.43; N, 10.85. Found: C, 64.67; H, 5.32; N, 10.81.

(*Z*)-1-Acetyl-3-(2-methylphenylmethylene)-2,5-piperazinedione (**1f**). This compound, isolated in 50% yield as a white solid following the general procedure, was known in the literature [49]. ^1^H NMR (250 MHz, CDCl_3_) δ: 7.91 (s, 1H), 7.40–7.23 (m, 5H), 4.51 (s, 2H), 2.70 (s, 3H) and 2.34 (s, 3H). ^13^C NMR (63 MHz, CDCl_3_) δ: 172.7, 162.7, 159.8, 137.8, 131.4, 131.3, 129.5, 127.5, 126.8, 126.2, 119.4, 46.3, 27.5 and 20.1. IR (neat): 3194, 1691, 1663 and 1621 cm^−1^. Analysis: Calculated for C_14_H_14_N_2_O_3_: C, 65.11; H, 5.43; N, 10.85. Found: C, 64.72; H, 5.28; N, 10.66.

(*Z*)-1-Acetyl-3-(4-Methoxyphenylmethylene)-2,5-piperazinedione (**1g**). This compound, isolated in 57% yield as a yellow solid following the general procedure, was known in the literature [50]. Mp: 189–192 °C. ^1^H NMR (250 MHz, CDCl_3_) δ: 8.01 (s, 1H), 7.38 (t, *J* = 7.9 Hz, 1H), 7.14 (s, 1H), 6.97 (dd, *J* = 8.0, 1.1 Hz, 1H), 6.94–6.85 (m, 2H), 4.51 (s, 2H), 3.83 (s, 3H) and 2.65 (s, 3H). ^13^C NMR (63 MHz, CDCl_3_) δ: 172.7, 162.8, 160.4, 160.0, 133.9, 130.8, 126.0, 120.6, 119.9, 114.9, 114.5, 55.5, 46.3 and 27.4. IR (neat, NaCl) ν: 3214, 1699, 1684, 1629 and 1429 cm^−1^. Analysis: Calculated for C_14_H_14_N_2_O_4_: C, 61.31; H, 5.14; N, 10.21. Found: C, 60.71; H, 5.12; N, 10.13.

(*Z*)-1-Acetyl-3-(5,6-dimethoxy-2-nitrophenylmethylene)-2,5-piperazinedione (**1h**). This compound was obtained in 80% yield as a yellow solid following the general procedure. Mp: 198–200 °C. ^1^H NMR (250 MHz, CDCl_3_) δ: 8.27 (s, 1H), 7.78 (s, 1H), 7.43 (s, 1H), 6.74 (s, 1H), 4.42 (s, 2H), 3.99 (s, 3H), 3.97 (s, 3H) and 2.66 (s, 3H). ^13^C NMR (63 MHz, CDCl_3_) δ: 172.7, 163.3, 159.3, 154.0, 149.5, 140.7, 126.5, 122.3, 118.2, 111.2, 109.0, 56.9, 56.7, 46.2 and 27.4. IR (neat, NaCl) ν: 3345, 3179, 1680 and 1631 cm^−1^. Analysis: Calculated for C_15_H_15_N_3_O_7_: C, 51.58; H, 4.33; N, 12.03. Found: C, 51.36; H, 4.21; N, 11.87.

#### 4.1.3. General Procedure for the Synthesis of Alkyl 2-(2-Oxo-3-Arylpropanamido)Acetate Derivatives (**2**)

To a stirred solution of the corresponding arylmethylene-2,5-piperazinedione **1** (6 mmol) in alcohol solution (250 mL) was added a 5M HCl aqueous solution (25 mL). The reaction was refluxed for 16 h. When no starting material was evident by TLC, the solvent was distilled under vacuum to dryness, and the residue was dissolved in chloroform and filtered. The solvent was evaporated to give the corresponding compound **2**. Their characterization data are given below.

Methyl 2-(2-oxo-3-phenylpropanamido)acetate (**2a**). This compound was obtained in 71% yield as a yellow oil following the general procedure. ^1^H NMR (250 MHz, CDCl_3_) δ: 7.41–7.23 (m, 5H), 4.25 (s, 2H), 4.11 (d, *J* = 5.7 Hz, 2H) and 3.80 (s, 3H). ^13^C NMR (63 MHz, CDCl_3_) δ: 195.3, 169.6, 160.4, 132.8, 130.2, 129.1, 127.7, 53.4, 43.5 and 41.4. IR (neat, NaCl) ν: 3384, 1745, 1691 and 1540 cm^−1^. Analysis: Calculated for C_12_H_13_NO_4_: C, 61.27; H, 5.57; N, 5.95. Found: C, 61.45; H, 5.72; N, 6.12.

Methyl 2-(2-oxo-3-(4-(trifluoromethyl)phenyl)propanamido)acetate (**2b**). This compound was obtained in 68% yield as a yellow oil following the general procedure. ^1^H NMR (250 MHz, CDCl_3_) δ: 7.60 (d, *J* = 8.0 Hz, 2H), 7.38 (d, *J* = 8.0 Hz, 2H), 4.31 (s, 2H), 4.11 (d, *J* = 5.8 Hz, 2H) and 3.79 (s, 3H). ^13^C NMR (63 MHz, CDCl_3_) δ: 194.4, 169.3, 159.9, 136.7, 130.5, 128.7 (q, *^1^J* (*C*,*F*) = 258.2 Hz), 125.7 (q, *^3^J* (*C*,*F*) = 7.6 Hz), 52.7, 43.4 and 41.1. IR (neat, NaCl) ν: 3363, 1748, 1682 and 1591 cm^−1^. Analysis: Calculated for C_13_H_12_F_3_NO_4_: C, 51.94; H, 3.99; N, 4.62. Found: C, 48.85; H, 3.87; N, 4.55.

Methyl 2-(2-oxo-3-(4-(fluorophenyl)propanamido)acetate (**2c**). This compound was obtained in 45% yield as a yellow oil following the general procedure. ^1^H NMR (250 MHz, CDCl_3_) δ: 7.43 (s, 1H), 7.20 (dd, *J* = 8.8, 5.3 Hz, 2H), 7.08–6.93 (m, 2H), 4.19 (s, 2H), 4.08 (d, *J* = 5.7 Hz, 2H) and 3.77 (s, 3H). ^13^C NMR (63 MHz, CDCl_3_) δ: 194.88 (s), 169.30 (s), 162.23 (d, *^1^J* (*C*,*F*) = 245.8 Hz), 160.01 (s), 131.54 (d, *^3^J* (*C*,*F*) = 8.1 Hz), 128.16 (d, *^4^J* (*C*,*F*) = 3.3 Hz), 115.73 (d, *^2^J* (*C*,*F*) = 21.5 Hz), 52.77 (s), 42.43 (s) and 41.12 (s). IR (neat, NaCl) ν: 3373, 2955, 1751, 1684 and 1510 cm^−1^. Analysis: Calculated for C_12_H_12_FNO_4_: C, 56.92; H, 4.78; N, 5.53. Found: C, 58.65; H, 4.57; N, 5.35.

Methyl 2-(3-(4-chlorophenyl)-2-oxopropanamido)acetate (**2d**). This compound was obtained in 98% yield as a brown oil following the general procedure. ^1^H NMR (250 MHz, CDCl_3_) δ: 7.38 (s, 1H), 7.17–7.05 (m, 2H), 6.98 (m, 2H), 4.05 (s, 2H), 3.94 (d, *J* = 5.8 Hz, 2H) and 3.62 (s, 3H). ^13^C NMR (63 MHz, CDCl_3_) δ: 194.8, 169.6, 160.3, 134.8, 130.3, 130.3, 128.5, 127.9, 53.04, 43.1 and 41.4. IR (neat, NaCl) ν: 3384, 2961, 1747, 1690 and 1572 cm^−1^. Analysis: Calculated for C_12_H_12_ClNO_4:_ C, 53.44; H, 4.49; N, 5.19. Found: C, 53.76; H, 4.78; N, 5.86.

Methyl 2-(3-(4-methylphenyl)-2-oxopropanamido)acetate (**2e**). This compound was obtained in 54% yield as a brown oil following the general procedure. ^1^H NMR (250 MHz, CDCl_3_) δ: 7.17 (s, 4H), 4.21 (s, 2H), 4.11 (d, *J* = 5.7 Hz, 2H), 3.80 (s, 3H) and 2.36 (s, 3H). ^13^C NMR (63 MHz, CDCl_3_) δ: 195.5, 169.3, 160.1, 137.1, 129.8, 129.6, 129.3, 52.7, 42.9, 41.1 and 21.2. IR (neat, NaCl) ν: 3394, 2951, 1674 and 1515 cm^−1^. Analysis: Calculated for C_13_H_15_NO_4_: C, 62.65; H, 6.07; N, 5.62. Found: C, 62.85; H, 6.17; N, 5.55.

Methyl 2-(3-(2-methylphenyl)-2-oxopropanamido)acetate (**2f**). This compound was obtained in 55% yield as a brown oil following the general procedure. ^1^H NMR (250 MHz, CDCl_3_) δ: 7.43 (s, 1H), 7.23–7.11 (m, 4H), 4.26 (s, 2H), 4.10 (d, *J* = 5.7 Hz, 2H), 3.78 (s, 3H) and 2.25 (s, 3H). ^13^C NMR (63 MHz, CDCl_3_) δ: 195.1, 169.3, 160.1, 137.3, 131.5, 130.7, 130.6, 127.8, 126.3, 52.8, 41.3, 41.1 and 19.8. IR (neat, NaCl) ν: 3297, 2927, 1673 and 1532 cm^−1^. Analysis: Calculated for C_13_H_15_NO_4_: C, 62.65; H, 6.07; N, 5.62. Found: C, 62.58; H, 6.12; N, 5.72.

Methyl 2-(3-(4-methoxyphenyl)-2-oxopropanamido)acetate (**2g**). This compound was obtained in 40% yield as an orange oil following the general procedure. ^1^H NMR (250 MHz, CDCl_3_) δ: 7.44 (s, 1H), 7.27 (td, *J* = 7.7, 0.8 Hz, 1H), 6.90–6.79 (m, 3H), 4.22 (s, 2H), 4.11 (d, *J* = 5.7 Hz, 2H), 3.82 (s, 3H) and 3.80 (s, 3H). ^13^C NMR (63 MHz, CDCl_3_) δ: 194.9, 169.3, 160.1, 159.9, 133.9, 129.8, 122.3, 115.5, 113.0, 55.3, 52.7, 43.2 and 41.1. IR (neat, NaCl) ν: 3379, 2950, 1753, 1692 and 1601 cm^−1^. Analysis: Calculated for C_13_H_15_NO_5_: C, 58.68; H, 5.70; N, 5.28. Found: C, 58.55; H, 5.37; N, 4.77.

Methyl (3-(4,5-dimethoxy-2-nitrophenyl)-2-oxopropamido)acetate (**2h**). This compound was obtained in 68% yield as a brown solid following the general procedure. ^1^H NMR (250 MHz, CDCl_3_) δ: 7.77 (s, 1H), 7.39 (t, *J* = 5.7 Hz, 1H), 6.68 (s, 1H), 4.60 (s, 2H), 4.14 (d, *J* = 5.7 Hz, 2H), 3.97 (s, 3H), 3.96 (s, 3H) and 3.79 (s, 3H). ^13^C NMR (63 MHz, CDCl_3_) δ: 193.6, 169.3, 160.1, 153.6, 148.4, 140.7, 124.6, 114.8, 108.6, 56.6, 56.5, 52.8, 43.0 and 41.2. IR (neat, NaCl) ν: 3370, 2937, 1753 and 1678 cm^−1^. Mp: 138–140 °C. Analysis: Calculated for C_14_H_16_N_2_O_8_: C, 49.42; H, 4.74; N, 8.23. Found: C, 48.455; H, 4.37; N, 8.77.

Benzyl (3-(4,5-dimethoxy-2-nitrophenyl)-2-oxopropamido)acetate (**2i**). This compound was obtained in 63% yield as a brown solid following the general procedure. ^1^H NMR (250 MHz, CDCl_3_) δ: 7.77 (s, 1H), 7.37 (s, 5H), 6.68 (s, 1H), 5.22 (s, 2H), 4.59 (s, 2H), 4.17 (d, *J* = 5.7 Hz, 2H), 3.97 (s, 3H) and 3.96 (s, 3H). ^13^C NMR (63 MHz, CDCl_3_) δ: 193.6, 168.7, 160.1, 153.6, 148.4, 140.7, 135.0, 128.8, 128.8, 128.6, 124.6, 114.8, 108.6, 67.7, 56.6, 56.5, 43.0 and 41.4. IR (neat, NaCl) ν: 3373, 3128, 3033, 1746 and 1685 cm^−1^. Analysis: Calculated for C_20_H_20_N_2_O_8:_ C, 57.69; H, 4.84; N, 6.73. Found: C, 58.05; H, 4.97; N, 6.26.

#### 4.1.4. General Procedure for Preparation of Benzofurazane Oxides **3a**–**c**

Benzofurazan oxide **3a** and its derivatives were synthesized according to a modified literature procedure [51]. Derivatives of *o*-nitroaniline (43.5 mmol) were dissolved in 20% ethanol/KOH (250 mL). The deep red solution was cooled in an ice bath (0–5 °C). Commercial aqueous NaOCl (Clorox) (200 mL) was added with stirring until no *o*-nitroaniline was present in the solution, that is, when the solution no longer changed color from yellow to red upon the addition of NaOCl. The bright yellow precipitate was collected by suction filtration and was washed with cold water. The solid was dried under vacuum to yield yellow solid benzofurazan oxides **3a**–**3c** (65–76% yield).

#### 4.1.5. General Procedure for Preparation of 3-arylquinoxaline-1,4-di-N-oxides **4**

The suitable arylpiruvyl ester **2** (0.42 mmol) was added to a solution of the suitable benzofuranoxane **3** (0.5 mmol) and KOH (0.5 mmol) in dry THF (10 mL). The reaction mixture was stirred at room temperature for 3–16 h, and when no starting material was evident by TLC, 2 mL of a 1M HCl solution was added. The reaction mixture was extracted with AcOEt (2 × 10 mL). The organic layer was washed with brine and dried over anhydrous sodium sulfate and the solvent was evaporated under reduced pressure. The residue was crystallized from chloroform–hexane to give the pure quinoxaline-1,4-di-N-oxides **4**.

*N*-(1,4-Dioxide-3-phenylquinoxaline-2-carbonyl)glycine methyl ester (**4a**). This compound was obtained in 82% yield as a brown solid following the general procedure. ^1^H NMR (250 MHz, CDCl_3_) δ: 8.53–8.40 (m, 2H), 8.36 (s, 1H), 7.82 (m, 2H), 7.73–7.58 (m, 2H), 7.58–7.44 (m, 3H), 4.06 (d, *J* = 5.2 Hz, 2H) and 3.71 (s, 3H). ^13^C NMR (63 MHz, CDCl_3_) δ: 169.4, 159.0, 141.5, 138.0, 137.3, 136.9, 132.8, 132.5, 130.7, 129.7, 128.8, 128.3, 120.8, 120.0, 52.6 and 41.7. IR (neat, NaCl) ν: 3414, 2926, 2856 and 1666 cm^−1^. Mp: 180–183 °C. Analysis: Calculated for C_18_H_15_N_3_O_5:_ C, 61.19; H, 4.28; N, 11.89. Found: C, 61.66; H, 4.16; N, 11.40.

*N*-(1,4-Dioxide-3-((4-methoxyphenyl)quinoxaline-2-carbonyl)glycine methyl ester (**4b**). This compound was obtained in 98% yield as a yellow solid following the general procedure. ^1^H NMR (250 MHz, CDCl_3_) δ: 8.49–8.42 (m, 2H), 8.38 (t, *J* = 5.4 Hz, 1H), 7.82 (dd, *J* = 6.6, 3.4 Hz, 2H), 7.48–7.40 (m, 1H), 7.24 (m, 1H), 7.23–7.17 (m, 1H), 7.06 (m, 1H), 4.06 (d, *J* = 5.3 Hz, 2H), 3.84 (s, 3H) and 3.71 (s, H). ^13^C NMR (63 MHz, CDCl_3_) δ: 169.7, 159.9, 159.2, 141.5, 138.2, 138.1, 137.0, 133.0, 132.9, 130.2, 129.3, 122.3, 121.0, 120.2, 117.2, 115.2, 55.8, 52.9 and 41.9. IR (neat, NaCl) ν: 3298, 2948, 2922, 1727, 1681 and 1342 cm^−1^. Mp: 75–77 °C. Analysis: Calculated for C_19_H_17_N_3_O_6:_ C, 59.53; H, 4.47; N, 10.96. Found: C, 60.06; H, 4.75; N, 10.40.

*N*-(1,4-Dioxide-3-((4-trifluoromethylphenyl)quinoxaline-2-carbonyl)glycine methyl ester (**4c**). This compound was obtained in 78% yield as a yellow solid following the general procedure. ^1^H NMR (250 MHz, CDCl_3_) δ: 8.72 (t, *J* = 4.6 Hz, 1H), 8.67–8.56 (m, 2H), 7.94 (dq, *J* = 6.9, 3.4 Hz, 2H), 7.81 (d, *J* = 8.3 Hz, 2H), 7.74 (d, *J* = 8.3 Hz, 2H), 4.13 (d, *J* = 5.2 Hz, 2H) and 3.75 (s, 3H). ^13^C NMR (63 MHz, CDCl_3_) δ: 169.7, 158.9, 141.1, 138.5, 137.6, 136.7, 133.6, 133.3, 132.8, 130.5, 126.1, 126.0, 121.2, 120.9, 53.0 and 41.9. IR (neat, NaCl) ν: 3290, 1749, 1679, 1530 and 1389 cm^−1^. Mp: 175–177 °C. Analysis: Calculated for C_19_H_14_F_3_N_3_O_5:_ C, 54.16; H, 3.35; N, 9.97. Found: C, 53.89; H, 3.76; N, 10.23.

*N*-(1,4-Dioxide-3-((4-fluorophenyl)quinoxaline-2-carbonyl)glycine methyl ester (**4d**). This compound was obtained in 74% yield as a yellow solid following the general procedure. ^1^H NMR (250 MHz, CDCl_3_) δ: 8.73–8.57 (m, 2H), 8.18 (t, *J* = 5.1 Hz, 1H), 8.01–7.86 (m, 2H), 7.65–7.53 (m, 2H), 7.23 (d, *J* = 8.8 Hz, 1H), 7.19 (d, *J* = 8.8 Hz, 1H), 4.13 (d, *J* = 5.1 Hz, 2H) and 3.76 (s, 3H). ^13^C NMR (63 MHz, CDCl_3_) δ: 169.4 (s), 158.8 (s), 140.1 (d, *J* = 86.0 Hz), 138.1 (s), 137.5 (s), 136.9 (s), 133.0 (s), 132.7 (s), 132.2 (d, *J* = 8.5 Hz), 124.3–123.9 (m), 120.8 (s), 120.3 (s), 116.1 (d, *J* = 22.6 Hz), 52.7 (s) and 41.6 (s). IR (neat, NaCl) ν: 3317, 2360, 1715, 1651 and 1389 cm^−1^. Mp: 175–177 °C. Analysis: Calculated for C_19_H_14_F_3_N_3_O_5:_ C, 54.16; H, 3.35; N, 9.97. Found: C, 53.89; H, 3.76; N, 10.23.

*N*-(1,4-Dioxide-3-((4,5-dimethoxy-2-nitro-phenyl)quinoxaline-2-carbonyl)glycine benzyl ester (**4e**). This compound was obtained in 83% yield as a yellow solid following the general procedure. ^1^H NMR (250 MHz, CDCl_3_) δ: 9.92 (t, *J* = 5.2 Hz, 1H), 8.72 (dd, *J* = 6.6, 3.3 Hz, 1H), 8.60 (dd, *J* = 6.6, 3.3 Hz, 1H), 8.01–7.91 (m, 2H), 7.90 (s, 1H), 7.40–7.28 (m, 5H), 6.76 (d, *J* = 10.1 Hz, 1H), 5.17 (d, *J* = 12.3 Hz, 1H), 5.11 (d, *J* = 12.2 Hz, 1H), 4.21 (dd, *J* = 18.3, 5.5 Hz, 1H), 4.15–4.05 (m, 1H), 4.02 (s, 3H) and 3.93 (s, 3H). ^13^C NMR (63 MHz, CDCl_3_) δ: 168.7, 158.6, 154.5, 149.7, 141.9, 140.5, 137.9, 137.5, 135.1, 134.0, 133.4, 132.9, 131.0, 128.8, 128.7, 128.5, 121.1, 120.8, 120.4, 111.5, 108.0, 67.5, 56.7, 56.6 and 41.7. IR (neat, NaCl) ν: 3299, 2920, 2849, 1742, 1675 and 1510 cm^−1^. Mp: 175–177 °C. Analysis: Calculated for C_19_H_14_F_3_N_3_O_5:_ C, 54.16; H, 3.35; N, 9.97. Found: C, 53.89; H, 3.76; N, 10.23.

*N*-(6-Methoxy-1,4-dioxide-3-(phenyl)quinoxaline-2-carbonyl)glycine benzyl ester (**4f**). This compound was obtained in 76% yield as a brown solid following the general procedure. ^1^H NMR (250 MHz, CDCl_3_) δ: 8.53 (d, *J* = 9.5 Hz, 1H), 8.26 (t, *J* = 4.5 Hz, 1H), 7.92 (d, *J* = 2.7 Hz, 1H), 7.59–7.44 (m, 6H), 4.09 (d, *J* = 5.1 Hz, 2H), 4.01 (s, 3H) and 3.73 (s, 3H). ^13^C NMR (63 MHz, CDCl_3_) δ: 169.4, 163.6, 158.9, 142.2, 139.5, 135.9, 132.0, 130.7, 129.7, 128.7, 128.2, 124.8, 121.6, 99.2, 56.7, 52.6 and 41.6. IR (neat, NaCl) ν: 3313, 3073, 2954, 2360, 1746 and 1681 cm^−1^. Mp: 175–177 °C. Analysis: Calculated for C_19_H_14_F_3_N_3_O_5:_ C, 54.16; H, 3.35; N, 9.97. Found: C, 53.89; H, 3.76; N, 10.23.

*N*-(6-Methoxy-1,4-dioxide-3-(3-chlorophenyl)quinoxaline-2-carbonyl)glycine methyl ester (**4g**). This compound was obtained in 79% yield as a yellow solid following the general procedure. ^1^H NMR (250 MHz, CDCl_3_) δ: 8.62 (t, *J* = 4.2 Hz, 1H), 8.25 (d, *J* = 8.3 Hz, 1H), 7.66 (s, 1H), 7.48 (d, *J* = 1.4 Hz, 1H), 7.35 (m, 4H), 3.97 (d, *J* = 5.1 Hz, 2H), 3.88 (s, 3H) and 3.62 (s, 3H). ^13^C NMR (63 MHz, CDCl_3_) δ: 169.7, 164.0, 159.1, 141.3, 139.7, 135.2, 134.9, 132.4, 131.0, 130.8, 130.3, 130.0, 128.2, 125.2, 122.0, 99.6, 57.0, 52.9 and 41. IR (neat, NaCl) ν: 3285, 1738, 1661 and 1614 cm^−1^. Mp: 175–177 °C. Analysis: Calculated for C_19_H_16_ClN_3_O_6:_ C, 54.62; H, 3.86; N, 10.06. Found: C, 54.78; H, 4.16; N, 10.39.

*N*-(6-Methoxy-1,4-dioxide-3-(4-trifloromethylphenyl)quinoxaline-2-carbonyl)glycine methyl ester (**4h**). This compound was obtained in 72% yield as a brown solid following the general procedure. ^1^H NMR (250 MHz, CDCl_3_) δ: 8.87 (t, *J* = 4.9 Hz, 1H), 8.53 (d, *J* = 9.5 Hz, 1H), 7.90 (d, *J* = 2.4 Hz, 1H), 7.80 (d, *J* = 8.4 Hz, 1H), 7.71 (d, *J* = 8.4 Hz, 1H), 7.51 (dd, *J* = 9.4, 2.6 Hz, 1H), 4.12 (d, *J* = 5.2 Hz, 2H), 4.03 (s, 3H) and 3.74 (s, 3H). ^13^C NMR (63 MHz, CDCl_3_) δ: 169.8, 164.2, 159.1, 141.7, 140.0, 134.8, 133.1, 132.7, 130.4, 125.9, 125.6, 122.4, 99.5, 57.0, 53.0 and 41.9. IR (neat, NaCl) ν: 3319, 1745, 1668 and 1325 cm^−1^. Mp: 177–179 °C. Analysis: Calculated for C_19_H_16_ClN_3_O_6:_ C, 53.22; H, 3.57; N, 9.31. Found: C, 48.39; H, 3.37; N, 7.52.

*N*-(6-Methoxy-1,4-dioxide-3-(4-florophenyl)quinoxaline-2-carbonyl)glycine methyl ester (**4i**). This compound was obtained in 62% yield as a brown solid following the general procedure. ^1^H NMR (250 MHz, DMSO) δ: 9.21–9.10 (m, *J* = 5.6 Hz, 1H), 8.43 (d, *J* = 9.5 Hz, 1H), 7.84 (d, *J* = 2.3 Hz, 1H), 7.70–7.55 (m, 3H), 7.34 (t, *J* = 8.9 Hz, 2H), 4.00 (s, 3H), 3.92 (d, *J* = 5.5 Hz, 2H) and 3.55 (s, 3H). ^13^C NMR (63 MHz, CDCl_3_) δ: 169.7 (s), 164.0 (s), 159.4 (s), 151.9 (d, *J* = 59.2 Hz), 139.8 (s), 135.6 (s), 132.3 (d, *J* = 8.8 Hz), 127.4 (s), 125.0 (s), 122.2 (s), 121.1 (s), 116.3 (d, *J* = 22.0 Hz), 111.8 (s), 99.6 (s), 57.0 (s), 53.9 (s) and 42.9 (s). IR (neat, NaCl) ν: 3342, 2953, 1732 and 1601 cm^−1^. Mp: 177–179 °C. Analysis: Calculated for C_19_H_16_ClN_3_O_6:_ C, 53.22; H, 3.57; N, 9.31. Found: C, 48.39; H, 3.37; N, 7.52.

*N*-(6-Methoxy-1,4-dioxide-3-(4-methylphenyl)quinoxaline-2-carbonyl)glycine methyl ester (**4j**). This compound was obtained in 35% yield as a brown solid following the general procedure. ^1^H NMR (250 MHz, CDCl_3_) δ: 8.42 (d, *J* = 9.6 Hz, 1H), 8.37 (t, *J* = 5.2 Hz, 1H), 7.83 (d, *J* = 2.5 Hz, 1H), 7.53 (d, *J* = 8.1 Hz, 2H), 7.43 (dd, *J* = 9.6, 2.4 Hz, 1H), 7.33 (d, *J* = 8.1 Hz, 2H), 4.10 (d, *J* = 5.1 Hz, 2H), 4.01 (s, 3H), 3.75 (s, 3H) and 2.45 (s, 3H). ^13^C NMR (63 MHz, CDCl_3_) δ: 169.4, 163.6, 159.2, 140.8, 139.5, 132.0, 129.5, 125.8, 124.5, 121.8, 101.9, 99.4, 56.7, 52.6, 41.7 and 21.8. IR (neat, NaCl) ν: 3321, 1743, 1670 and 1321 cm^−1^. Mp: 177–179 °C. Analysis: Calculated for C_19_H_16_ClN_3_O_6:_ C, 53.22; H, 3.57; N, 9.31. Found: C, 48.39; H, 3.37; N, 7.52.

*N*-(6-Methoxy-1,4-dioxide-3-(2-methylphenyl)quinoxaline-2-carbonyl)glycine methyl ester (**4k**). This compound was obtained in 45% yield as a brown solid following the general procedure. ^1^H NMR (250 MHz, CDCl_3_) δ: 8.56 (t, *J* = 5.0 Hz, 1H), 8.18 (d, *J* = 9.3 Hz, 1H), 7.57 (d, *J* = 1.8 Hz, 1H), 7.28–7.06 (m, 6H), 3.81 (d, *J* = 2.0 Hz, 2H), 3.78 (s, 3H), 3.49 (s, 3H) and 2.05 (s, 3H). ^13^C NMR (63 MHz, CDCl_3_) δ: 169.4, 163.4, 158.8, 143.2, 139.3, 138.4, 135.3, 132.1, 130.5, 130.2, 128.9, 128.8, 126.1, 124.5, 121.7, 99.3, 56.6, 52.5, 41.6 and 19.5. IR (neat, NaCl) ν: 3264, 2951 and 1740 cm^−1^. Mp: 177–179 °C. Analysis: Calculated for C_20_H_19_N_3_O_6:_ C, 60.45; H, 4.79; N, 10.58. Found: C, 60.20; H, 4.76; N, 10.42.

*N*-(6-Methyl-1,4-dioxide-3-(4-trifluoromethylphenyl)quinoxaline-2-carbonyl)glycine methyl ester (**4l**). This compound was obtained in 28% yield as a brown solid following the general procedure. ^1^H NMR (250 MHz, CDCl_3_) δ: 8.83 (t, *J* = 5.0 Hz, 1H), 8.51 (d, *J* = 8.8 Hz, 1H), 8.35 (s, 1H), 7.78 (d, *J* = 8.3 Hz, 2H), 7.73 (d, *J* = 8.3 Hz, 1H), 7.68 (d, *J* = 8.4 Hz, 2H), 4.11 (d, *J* = 5.2 Hz, 2H), 3.74 (s, 3H) and 2.65 (s, 3H). ^13^C NMR (63 MHz, CDCl_3_) δ: 169.4, 158.8, 149.9, 145.2, 138.0, 135.7, 135.3, 134.9, 132.7 (d, *J* = 35.4 Hz), 130.4 (d, *J* = 4.9 Hz), 125.7 (d, *J* = 12.5 Hz), 122.9 (d, *J* = 6.8 Hz), 120.5, 119.8, 52.7, 41.6 and 22.2. IR (neat, NaCl) ν: 3110, 1751, 1655, 1406 and 1323 cm^−1^. Mp: 177–179 °C. Analysis: Calculated for C_20_H_16_F_3_N_3_O_5:_ C, 55.17; H, 3.68; N, 9.65. Found: C, 46.93; H, 3.52; N, 9.68.

*N*-(6-Methyl-1,4-dioxide-3-(4-fluorophenyl)-quinoxaline-2-carbonyl)glycine methyl ester (**4m**). This compound was obtained in 60% yield as a yellow solid following the general procedure. ^1^H NMR (250 MHz, CDCl_3_) δ: 8.49 (d, *J* = 8.8 Hz, 1H), 8.36 (s, 1H), 8.35–8.25 (m, 1H), 7.71 (d, *J* = 8.8 Hz, 2H), 7.22 (d, *J* = 8.8 Hz, 2H), 7.19 (d, *J* = 8.7 Hz, 2H), 4.12 (d, *J* = 5.1 Hz, 2H), 3.75 (s, 3H) and 2.64 (s, 4H). ^13^C NMR (63 MHz, CDCl_3_) δ: 169.5, 159.0, 144.9 (d, *J* = 16.2 Hz), 136.8, 135.1, 134.7, 132.1 (d, *J* = 8.9 Hz), 120.5, 120.0, 119.7, 119.2, 116.1 (d, *J* = 22.1 Hz), 115.6, 113.6, 52.7, 41.6 and 22.1. IR (neat, NaCl) ν: 3283, 3086, 1733 and 1656 cm^−1^. Mp: 177–179 °C. Analysis: Calculated for C_19_H_16_FN_3_O_5:_ C, 59.22; H, 4.19; N, 10.90. Found: C, 46.93; H, 3.52; N, 9.68.

*N*-(1,4-Dioxide-6-trifluoromethyl-3-phenylquinoxaline-2-carbonyl)glycine methyl ester (**4n**). This compound was obtained in 40% yield as a brown solid following the general procedure. ^1^H NMR (250 MHz, CDCl_3_) δ: 8.76 (t, *J* = 5.3 Hz, 1H), 8.66 (ddd, *J* = 9.5, 6.5, 3.3 Hz, 1H), 7.96 (dd, *J* = 6.5, 3.3 Hz, 1H), 7.81 (d, *J* = 8.3 Hz, 1H), 7.73 (d, *J* = 8.3 Hz, 1H), 4.14 (d, *J* = 5.3 Hz, 1H) and 3.76 (s, 1H). ^13^C NMR (63 MHz, CDCl_3_) δ: 182.0, 169.7, 168.6, 158.9, 138.6, 137.7, 133.2, 130.4, 126.1, 121.2, 53.0 and 41.9. IR (neat, NaCl) ν: 3314, 1738, 1672 and 1334 cm^−1^. Mp: 183–185 °C. Analysis: Calculated for C_19_H_14_F_3_N_3_O_5:_ C, 54.16; H, 3.35; N, 9.97. Found: C, 51.34; H, 3.33; N, 8.57.

#### 4.1.6. General Procedure for the Preparation of N-(1,4-Dioxide-3-Arylquinoxaline-2-Carbonyl)Glycine Derivatives **5**

A mixture of the suitable ester **4** (0.21 mmol), 1,4-dioxane (6 mL) and a solution of 1 M LiOH in water (2 mL) was stirred at 30 °C for 30 min. The mixture was diluted with 10 mL of water and washed with AcOEt (2 × 10 mL); the aqueous layer was neutralized with 1M HCl, extracted with AcOEt (3 × 15 mL) and washed with brine (10 mL). The organic phase was dried and the solvent was evaporated under reduced pressure to obtain the final product.

2-((Carboxymethyl)carbamoyl)-3-(4-fluorophenyl)quinoxaline 1,4-dioxide (**5d**). This compound was obtained in 52% yield as a brown solid following the general procedure. ^1^H NMR (250 MHz, DMSO) δ: 12.76 (s, 1H), 9.04 (t, *J* = 6.1 Hz, 1H), 8.52 (dd, *J* = 6.1 and 3.1 Hz, 2H), 8.04 (dd, *J* = 6.1 and 3.3 Hz, 2H), 7.66 (d, *J* = 6.2 Hz, 1H), 7.63 (d, *J* = 5.5 Hz, 1H), 7.30 (t, *J* = 8.7 Hz, 2H) and 3.82 (d, *J* = 5.2 Hz, 2H). ^13^C NMR (75 MHz, DMSO) δ: 169.9, 158.6, 132.6, 131.3, 119.9, 115.3 and 40.7. IR (neat, NaCl) ν: 3320, 1715, 1650 and 1364 cm^−1^.^.^

2-((Carboxymethyl)carbamoyl)-6-methoxy-3-phenylquinoxaline 1,4-dioxide (**5f**). This compound was obtained in 62% yield as a brown solid following the general procedure. ^1^H NMR (250 MHz, DMSO) δ: 12.77 (s, 1H), 9.06 (t, *J* = 5.2 Hz, 1H), 8.43 (d, *J* = 9.5 Hz, 1H), 7.84 (d, *J* = 2.7 Hz, 1H), 7.64 (dd, *J* = 9.5 and 2.7 Hz, 1H), 7.61–7.54 (m, 2H), 7.51–7.41 (m, 3H), 4.00 (s, 3H) and 3.78 (d, *J* = 5.2 Hz, 3H).

2-((Carboxymethyl)carbamoyl)-6-methoxy-3-(4-(trifluoromethyl)phenyl)quinoxaline 1,4-dioxide (**5h**). This compound was obtained in 65% yield as a brown solid following the general procedure. ^1^H NMR (250 MHz, DMSO) δ: 12.74 (s, 1H), 9.11 (t, *J* = 5.5 Hz, 1H), 8.45 (d, *J* = 9.5 Hz, 1H), 7.89–7.78 (m, 5H), 7.66 (dd, *J* = 9.5, 2.6 Hz, 1H), 4.01 (s, 3H) and 3.82 (d, *J* = 5.6 Hz, 2H). IR (neat, NaCl) ν: 3299, 1707 and 1649 cm^−1^. 

2-((Carboxymethyl)carbamoyl)-3-(4-fluorophenyl)-6-methylquinoxaline 1,4-dioxide (**5m**). This compound was obtained in 58% yield as a brown solid following the general procedure. ^1^H NMR (250 MHz, DMSO) δ: 12.75 (s, 1H), 9.06 (t, *J* = 5.0 Hz, 1H), 8.42 (d, *J* = 9.1 Hz, 1H), 8.34 (s, 1H), 7.88 (d, *J* = 8.6 Hz, 1H), 7.71–7.55 (m, 2H), 7.31 (t, *J* = 8.6 Hz, 2H), 3.83 (d, *J* = 4.8 Hz, 2H) and 2.62 (s, 3H).

2-Carboxy-6-methoxy-3-(4-tolyl)quinoxaline 1,4-dioxide (**6**). A mixture of carboxylate derivative **4j** (0.21 mmol), 1,4-dioxane (6 mL) and a solution of 1 M LiOH in water (2 mL) was stirred at 80 °C for 2 h. The mixture was diluted with 10 mL of water and washed with AcOEt (2 × 10 mL); the aqueous layer was neutralized with 1M HCl, extracted with AcOEt (3 × 15 mL) and washed with brine (10 mL). The organic phase was dried and the solvent was evaporated under reduced pressure to obtain the final product in 58% yield as a brown solid following the general procedure. ^1^H NMR (250 MHz, CDCl_3_) δ: 8.92 (s, 1H), 7.74 (d, *J* = 8.2 Hz, 3H), 7.46 (d, *J* = 2.3 Hz, 2H), 7.28 (d, *J* = 8.0 Hz, 4H), 6.79 (d, *J* = 8.5 Hz, 2H), 6.72 (dd, *J* = 8.6, 2.4 Hz, 2H), 3.81 (s, 3H) and 2.44 (s, 3H). ^13^C-NMR (63 MHz, CDCl_3_) δ: 168.9, 156.0, 153.5, 144.2, 130.7, 130.1, 129.0, 128.8, 122.0, 110.9, 110.0, 100.9, 56.1 and 21.9.

### 4.2. In Vitro Activity against Mycobacterium tuberculosis

#### 4.2.1. First Protocol for Activity Measurement

The test samples were analyzed in vitro against *Mycobacterium tuberculosis* H37Rv (Mtb H37Rv) in a high-throughput screen using an assay adapted from the microdilution AlamarBlue (AB) broth assay as reported by Collins and Franzblau [47]. All manipulations of Mtb H37Rv were conducted in accordance with the Biosafety in Microbiological and Biomedical Laboratories (BMBL) in Biosafety Level 3 containment laboratories. In brief, our assay uses Mtb H37Rv in a 384-well plate format with in-plate DMSO carrier controls, 3.198 µM amikacin, 0.17 µM amikacin controls and 320 compounds. The Mtb H37Rv plus DMSO carrier control provides a 100% growth control for each plate. The 3.198 µM amikacin completely inhibits the growth of the bacteria and is used in place of uninoculated media as the background control; meanwhile, the 0.17 µM amikacin control approximates the MIC of amikacin ranging from 30 to 80% inhibition, indicative of a positive growth inhibition and proper assay performance. The medium used for both compound preparation and Mtb H37Rv plating was assessed for contamination by plating two 384-well plates with the medium alone. The plates were checked for contamination by visual inspection and end-point detection. The compounds were evaluated in a 10-point stacked plate dose–response method. The compounds were serially diluted at 1:2 from 100 µM to 0.195 µM. The plates were read fluorometrically after incubation with the compounds and the addition of AB.

#### 4.2.2. Second Protocol for Activity Measurement

An alternative method for end-point detection was assessed using the Promega reagent BacTiter-Glo™ Microbial Cell Viability (BTG). The BTG plates were briefly incubated for 20 min at room temperature, sealed with Perkin Elmer clear TopSeal A and read from the top using luminescence on a Perkin Elmer Envision instrument (Tres Cantos, Spain).

#### 4.2.3. Autofluorescence

The compounds in media were pre-read from the high concentration plate with no AlamarBlue or bacteria added. Fold increase was calculated using the median of the positive control wells from the AlamarBlue Mtb H37Rv assay for the Mtb H37Rv control wells. The criterion for a compound being considered autofluorescent was defined as having >50% fluorescence of the Mtb H37Rv control wells. None of the analyzed compounds were found to be autofluorescent.

### 4.3. In Vitro Assays for Leishmanicidal Activity

#### 4.3.1. Parasites and Culture Procedure

The following species of Leishmania were used: *L. donovani* (MHOM/IN/80/DD8), which was purchased (ATCC, Manassas, VA, USA), and *L. amazonensis* (MHOM/Br/79/Maria), which was kindly provided by Prof. Alfredo Toraño (Instituto de Salud Carlos III, Madrid, Spain). Promastigotes were cultured in Schneider’s Insect Medium supplemented with 10% heat-inactivated Fetal Bovine Serum (FBS) (SIGMA-Merck, Madrid, Spain) and 1000 U/L of penicillin plus 100 mg/L of streptomycin in 25 mL culture flasks at 26 °C.

#### 4.3.2. J774 Cell Cultivation 

J774 murine macrophages were grown in RPMI-1640 medium (Sigma) supplemented with 10% heat-inactivated FBS (30 min at 56 °C) (SIGMA-Merck), penicillin G (100 U/mL) and streptomycin (100 µg/mL). For the experiments, cells in the pre-confluence phase were harvested with trypsin. Cell cultures were maintained at 37 °C in a humidified environment with 5% CO_2_.

#### 4.3.3. Promastigote Susceptibility Assay

The activity of the compounds on *Leishmania* promastigotes was performed according to the method previously reported by us [52]. Briefly, promastigotes (2.5 × 10^5^ parasites/well) were cultured in 96-well plastic plates. The compounds were dissolved in dimethylsulfoxide (DMSO). Serial dilutions at 1:2 of the test compounds in fresh culture medium were performed (100, 50, 25, 12.5, 6.25, 3.12, 1.56 and 0.78 µg/mL) in a final volume of 200 µL; the solutions were then added to the parasite suspension. After an incubation period of 48 h at 26 °C, a volume of 20 μL of 2.5 mM resazurin solution in PBS was added and the plates were incubated for 3 h under the same conditions. Finally, the Relative Fluorescence Unit (RFU) (535_ex_–590_em_ nm wavelength) was determined in a fluorimeter (Infinite 200 Tecan i-Control). Growth inhibition (%) was calculated by 100—[(RFU treated wells—RFU signal-to-noise)/(RFU untreated—RFU signal-to-noise) × 100]. All tests were carried out in triplicate and the IC50 was calculated by probit analysis using SPSS 17.0 Statistics Software. Miltefosine (Sigma-Merck, Madrid, Spain) was used as the reference drug.

#### 4.3.4. Intracellular Amastigote Assay

We performed an intracellular amastigote assay on *L. amazonensis* and *L. donovani* according to a fluorometric method reported by us [53]. To carry out the in vitro macrophage infections, 5 × 10^4^ cells were cultured in RPMI-1640 medium and then were infected with 5 × 10^5^ promastigotes. In the *L. donovani* assays, the plates were incubated for 48 h at 37 °C, while in the *L. amazonensis* assays, they were incubated at 33 °C for the first 24 h and then the temperature was increased to 37 °C for the next 24 h. After, the culture medium was removed and the cells were washed with buffered RPMI-HEPES at pH 7.4 several times in order to eliminate the non-internalized promastigotes. A total of 100 µL of fresh medium tempered at 37 °C was added to the infected cells, which then were exposed to 100 µL of the test compounds in RPMI-1640 at different concentrations (100, 50, 25, 12.5, 6.25, 3.12, 1.56 and 0.78 µg/mL in a final volume of 200 µL) for 48 h at 37 °C. The culture medium was removed by centrifugation at 3500 rpm for 5 min (Centrifuge 5403 Eppendorf) and a lysis solution (0.02% sodium dodecyl sulfate in RPMI-HEPES) was added. After 20 min, the treated cells were harvested (centrifugation at 3500 rpm for 5 min at 4 °C) and the supernatants were replaced by 200 μL of fresh Schneider’s culture medium. The plates were then incubated at 26 °C for another 3 days to allow for the transformation of viable amastigotes into promastigotes and proliferation. Afterwards, 20 μL/well of 2.5 mM resazurin was added and incubated for another 3 h. Finally, URF was measured and IC50 was estimated as described above. All tests were carried out in triplicate. Miltefosine was used as the reference drug.

#### 4.3.5. Cytotoxicity Assay on Macrophages

Cell viability was evaluated using a modification with resazurin of the colorimetric method described previously [47]. J774 macrophages were seeded (5 × 10^4^ cells/well) in 96-well flat-bottom microplates with 100 µL of RPMI-1640 medium. The cells were allowed to adhere for 4 h at 37 °C in 5% CO_2_, then different concentrations of the test compounds in 100 µL of the medium were added and exposed for 24 h. Growth controls were also included. To evaluate cell viability, 20 µL of a 2.5 mM resazurin solution was added and the plates were returned to the incubator for another 3 h. The reduction in resazurin was determined by measuring the fluorescence intensity (excitation wavelength, 535 nm; emission wavelength, 590 nm). Each concentration was assayed three times. The medium and controls were used in each test as blanks. The cytotoxic effect of the compounds was defined as the 50% reduction in cell viability of treated culture cells with respect to the untreated culture (CC_50_).

#### 4.3.6. Selectivity Index Calculations

The selectivity index (SI) was calculated as SI = CC_50_/IC_50_, as described by Ferreira et al. [54].

## 5. Conclusions

We describe the design of a novel synthetic route and biological evaluation of new QdNOs with amino acid side chains as bifunctional therapeutic agents against tuberculosis and leishmaniasis. The basic structure of quinoxaline 1,4-dioxide was modified with a peptide side chain and aryl side arm at position 2 and 3, respectively. The synthetic route toward these compounds involves a selective diketopiperazine ring opening followed by a modified Beirut reaction. The strategic design of molecules possessing dual properties represents a promising paradigm in drug discovery, effectively tackling the specific challenges associated with infectious diseases and potentially leading to more efficient and less costly treatments. The main conclusions that can be drawn from this SAR study are as follows:

1. The presence of a peptide chain at C-2 improves the antitubercular activity compared to the presence of a carboxylic acid.
2. A higher state of oxidation of the nitrogen atoms of the heterocyclic skeleton increases its activity.
3. The presence of substituents in the benzene rings improves the activity of the compounds.
4. Substituents at the para position of the aromatic side arm are more favorable than their ortho and meta analogues.

The identification of compound **4h**, showing good activity and selectivity both as an antitubercular and antileishmanial agent, establishes a foundation for future investigations for the desired dual profile, with a good balance between the activities found against all of the organisms studied. This compound can be considered an initial candidate for further development.

## Figures and Tables

**Table 1:** Scope and yields obtained in two-step synthesis of compounds **2**.

Entry	R_1_	1, Yield	R_2_	2, Yield
1	H	**1a**, 82%	OMe	**2a**, 71%
2	4-CF_3_	**1b**, 73%	OMe	**2b**, 68%
3	4-F	**1c**, 72%	OMe	**2c**, 45%
4	3-Cl	**1d**, 97%	OMe	**2d**, 98%
5	4-Me	**1e**, 65%	OMe	**2e**, 54%
6	2-Me	**1f**, 50%	OMe	**2f**, 55%
7	3-MeO	**1g**, 57%	OMe	**2g**, 40%
8	2-NO_2_-3,4-(MeO)_2_	**1h**, 80%	OMe	**2h**, 68%
9			OBn	**2i**, 63%

**Table 2:** Scope and yields obtained in synthesis of compounds **4** and **5**.

Entry	R_1_	R_2_	R_3_	4, Yield	5, Yield
1	H	OMe	H	**4a**, 82%	
2	3-MeO	OMe	H	**4b**, 98%	
3	4-CF_3_	OMe	H	**4c**, 78%	
4	4-F	OMe	H	**4d**, 74%	**5d**, 52%
5	2-NO_2_, 3,4-(MeO)_2_	OBn	H	**4e**, 83%	
6	H	OMe	6-OMe	**4f**, 76%	**5f**, 62%
7	3-Cl	OMe	6-OMe	**4g**, 79%	
8	4-CF_3_	OMe	6-OMe	**4h**, 72%	**5h**, 65%
9	4-F	OMe	6-OMe	**4i**, 62%	
10	4-Me	OMe	6-OMe	**4j**, 35%	
11	2-Me	OMe	6-OMe	**4k**, 45%	
12	4-CF_3_	OMe	6-Me	**4l**, 28%	
13	4-F	OMe	6-Me	**4m**, 60%	**5m**, 58%
14	H	OMe	6-CF_3_	**4n**, 40%	

## Data Availability

The data are contained within the article and Supplementary Materials.

## Supplementary Materials

The following supporting information can be downloaded at: https://www.mdpi.com/article/10.3390/ph17040487/s1, numerical biological data and copies of NMR spectra.
