# Supplementary material for: Dual Antitubercular and Antileishmanial Profiles of Quinoxaline Di-N-Oxides Containing an Amino Acidic Side Chain
Source: Pharmaceuticals (Basel). 2024 Apr 11;17(4):487. doi: 10.3390/ph17040487 (PMC11054274; doi:10.3390/ph17040487)
Supplement: Supplementary file 1 [file pharmaceuticals-17-00487-s001.zip › pharmaceuticals-2948363-supplementary.pdf]

Supporting Information

Dual antitubercular and anti-leishmanial profiles of quinoxaline di-*N*-oxides containing an amino acidic side chain

Juan F. González,[a] María-Auxiliadora Dea-Ayuela[b], Lena Huck,[a] José María Orduña,[a] Francisco Bolás-Fernández,[c] Elena de la Cuesta,[a] Nazia Haseen,[d] Ashraf Ali Mohammed,[d] J. Carlos Menéndez[a*]

[a] Unidad de Química Orgánica y Farmacéutica, Departamento de Química en Ciencias Farmacéuticas, Facultad de Farmacia, Universidad Complutense, Plaza de Ramón y Cajal s/n, 28040 Madrid, Spain

[b] Departamento de Farmacia, Facultad de Ciencias de la Salud, Universidad Cardenal Herrera-CEU, CEU Universities, C/ Santiago Ramón y Cajal, Alfara del Patriarca, 46115 Valencia, Spain.

[c] Departamento de Microbiología y Parasitología, Facultad de Farmacia, Universidad Complutense de Madrid, Plaza Ramón y Cajal s/n, 28040 Madrid, Spain.

[d] AMIPRO SDN.BHD. Level 3, Bangunan Inkubator Universiti. Sains@USM, Lebuh Bukit Jambul, 11900 Bayan Lepas, Pulau Pinang, Malaysia.

Correspondence: J. C. Menéndez, josecm@ucm.es

Table of Contents

1. Numerical biological data. S2
2. Copies of ^{1}H and ^{13}C NMR spectra. S5

1. Numerical biological data

Table S1. Antibacterial activity against Mtb H37Rv *

Entry	Compound	IC_{50} (µM)	IC_{90} (µM)	MIC (µM)
1	**4a**	26.30	55.50	100
2	**4b**	40.64	69.83	100
3	**4c**	13.19	25.90	50
4	**4d**	28.57	47.72	50
5	**4e**	25.17	47.86	100
6	**4f**	21.91	47.93	50
7	**4g**	49.95	71.87	100
8	**4h**	8.46	12.42	50
9	**4i**	47.2	66.5	100
10	**4j**	12.87	24.60	50
11	**4k**	25.87	44.60	100
12	**4l**	7.20	15.5	50
13	**4m**	18.28	36.50	50
14	**4n**	11.91	27.93	50
15	**5f**	4.28	9.86	25
16	**5h**	16.38	21.97	50
17	**5m**	17.20	3505	50
18	**6**	44.95	76.38	100
19	**7**	28.28	66.50	100
20	Amikacin	0.07	0.08	0.16
21	Cycloserine	12.47	13.49	25
22	Ethambutol	1.56	32.79	12.5
23	Isoniazid	0.18	0.29	0.31
24	Pyrimethamine	37.35	74.96	100

* None of the compounds showed cytotoxicity in Vero cells at concentrations up to 6.25 µg/mL.

Table S2. Antileishmanial promastigote activity, cytotoxicity and selectivity indexes*

Entry	Compound	*L. amazonensis* IC_{50}, µM (SI)	*L. donovani* IC_{50}, µM (SI)	Toxicity against J774 macrophages CC_{50}, µM
1	**4a**	171.0 ± 8.5 (5.0)	103.4 ± 9.8 (8.2)	849.8
2	**4b**	157.4 ± 7.8 (9.9)	261.1 ± 7.8 (6.0)	1566.6
3	**4c**	> 237.5 (> 30)	> 237.5 (> 30)	6960.1
4	**4d**	124.9 ± 1.43 (4.3)	164.9 ± 10.24 (3.3)	539.1
5	**4e**	25.1 ± 0.56 (0.7)	34.0 ± 5.2 (0.5)	18.5 ± 3.4
6	**4f**	> 261.1 (> 30.0)	> 261.1 (30.0)	7832.9
7	**4g**	111.8 ± 13.7 (64.3)	40.0 ± 6.7 (179.9)	7194.2
8	**4h**	122.0 ± 9.9 (4.5)	25.3 ± 1.55 (21.9)	554.3
9	**4i**	116.9 ± 8.0 (3.2)	46.9 ± 14.2 (12.2)	379.0 ± 49.9
10	**4j**	110.8 ± 4.0 (2.9)	13.8 ± 9.5 (23.2)	318.6 ± 0.84
11	**4k**	117.4 ± 14.3 (2.8)	23.9 ± 2.2 (13.8)	330.0 ± 75.5
12	**4l**	> 229.9 (> 6.0)	229.9 (> 6.0)	1380
13	**4m**	62.8 ± 1.6 (7.2)	84.8 ± 8.9 (5.4)	454.5
14	**4n**	97.3 ± 4.3 (2.0)	43.5 ± 1.1 (4.4)	191.3 ± 29.3
15	**5d**	> 280.1 (> 3.0)	> 280.1 (> 3.0)	840
16	**5f**	> 271.0 (> 30)	> 271.0 (> 30)	8130
17	**5h**	> 228.8 (> 8)	> 228.8 (> 8)	1831
18	**5m**	> 269.5 (> 30)	> 269.5 (> 30)	8086
19	**6**	20.5 ± 2.5 (20.7)	6.2 ± 0.2 (68.4)	424.0
20	Miltefosine	47.9 ± 4.9 (2.8)	0.2 ± 0.02 (680.5)	136.1 ± 10.3

* SI, selectivity index.

Table S3. Anti-amastigote activity, cytotoxicity and selectivity of selected compounds*

Entry	Compound	*L. amazonensis* IC_{50}, μM (SI)	*L. donovani* IC_{50}, μM (SI)	Toxicity against J774 macrophages CC_{50}, μM
1	**4a**	12.6 ± 0.3 (67.3)	ND	850
2	**4d**	9.8 ± 1.0 (54.9)	234.2 ± 5.9 (2.3)	539
3	**4g**	23.2 ± 4.1 (310.6)	77.5 ± 6.2 (92.8)	7194
4	**4h**	20.9 ± 4.0 (26.5)	39.5 ± 7.5 (14.0)	554
5	**4i**	12.9 ± 1.7 (29.9)	71.0 ± 11.0 (5.3)	379
6	**4k**	7.2 ± 0.5 (45.6)	72.2 ± 18.6 (4.6)	330
7	**4m**	74.4 ± 19.3 (6.11)	284.9 ± 114.8 (1.6)	454
8	**6**	31.9 ± 13.2 (13.3)	ND	424
9	Miltefosine	47.6 ± 7.4 (2.9)	7.1 (19.1)	136

* SI, selectivity index.

2. Copies of ^{1}H and ^{13}C NMR Spectra

Compound 1a

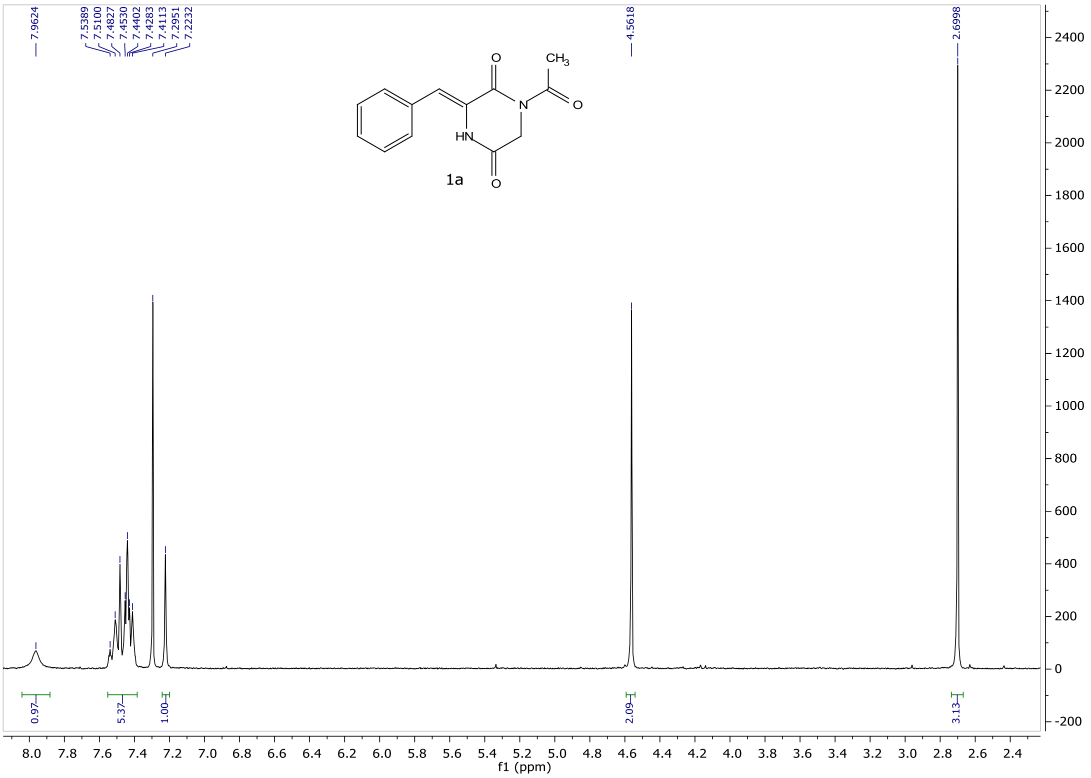

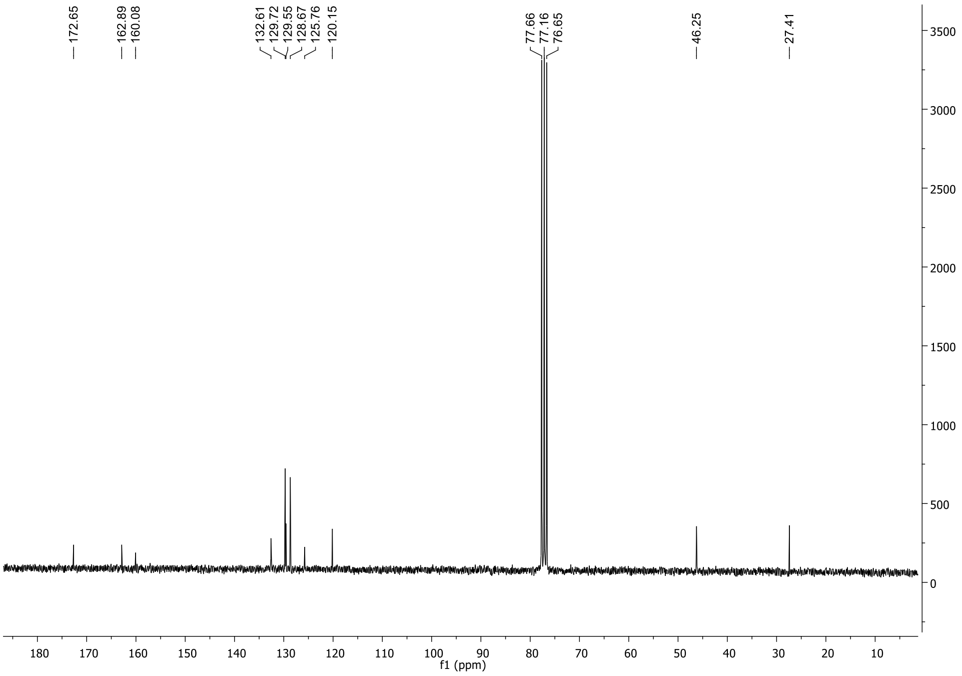

Compound 1b

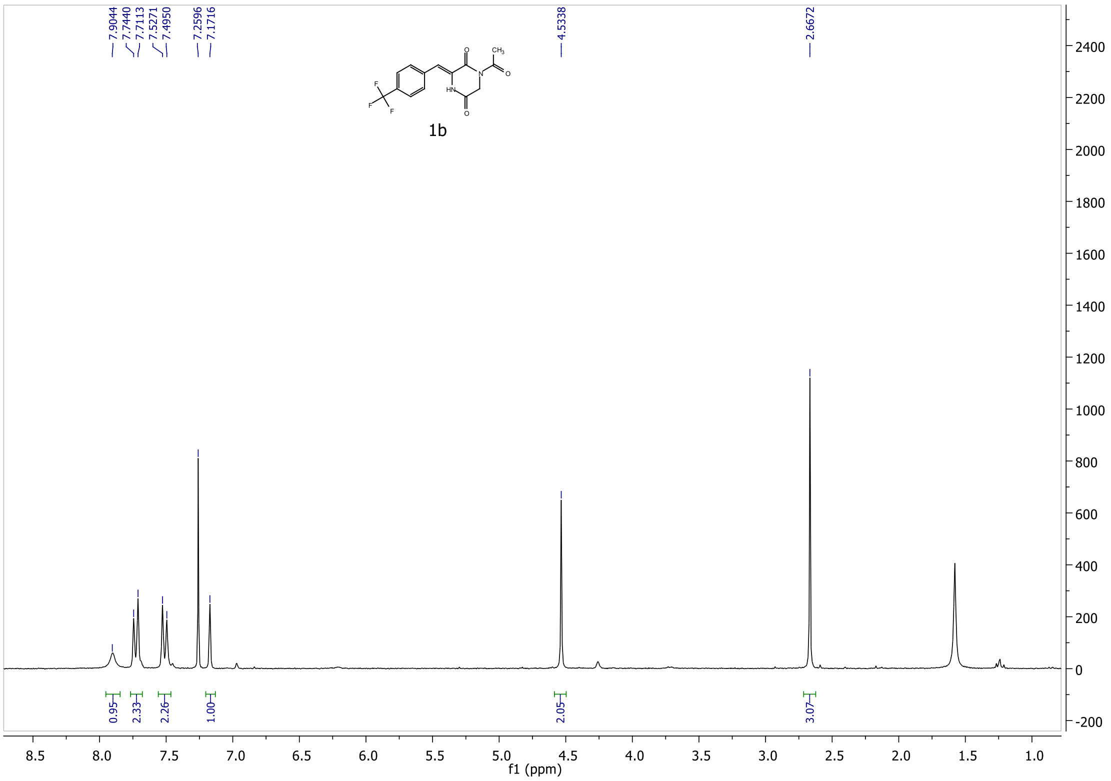
7.9044
7.7440
7.7113
7.5271
7.4950
7.2596
7.1716
4.5338
2.6672
1b
0.95
2.33
2.26
1.00
2.05
3.07
f1 (ppm)

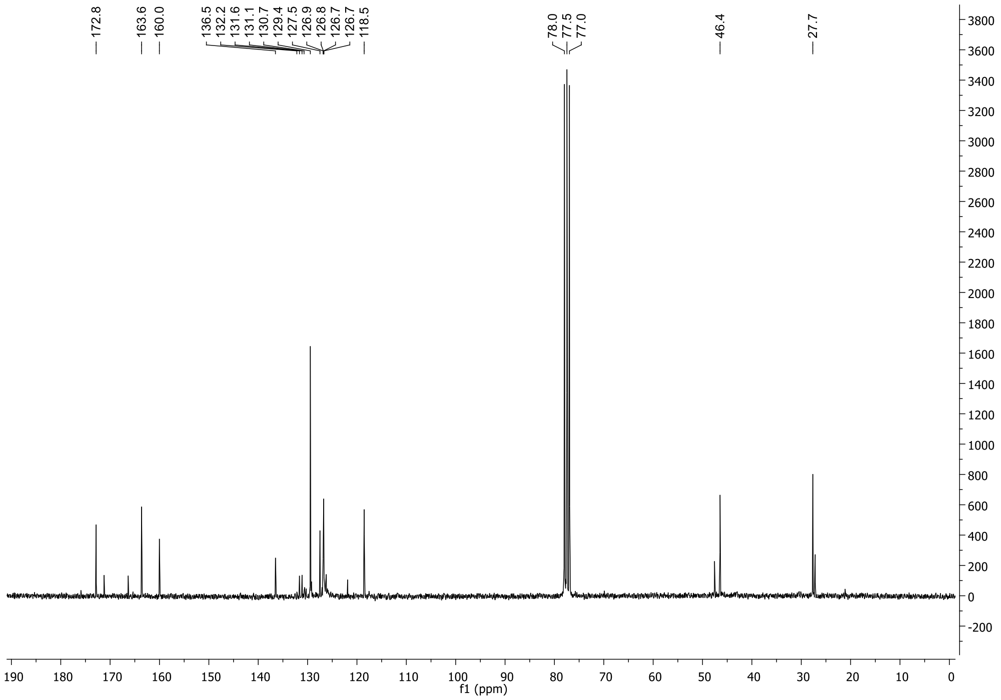
172.8
163.6
160.0
136.5
132.2
131.6
131.1
130.7
129.4
127.5
126.9
126.8
126.7
126.7
118.5
78.0
77.5
77.0
46.4
27.7
f1 (ppm)

Compound 1c

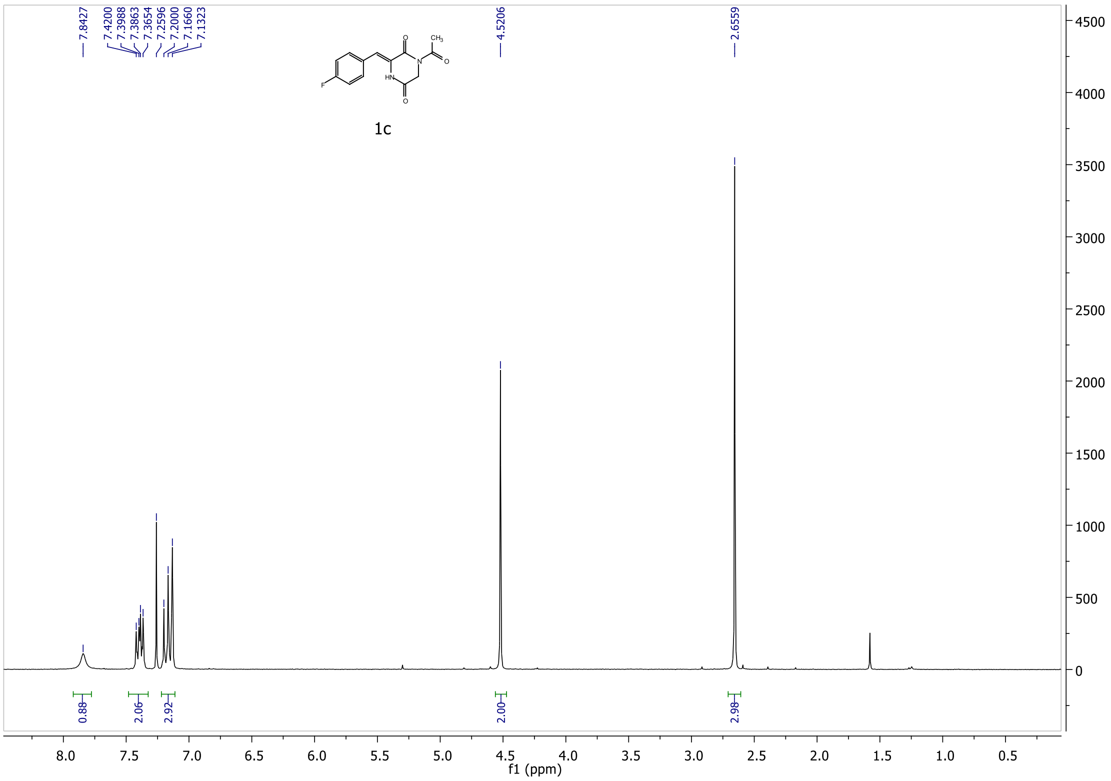

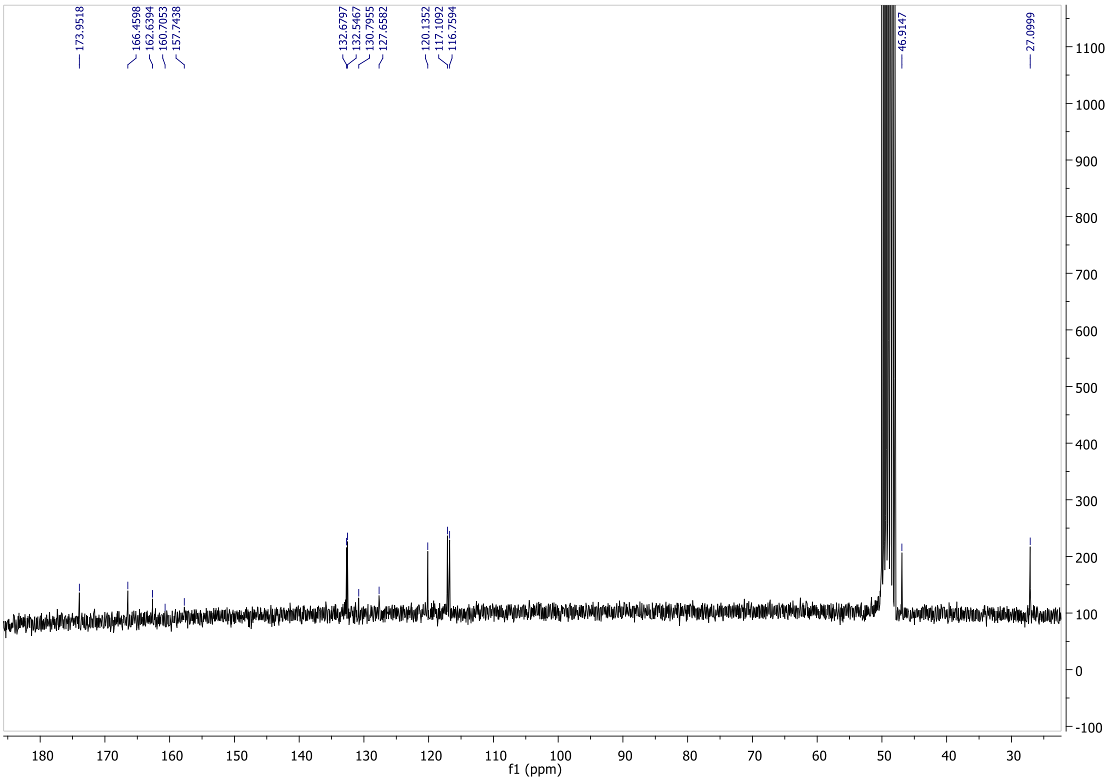

Compound 1d

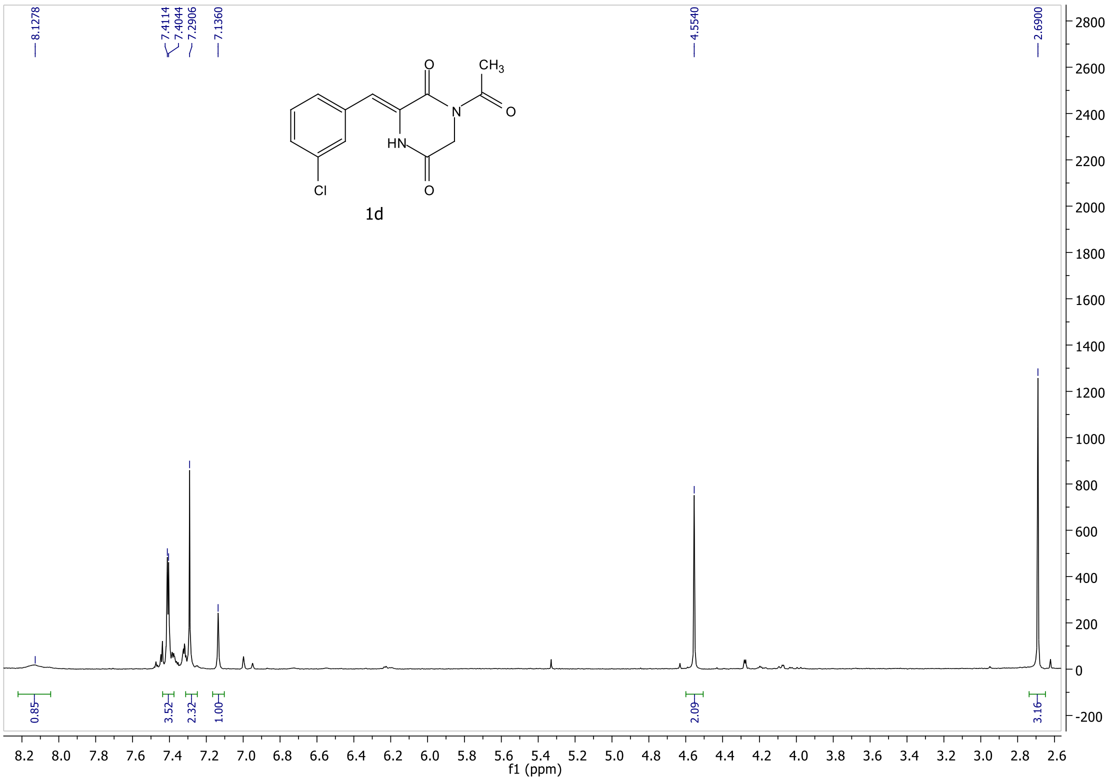

8.1278
7.4114
7.4044
7.2906
7.1360
4.5540
2.6900
1d
0.85
3.52
2.32
1.00
2.09
3.16
f1 (ppm)

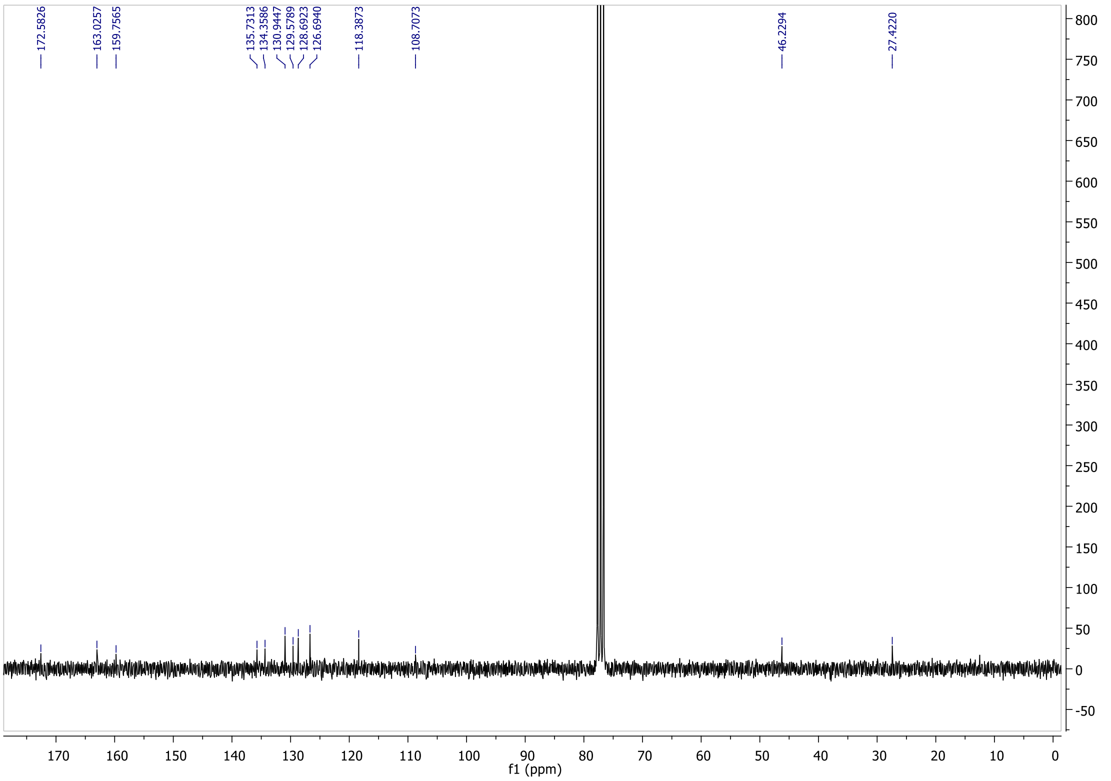

172.5826
163.0257
159.7565
135.7313
134.3586
130.9447
129.5789
128.6923
126.6940
118.3873
108.7073
46.2294
27.4220
f1 (ppm)

Compound 1e

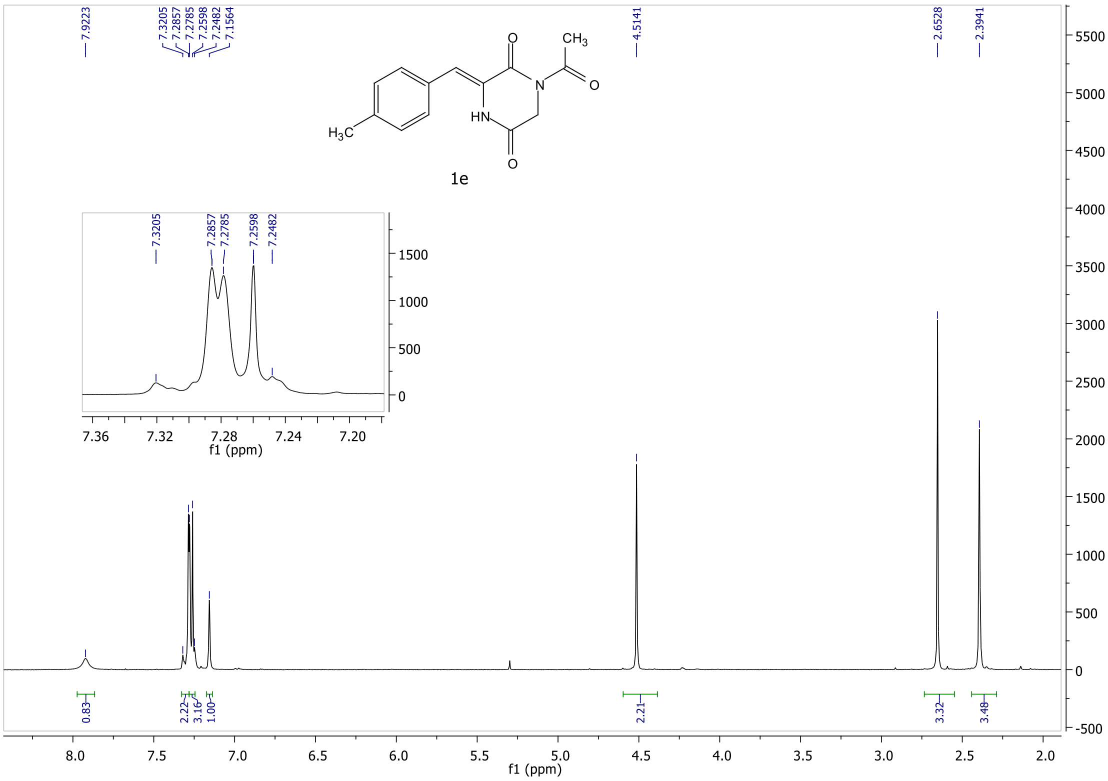
7.9223
7.3205
7.2857
7.2785
7.2598
7.2482
7.1564
4.5141
2.6528
2.3941
CH_3
O
N
HN
H_3C
1e
0.83
2.22
3.16
1.00
2.21
3.32
3.48
f1 (ppm)

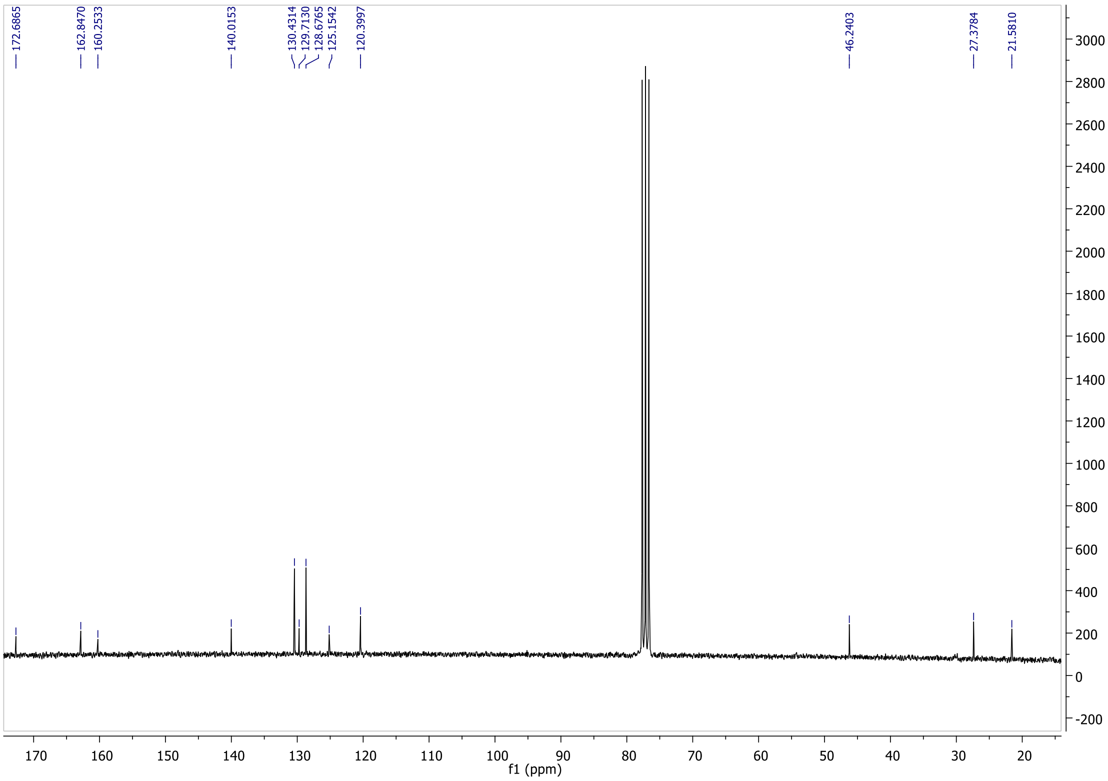
172.6865
162.8470
160.2533
140.0153
130.4314
129.7130
128.6765
125.1542
120.3997
46.2403
27.3784
21.5810
f1 (ppm)

Compound 1f

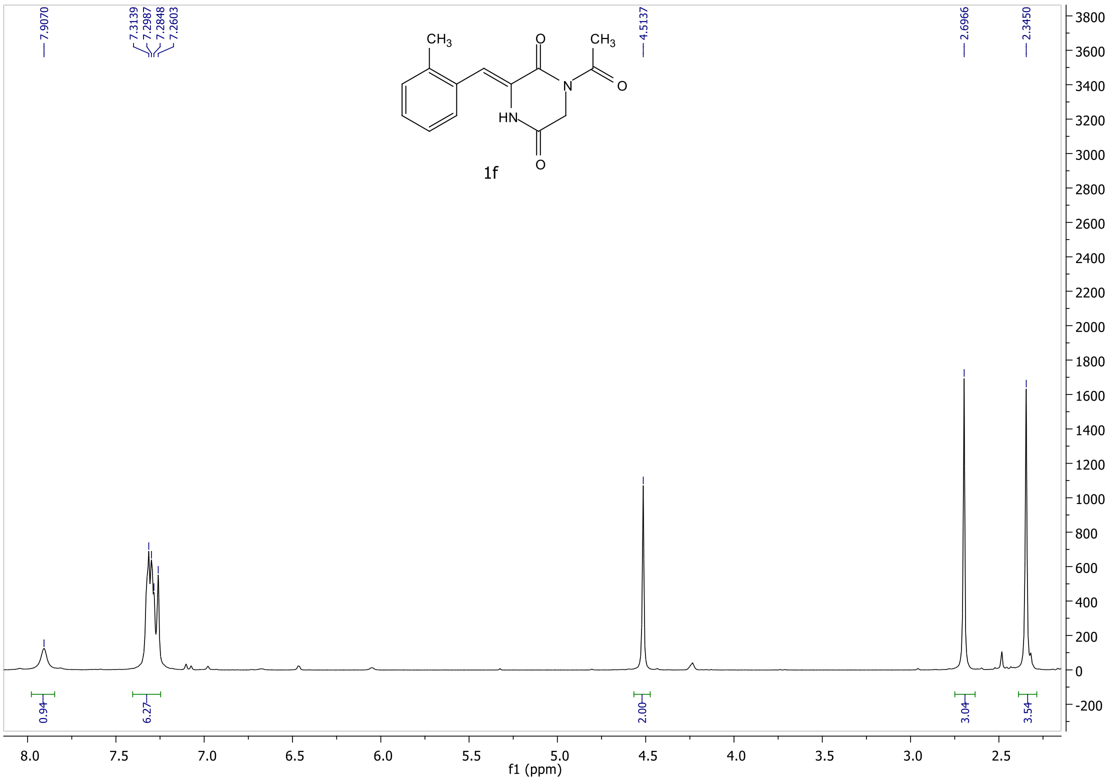
7.9070
7.3139
7.2987
7.2848
7.2603
4.5137
2.6966
2.3450
CH3
O
CH3
N
O
HN
O
1f
0.94
6.27
2.00
3.04
3.54
f1 (ppm)

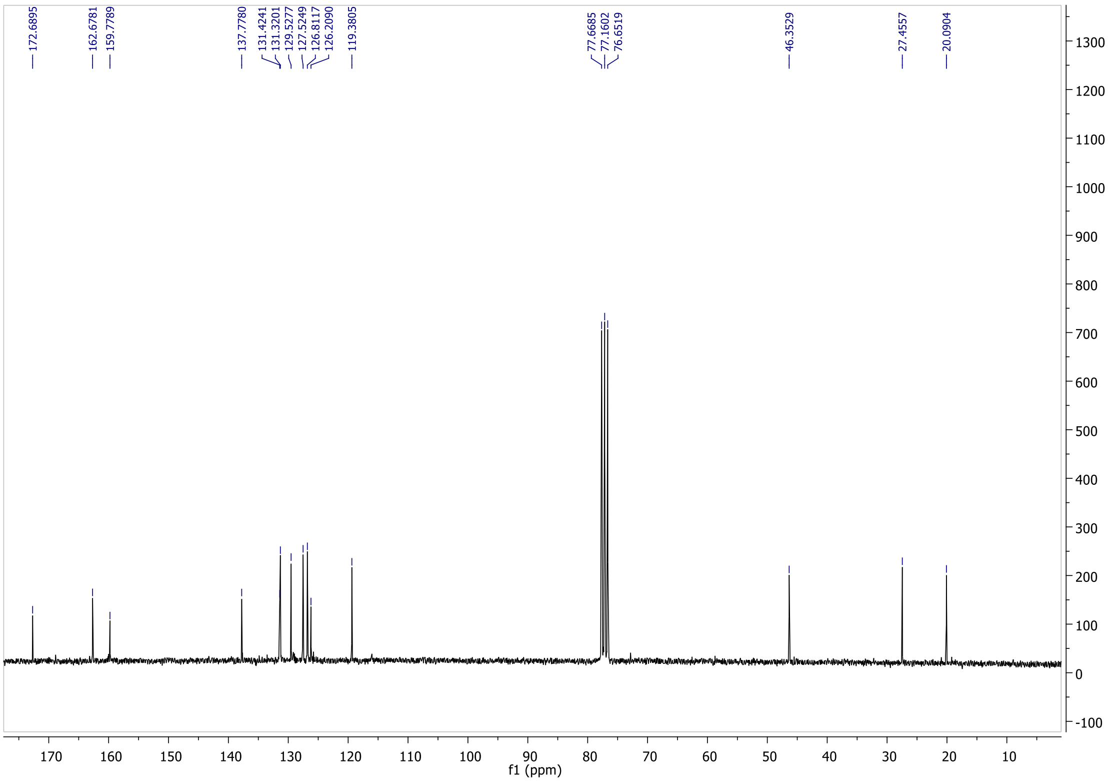
172.6895
162.6781
159.7789
137.7780
131.4241
131.3201
129.5277
127.5249
126.8117
126.2090
119.3805
77.6685
77.1602
76.6519
46.3529
27.4557
20.0904
f1 (ppm)

Compound 1g

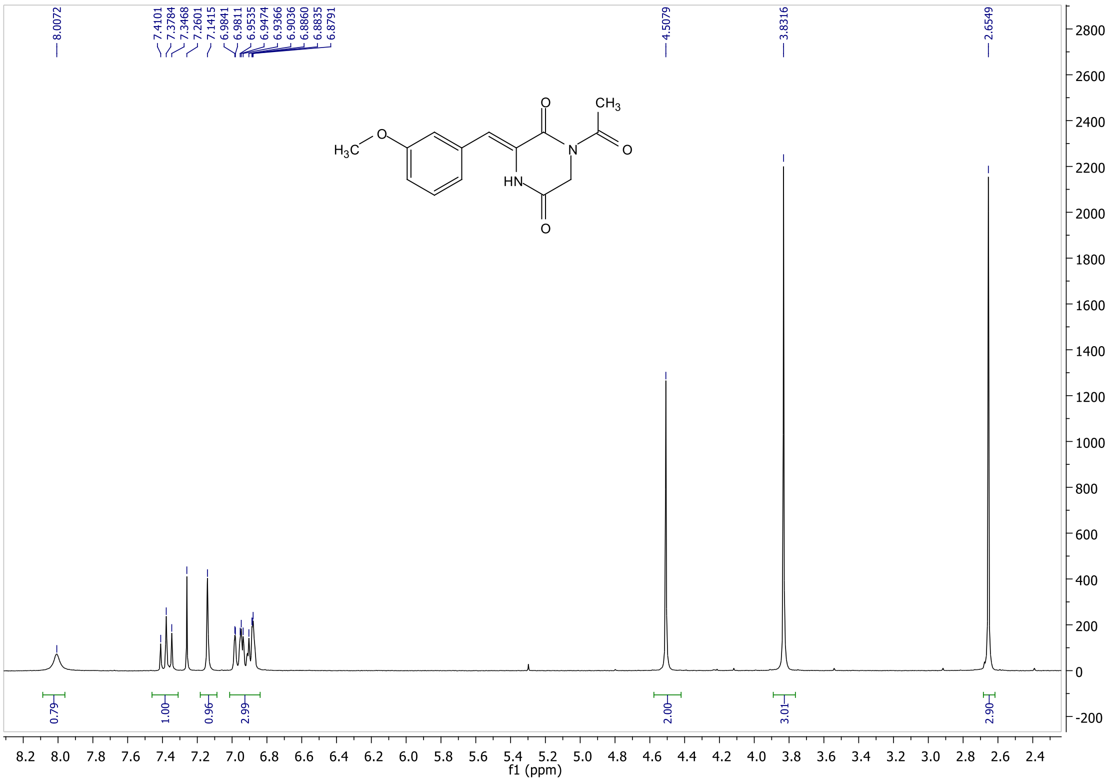

8.0072
7.4101
7.3784
7.3468
7.2601
7.1415
6.9841
6.9811
6.9535
6.9474
6.9366
6.9036
6.8860
6.8835
6.8791
4.5079
3.8316
2.6549
0.79
1.00
0.96
2.99
2.00
3.01
2.90
f1 (ppm)

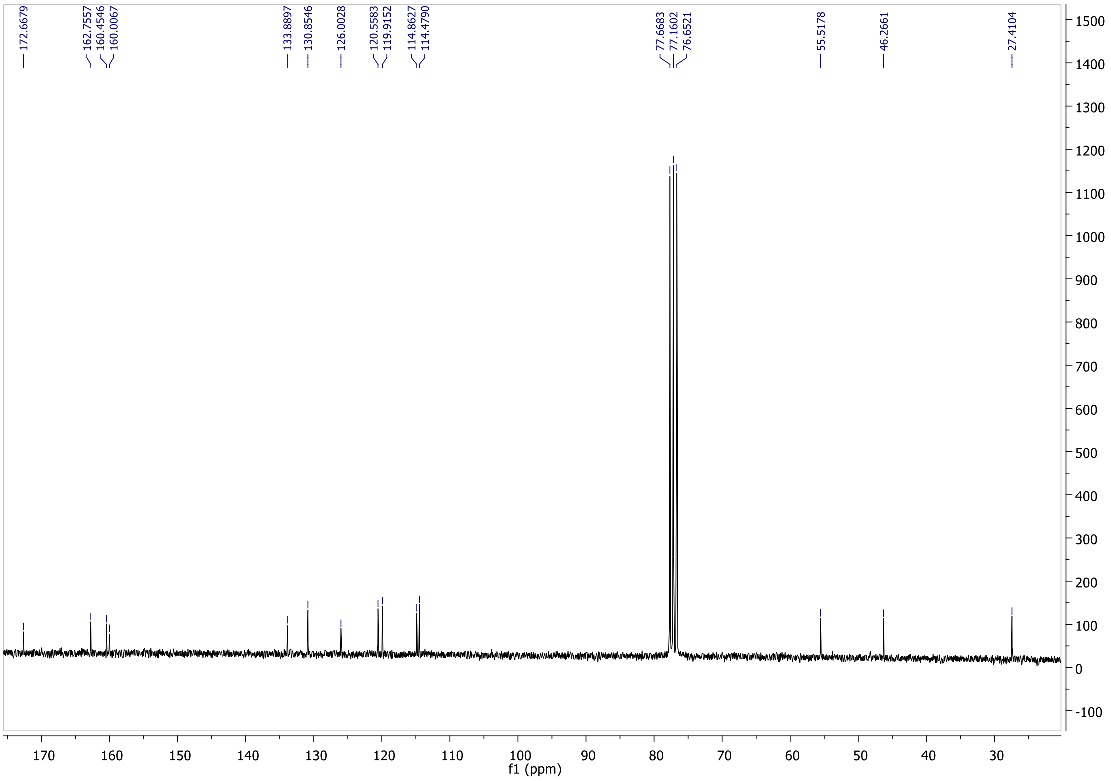

172.6679
162.7557
160.4546
160.0067
133.8897
130.8546
126.0028
120.5583
119.9152
114.8627
114.4790
77.6683
77.1602
76.6521
55.5178
46.2661
27.4104
f1 (ppm)

Compound 1h

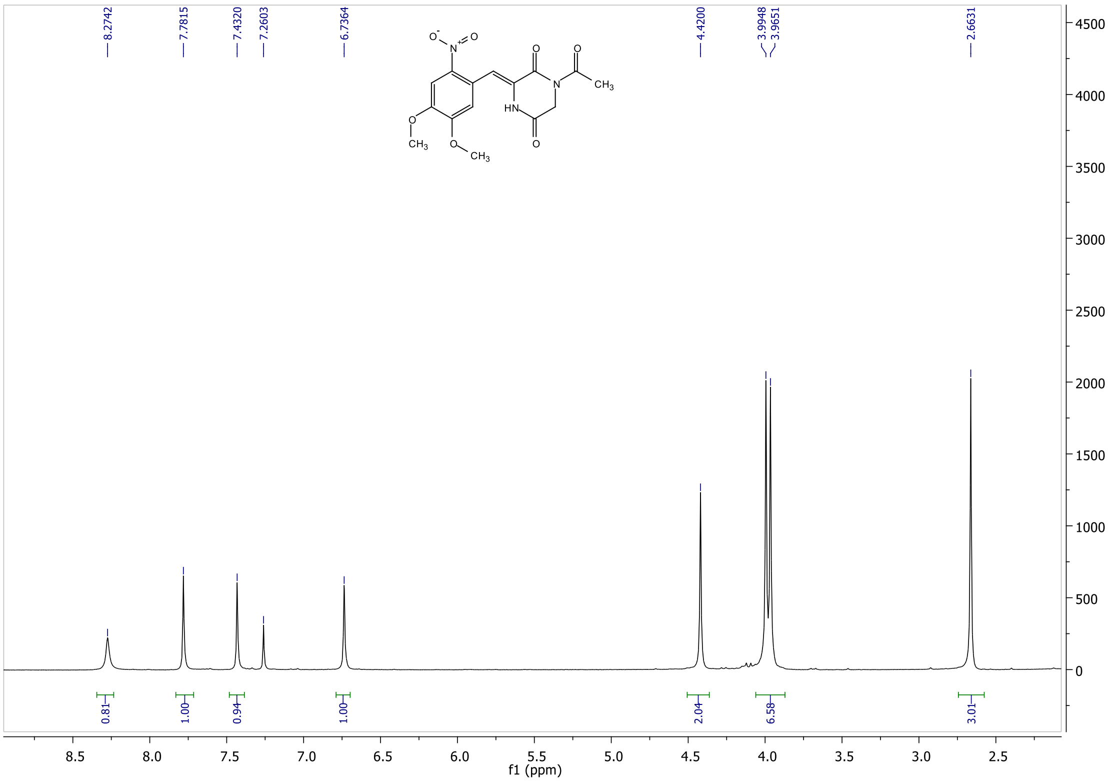

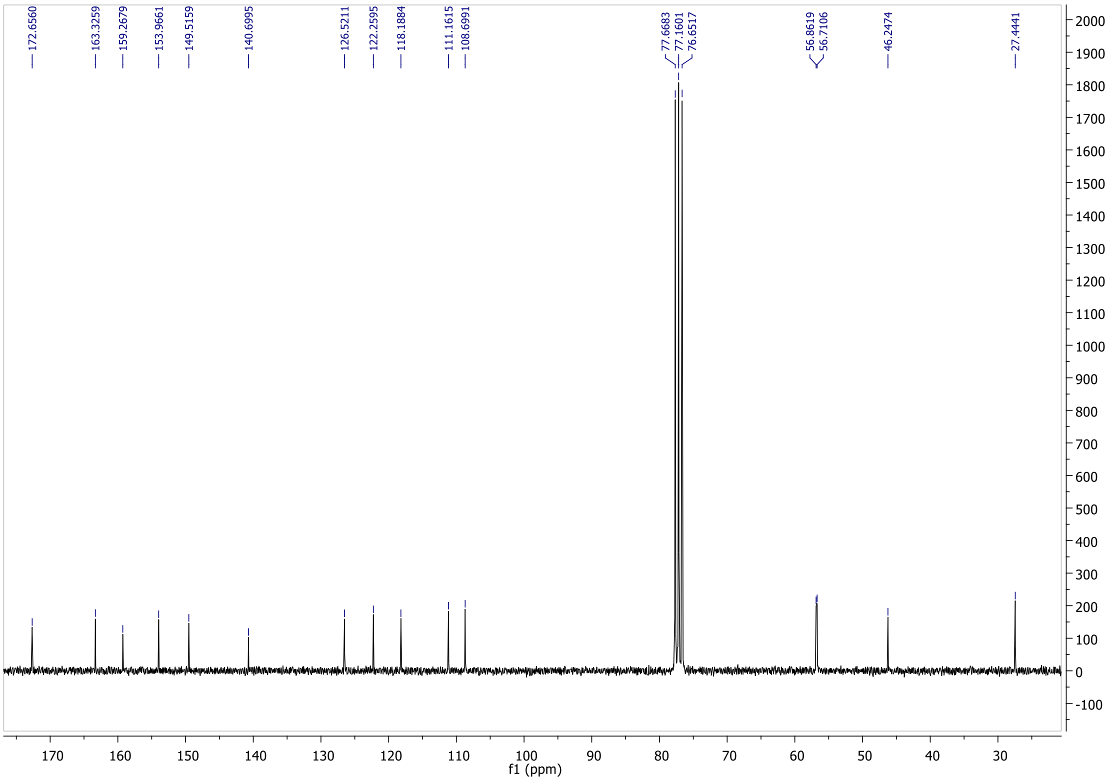

Compound 2a

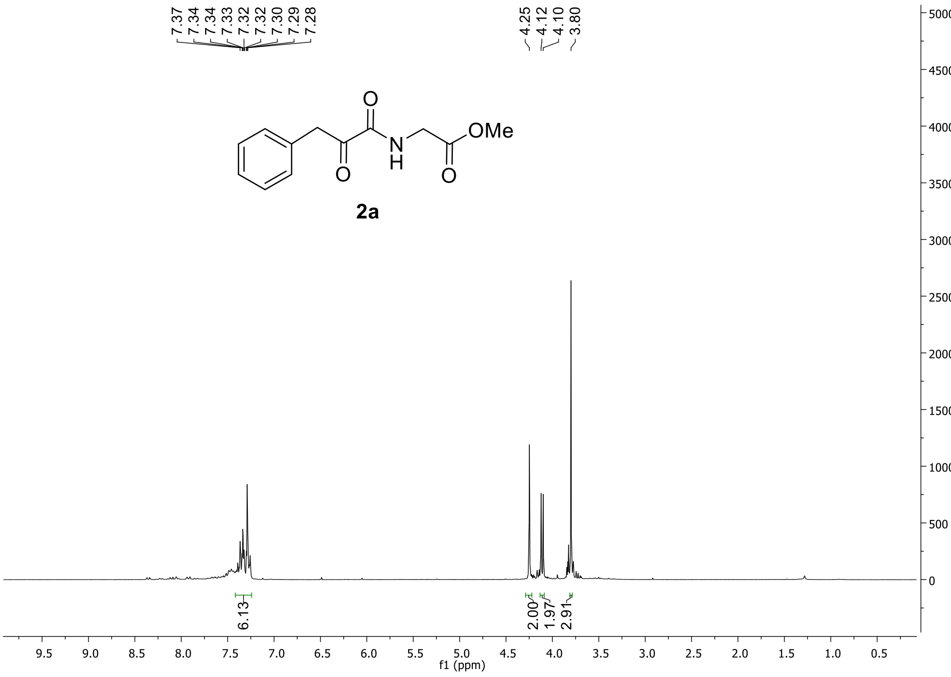

7.37
7.34
7.34
7.33
7.32
7.32
7.30
7.29
7.28
4.25
4.12
4.10
3.80
OMe
2a
6.13
2.00
1.97
2.91
f1 (ppm)

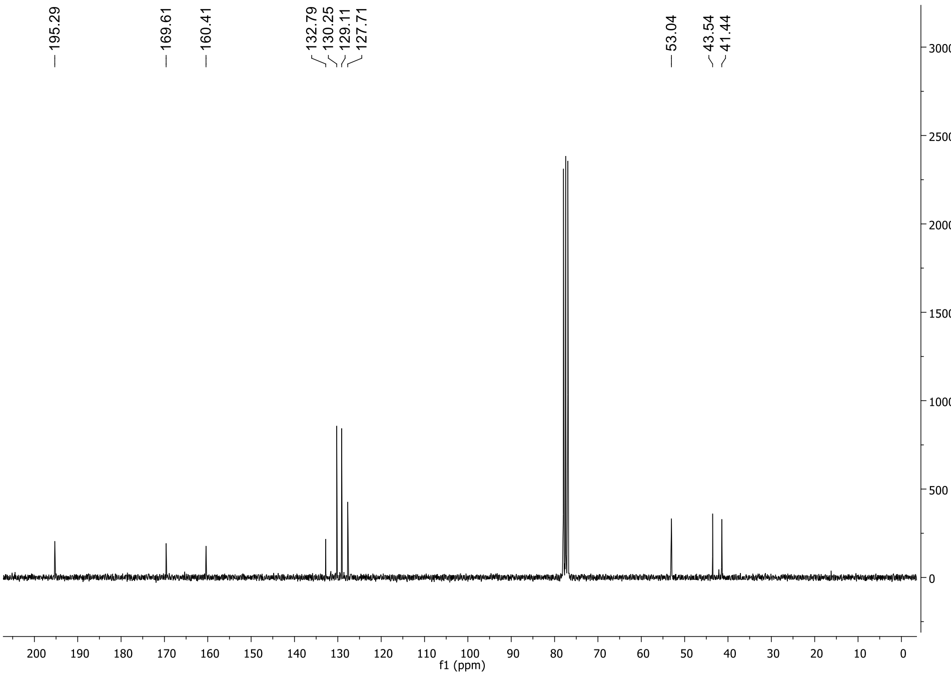

195.29
169.61
160.41
132.79
130.25
129.11
127.71
53.04
43.54
41.44
f1 (ppm)

Compound 2b

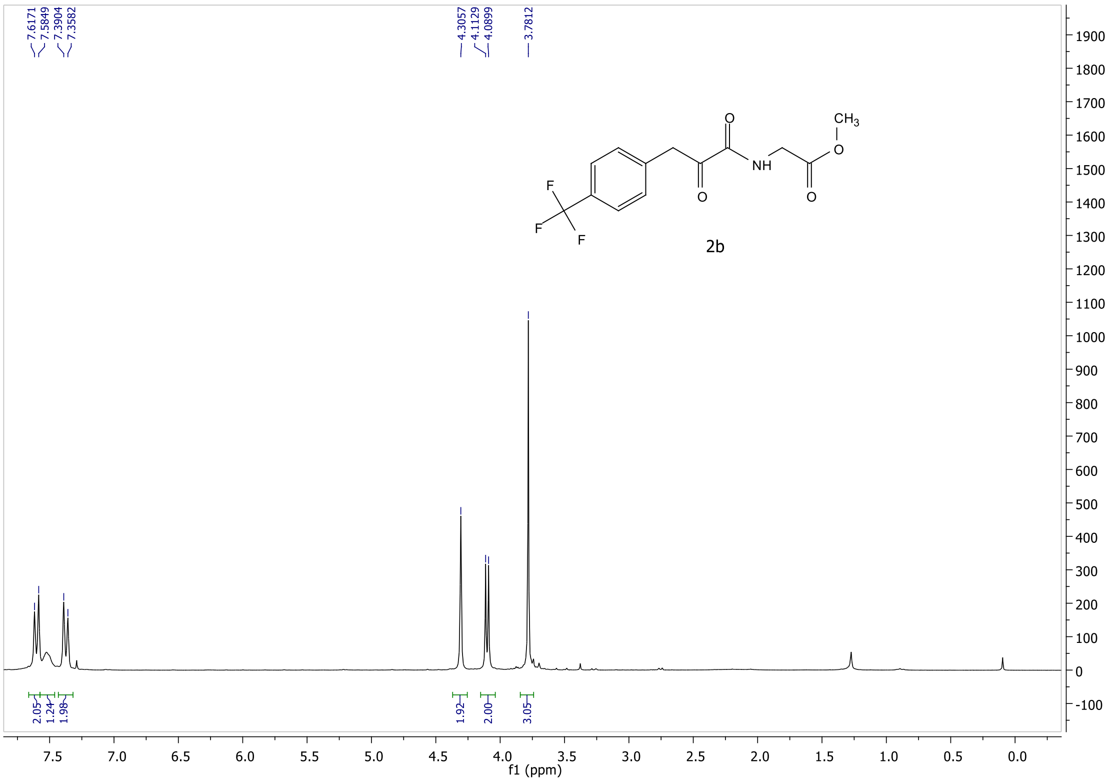

7.6171
7.5849
7.3904
7.3582
4.3057
4.1129
4.0899
3.7812
2b
2.05
1.24
1.98
1.92
2.00
3.05
f1 (ppm)

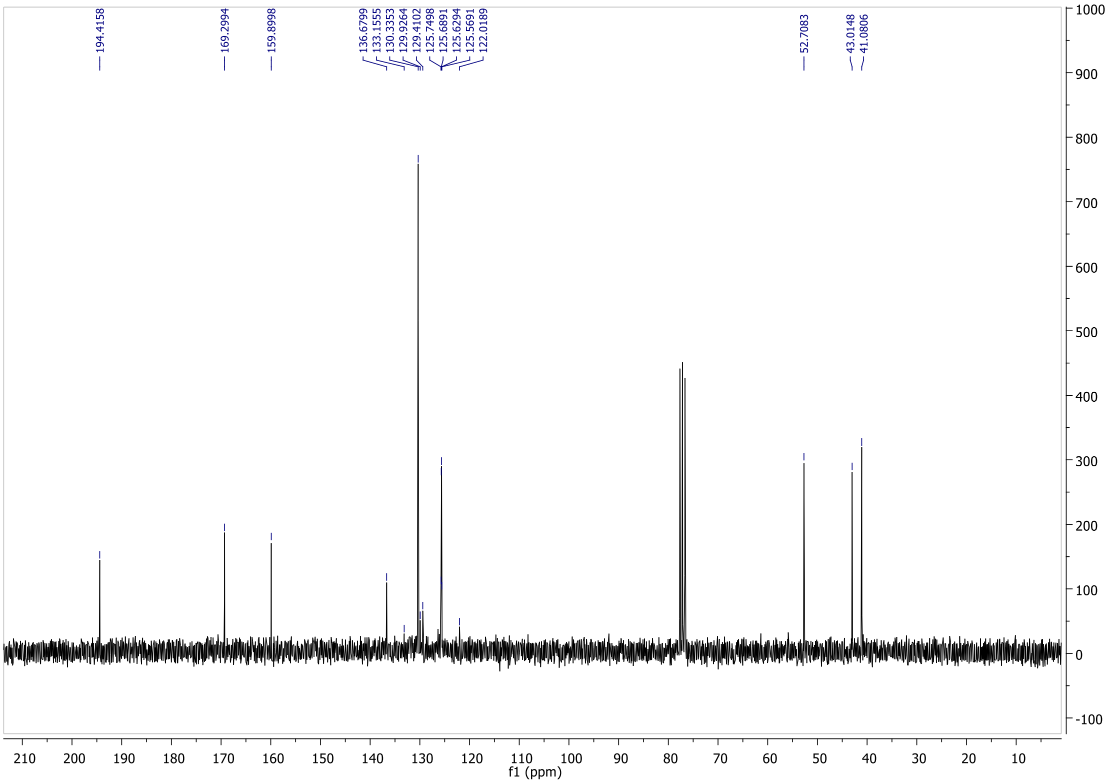

194.4158
169.2994
159.8998
136.6799
133.1555
130.3353
129.9264
129.4102
125.7498
125.6891
125.6294
125.5691
122.0189
52.7083
43.0148
41.0806
f1 (ppm)

Compound 2c

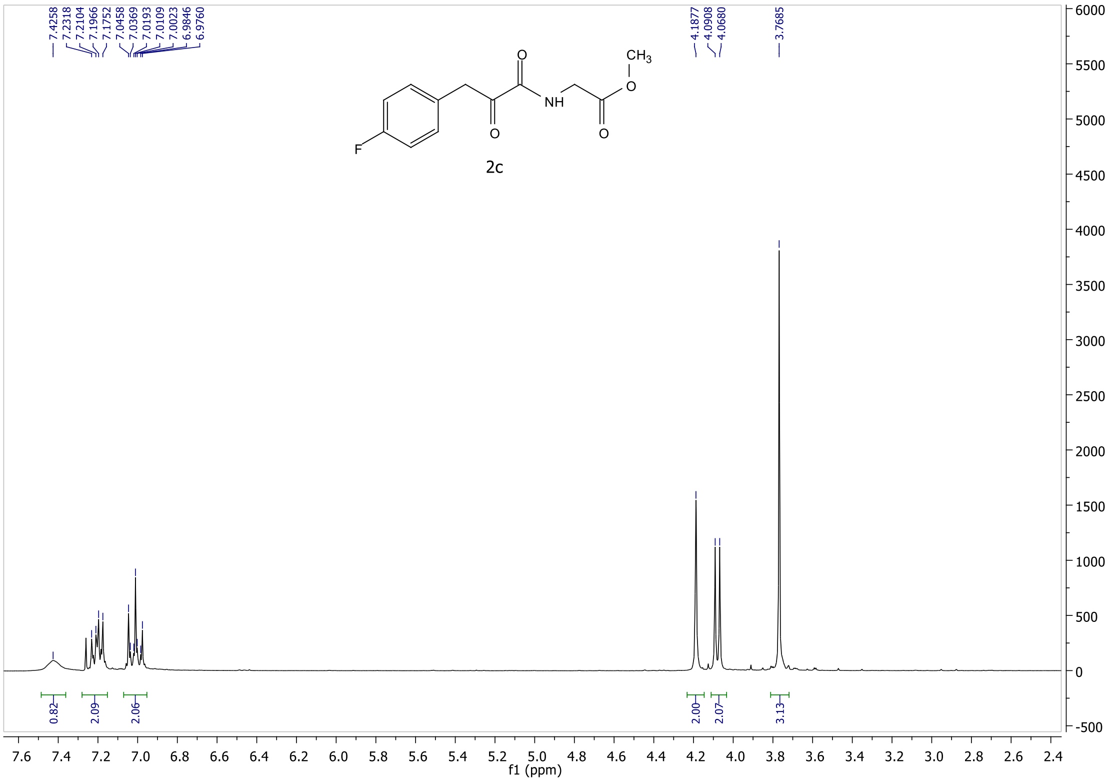

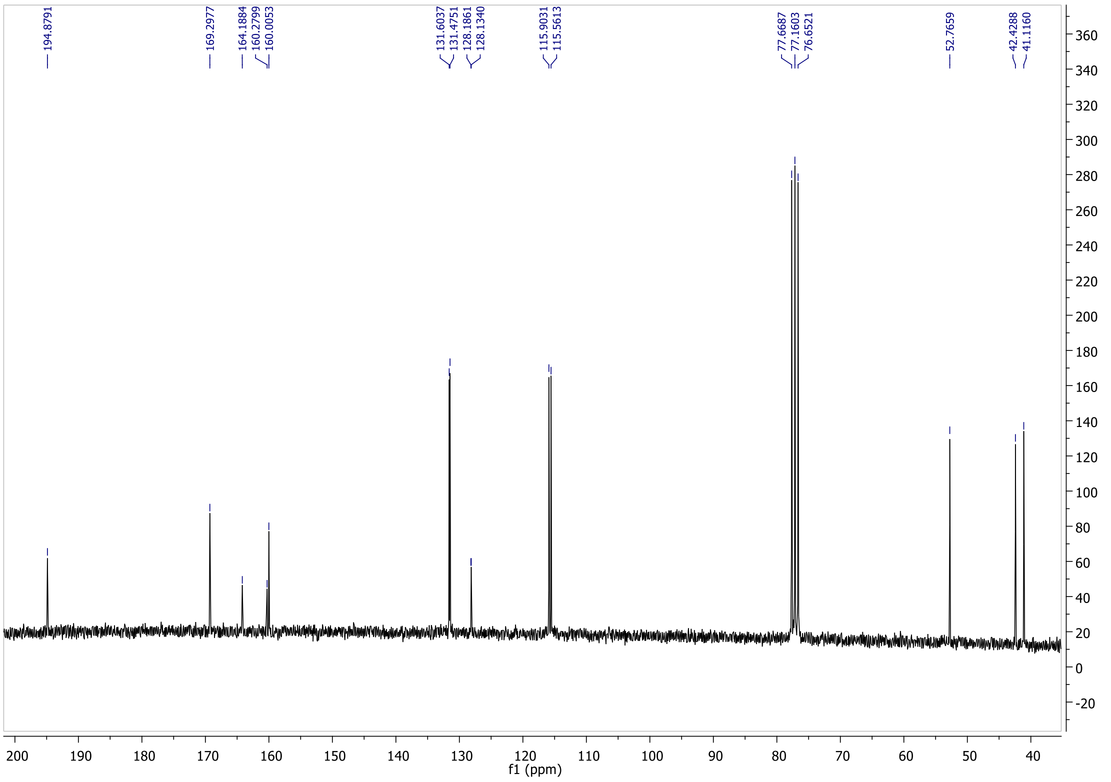

Compound 2d

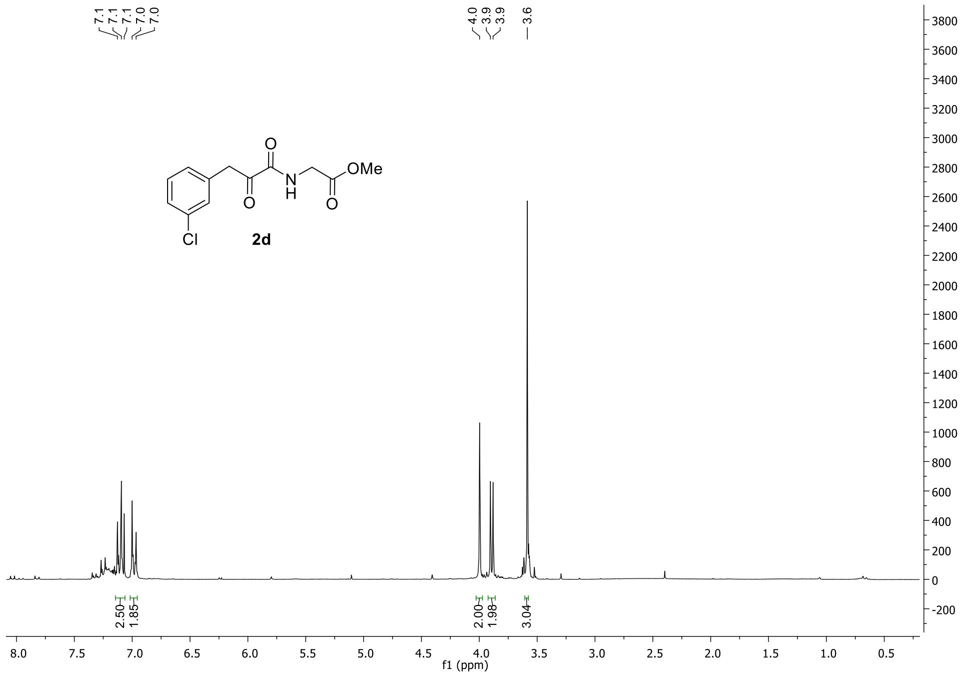
7.1
7.1
7.1
7.0
7.0
4.0
3.9
3.9
3.6
OMe
2d
2.50
1.85
2.00
1.98
3.04
f1 (ppm)

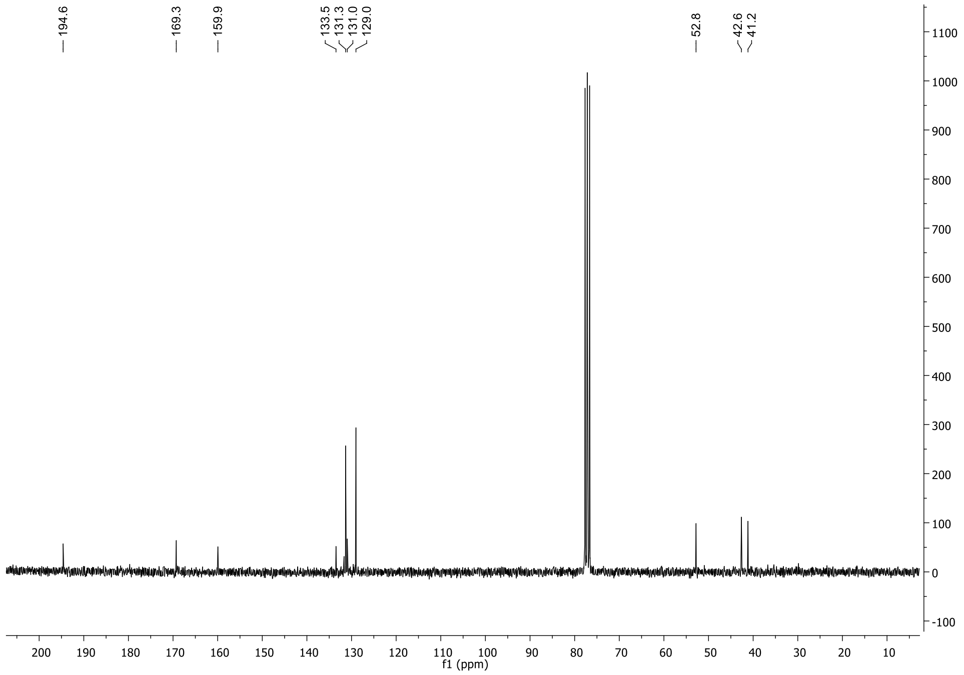
194.6
169.3
159.9
133.5
131.3
131.0
129.0
52.8
42.6
41.2
f1 (ppm)

Compound 2e

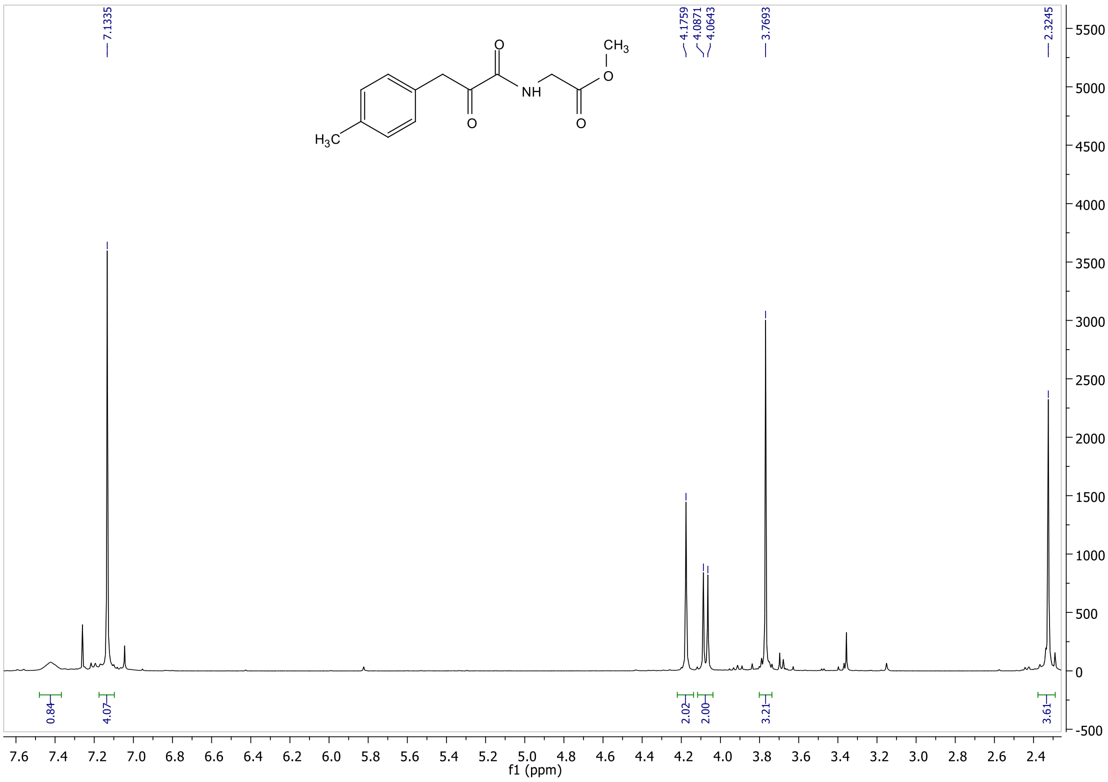

7.1335
4.1759
4.0871
4.0643
3.7693
2.3245
CH3
O
O
NH
O
O
H3C
0.84
4.07
2.02
2.00
3.21
3.61
f1 (ppm)

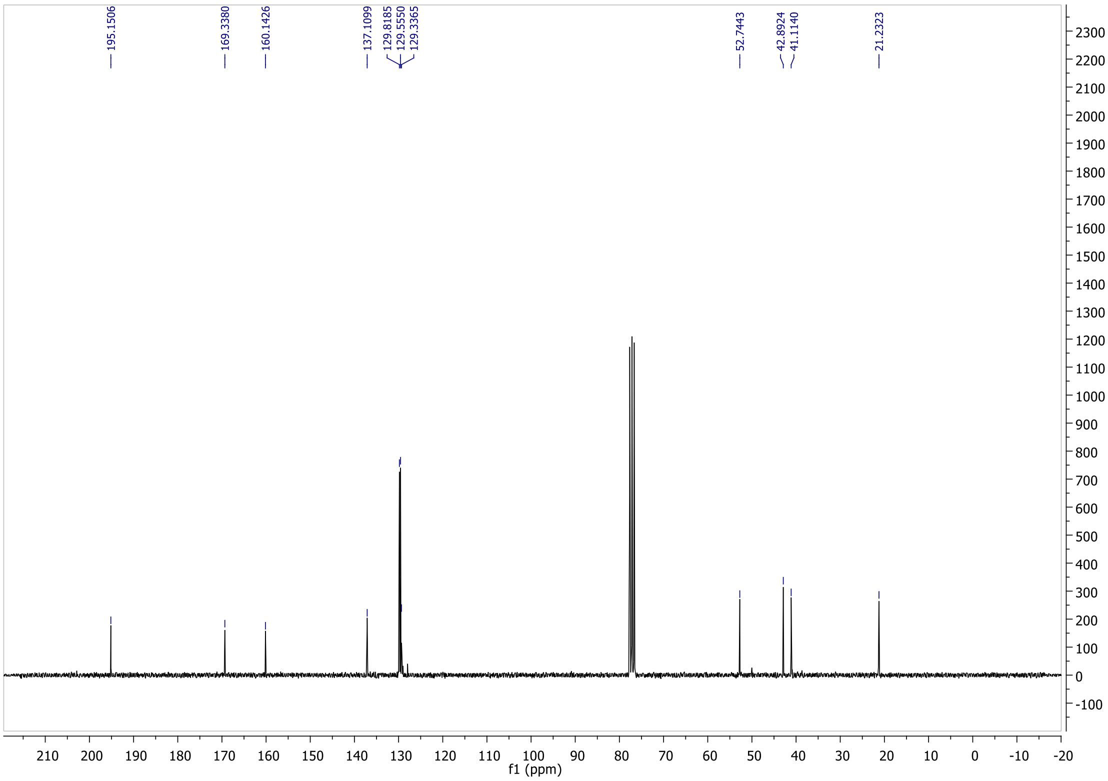

195.1506
169.3380
160.1426
137.1099
129.8185
129.5550
129.3365
52.7443
42.8924
41.1140
21.2323
f1 (ppm)

Compound 2f

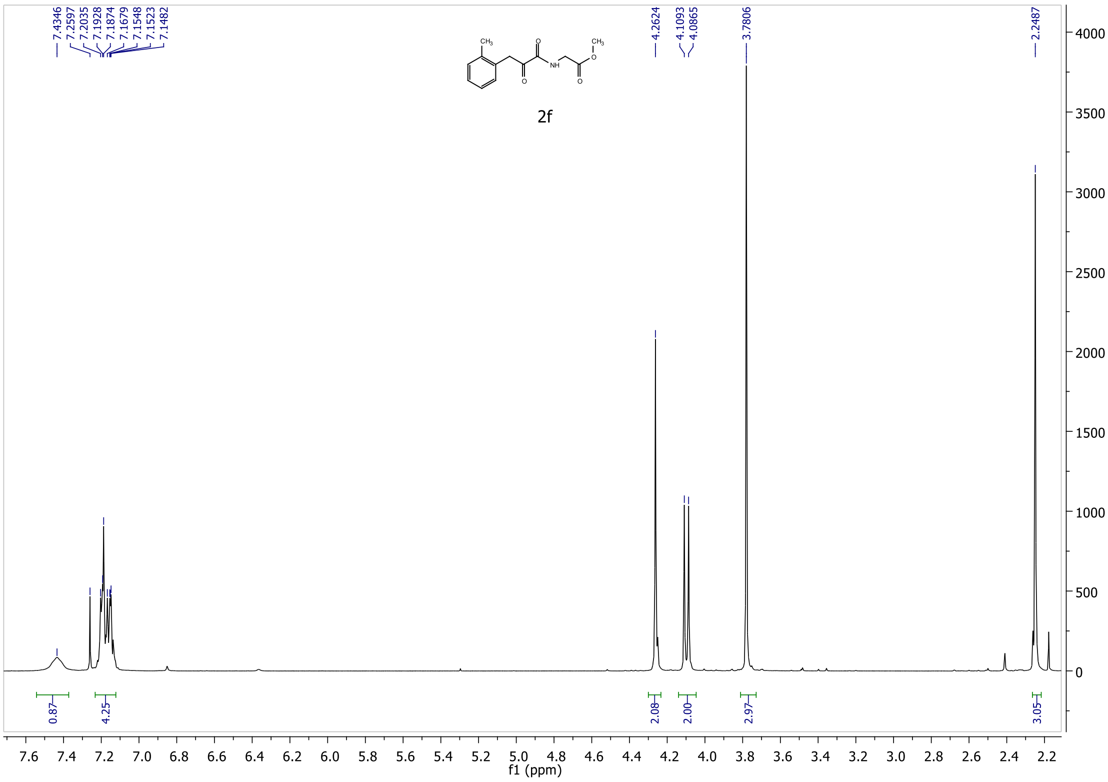

7.4346
7.2597
7.2035
7.1928
7.1874
7.1679
7.1548
7.1523
7.1482
4.2624
4.1093
4.0865
3.7806
2.2487
2f
0.87
4.25
2.08
2.00
2.97
3.05
f1 (ppm)

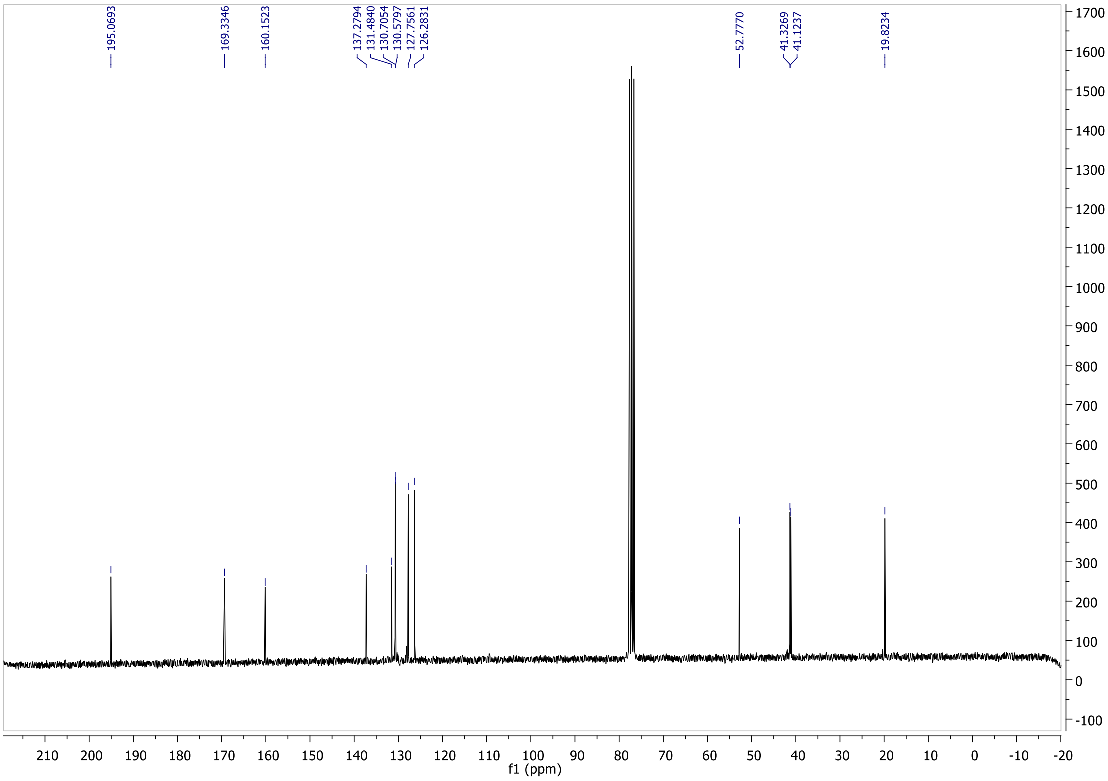

195.0693
169.3346
160.1523
137.2794
131.4840
130.7054
130.5797
127.7561
126.2831
52.7770
41.3269
41.1237
19.8234
f1 (ppm)

Compound 2g

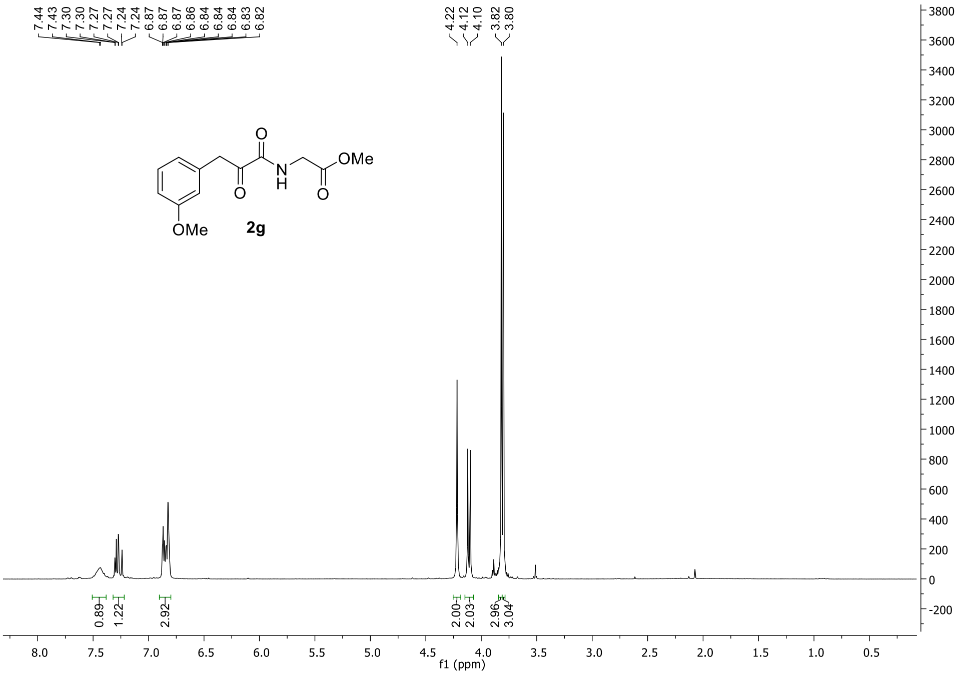

7.44
7.43
7.30
7.27
7.27
7.24
7.24
6.87
6.87
6.87
6.86
6.84
6.84
6.84
6.83
6.82
4.22
4.12
4.10
3.82
3.80
OMe
OMe
N
H
2g
0.89
1.22
2.92
2.00
2.03
2.96
3.04
f1 (ppm)

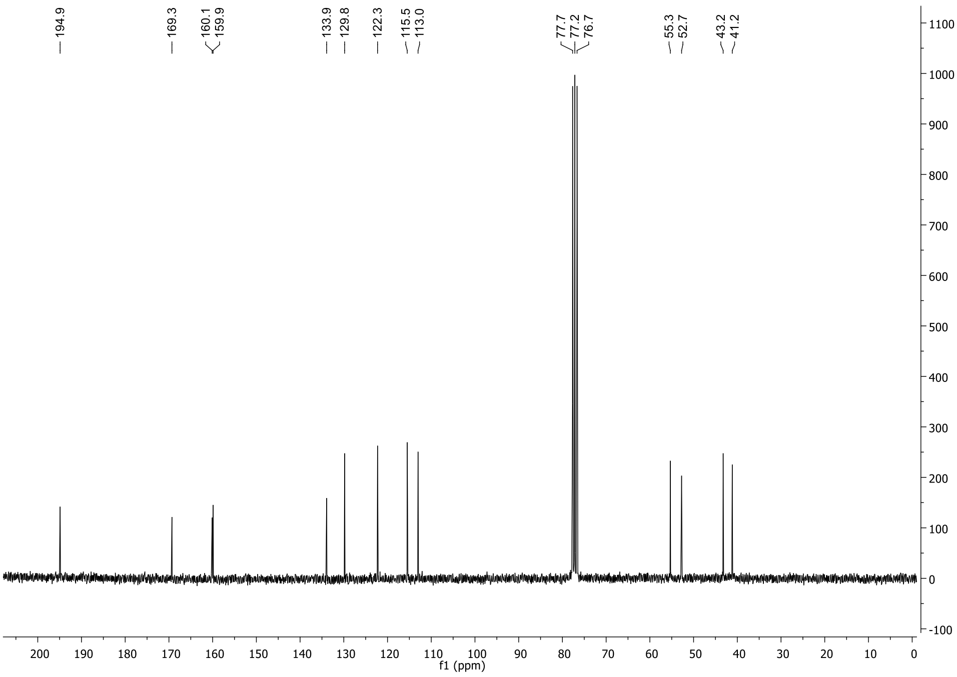

194.9
169.3
160.1
159.9
133.9
129.8
122.3
115.5
113.0
77.7
77.2
76.7
55.3
52.7
43.2
41.2
f1 (ppm)

Compound 2h

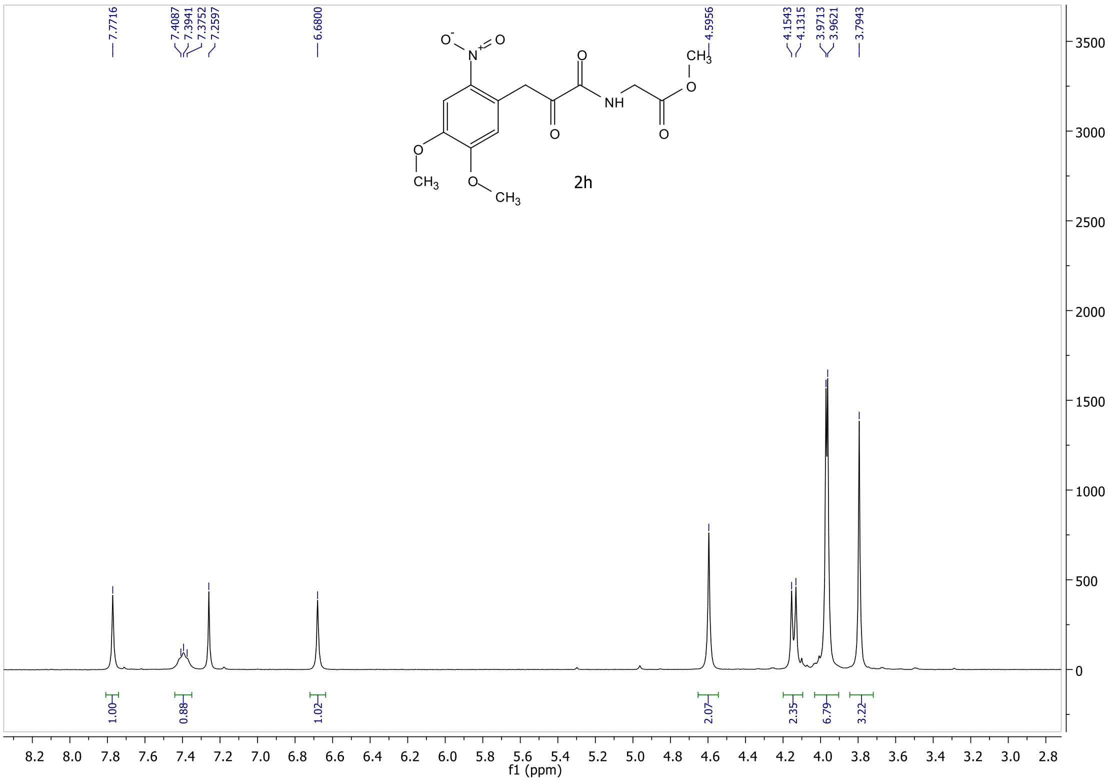

7.7716
7.4087
7.3941
7.3752
7.2597
6.6800
4.5956
4.1543
4.1315
3.9713
3.9621
3.7943
2h
1.00
0.88
1.02
2.07
2.35
6.79
3.22
f1 (ppm)

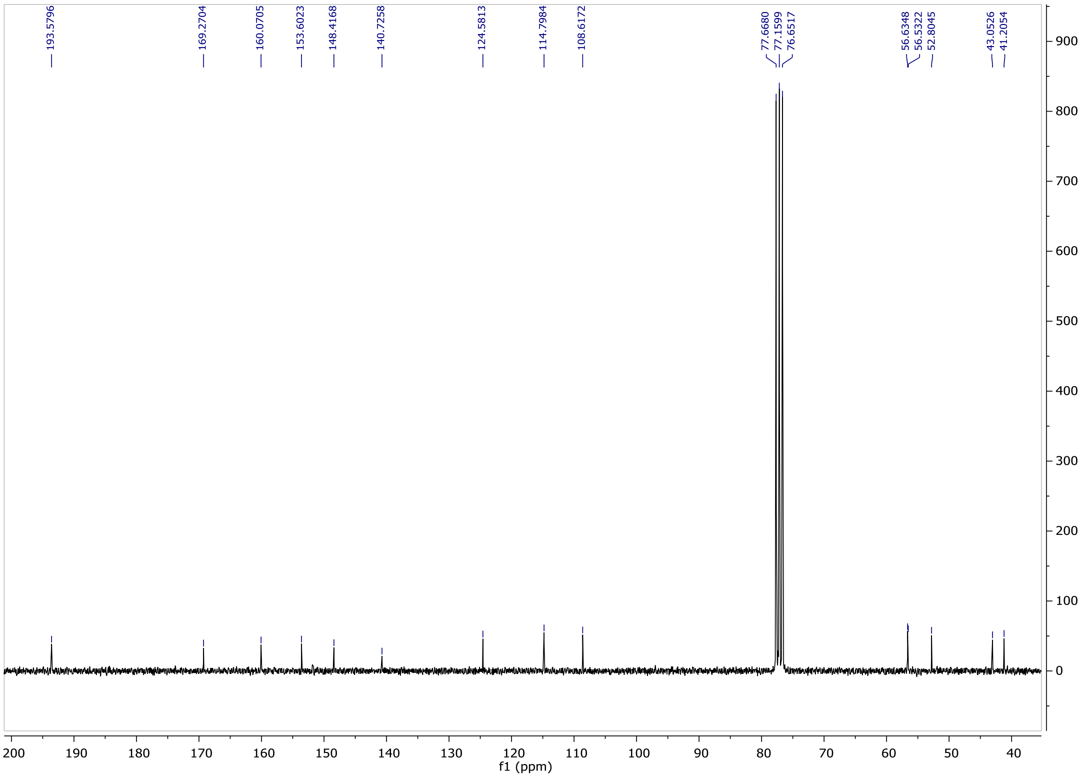

193.5796
169.2704
160.0705
153.6023
148.4168
140.7258
124.5813
114.7984
108.6172
77.6680
77.1599
76.6517
56.6348
56.5322
52.8045
43.0526
41.2054
f1 (ppm)

Compound 2i

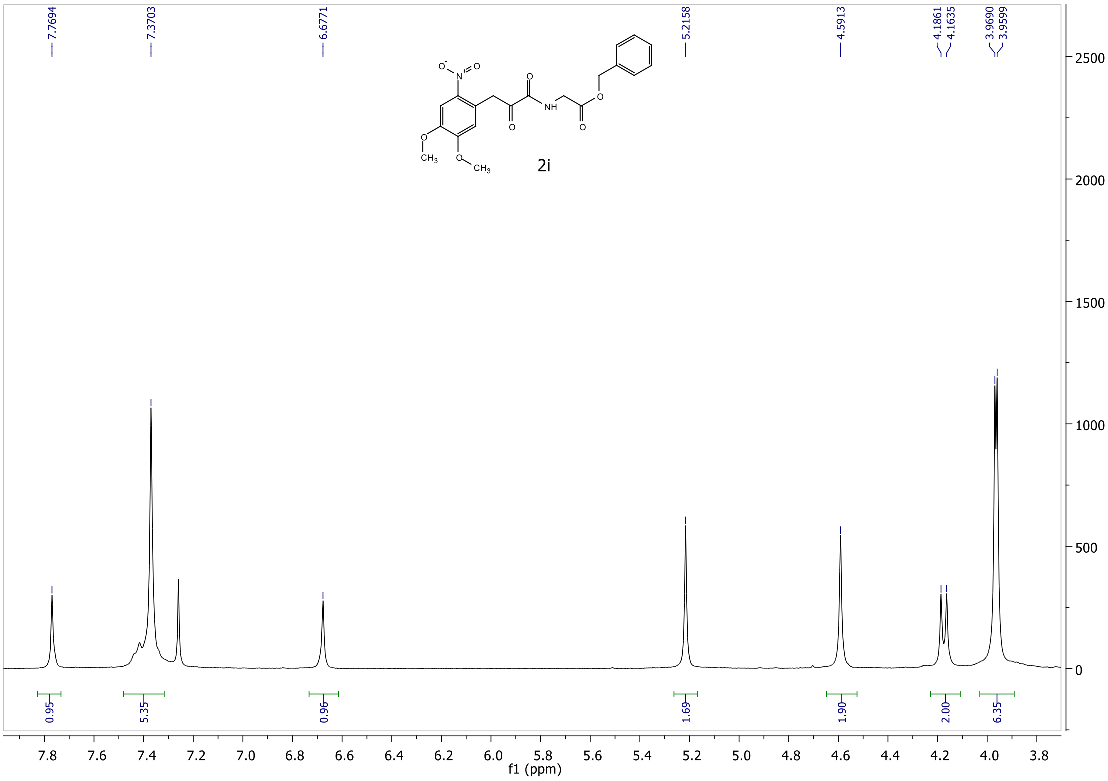
7.7694
7.3703
6.6771
5.2158
4.5913
4.1861
4.1635
3.9690
3.9599
2i
0.95
5.35
0.96
1.69
1.90
2.00
6.35
f1 (ppm)

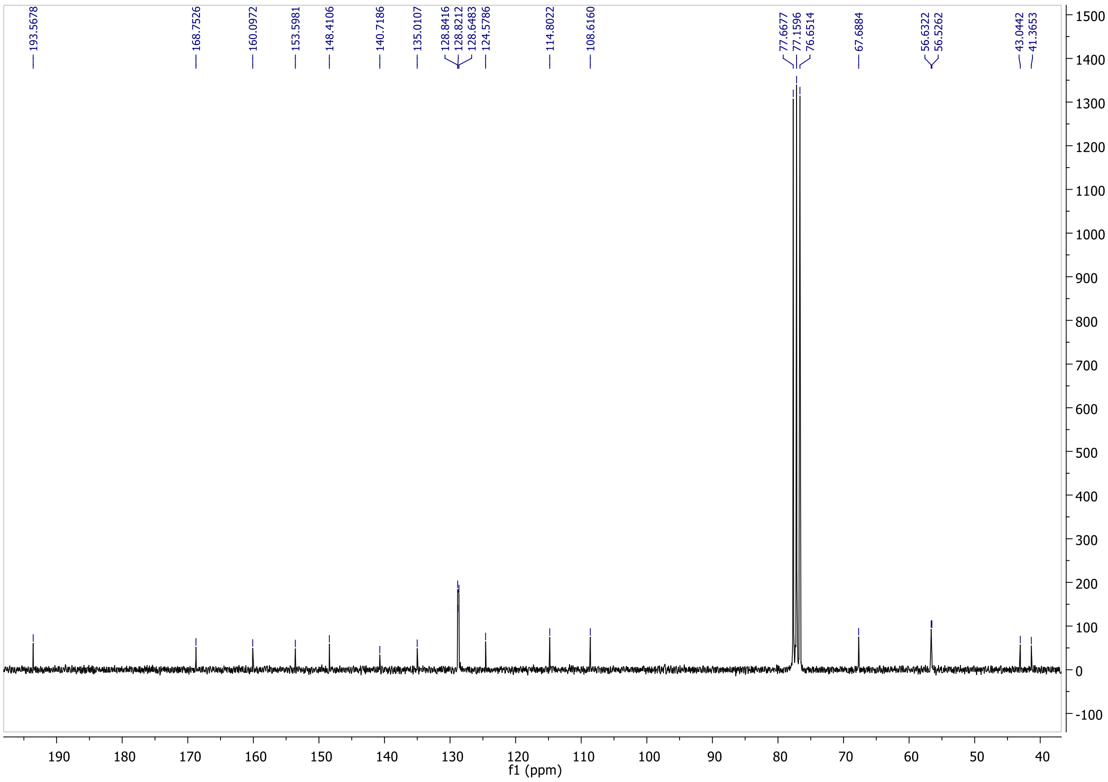
193.5678
168.7526
160.0972
153.5981
148.4106
140.7186
135.0107
128.8416
128.8212
128.6483
124.5786
114.8022
108.6160
77.6677
77.1596
76.6514
67.6884
56.6322
56.5262
43.0442
41.3653
f1 (ppm)

Compound 4a

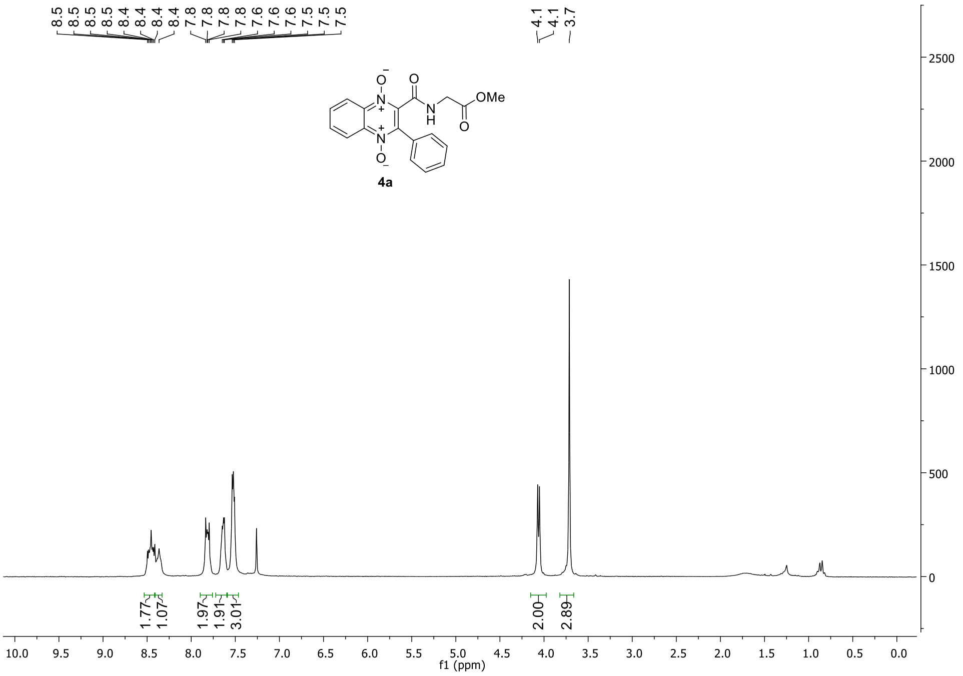
8.5
8.5
8.5
8.5
8.4
8.4
8.4
8.4
7.8
7.8
7.8
7.8
7.6
7.6
7.6
7.5
7.5
7.5
4.1
4.1
3.7
OMe
4a
1.77
1.07
1.97
1.91
3.01
2.00
2.89
f1 (ppm)

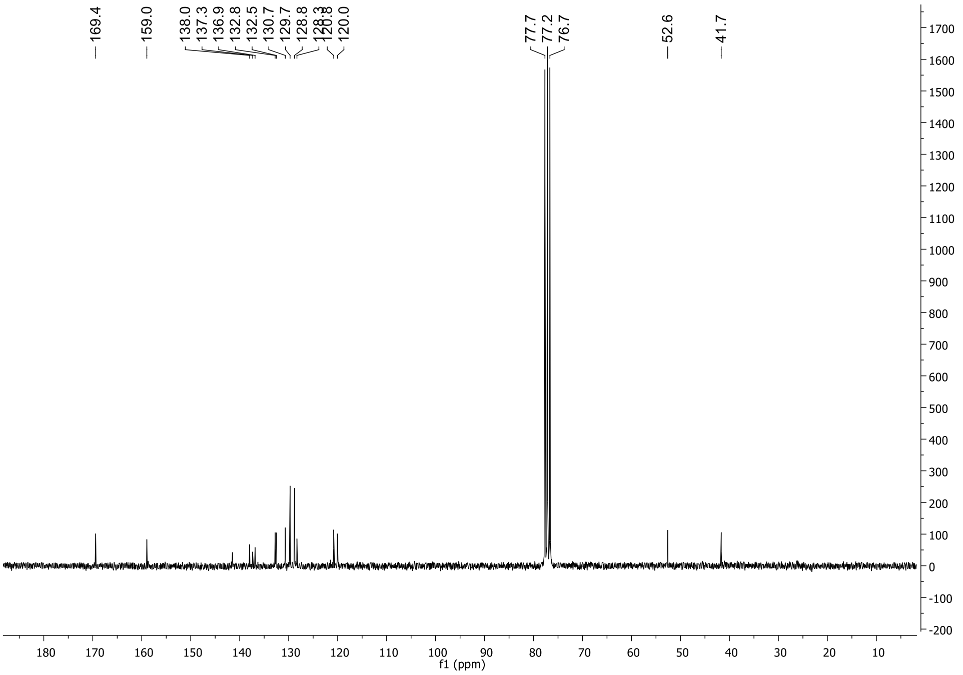
169.4
159.0
138.0
137.3
136.9
132.8
132.5
130.7
129.7
128.8
128.3
120.8
120.0
77.7
77.2
76.7
52.6
41.7
f1 (ppm)

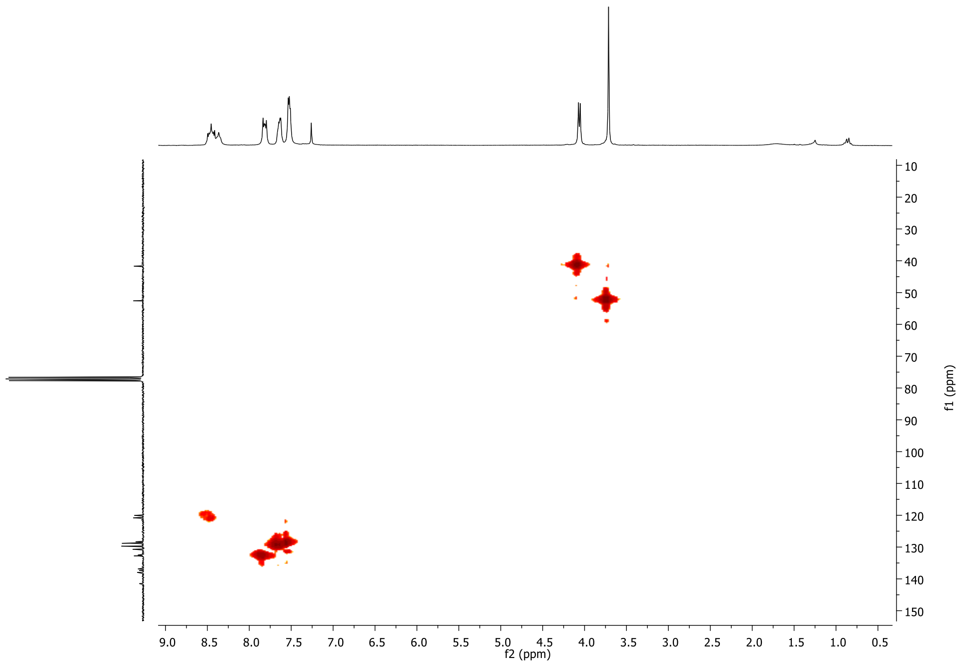
f2 (ppm)
f1 (ppm)

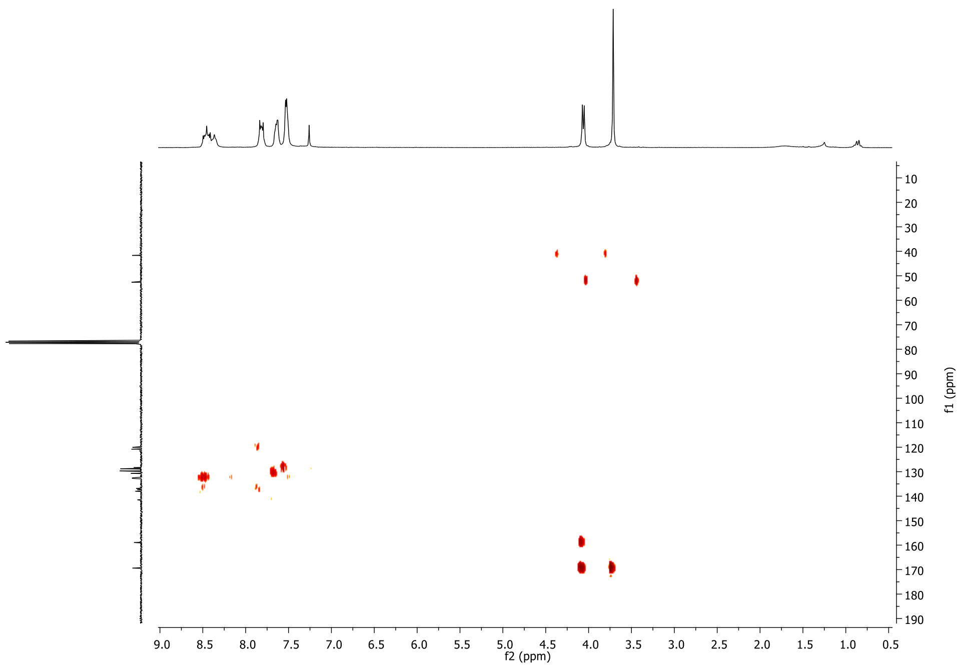
f2 (ppm)
f1 (ppm)

Compound 4b

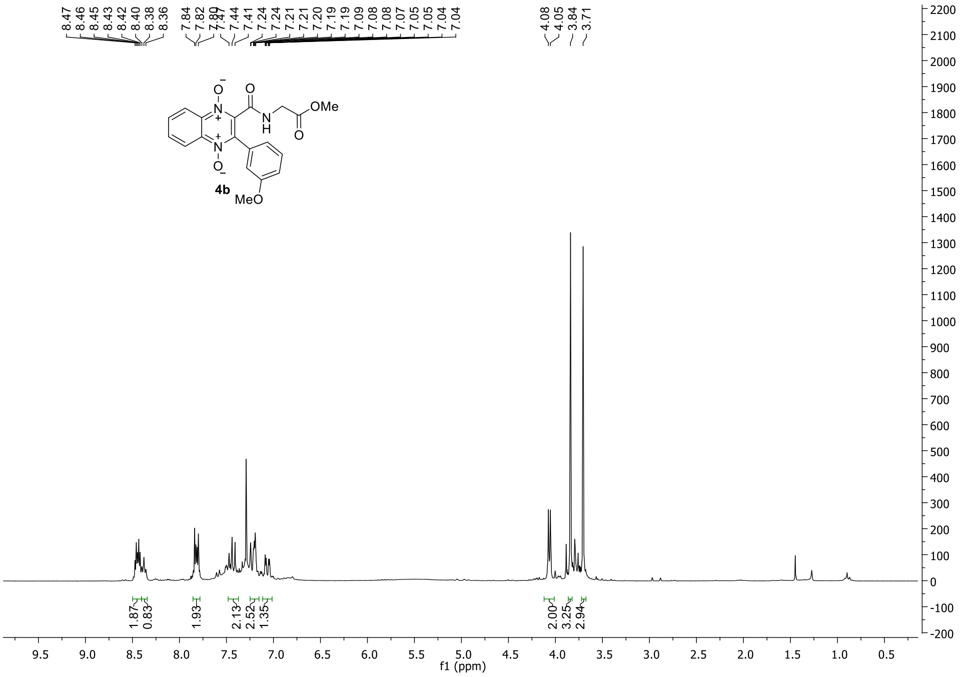
8.47
8.46
8.45
8.43
8.42
8.40
8.38
8.36
7.84
7.82
7.80
7.47
7.44
7.41
7.24
7.24
7.21
7.21
7.20
7.19
7.09
7.08
7.08
7.07
7.05
7.05
7.04
7.04
4.08
4.05
3.84
3.71
OMe
H
MeO
4b
1.87
0.83
1.93
2.13
2.52
1.35
2.00
3.25
2.94
f1 (ppm)

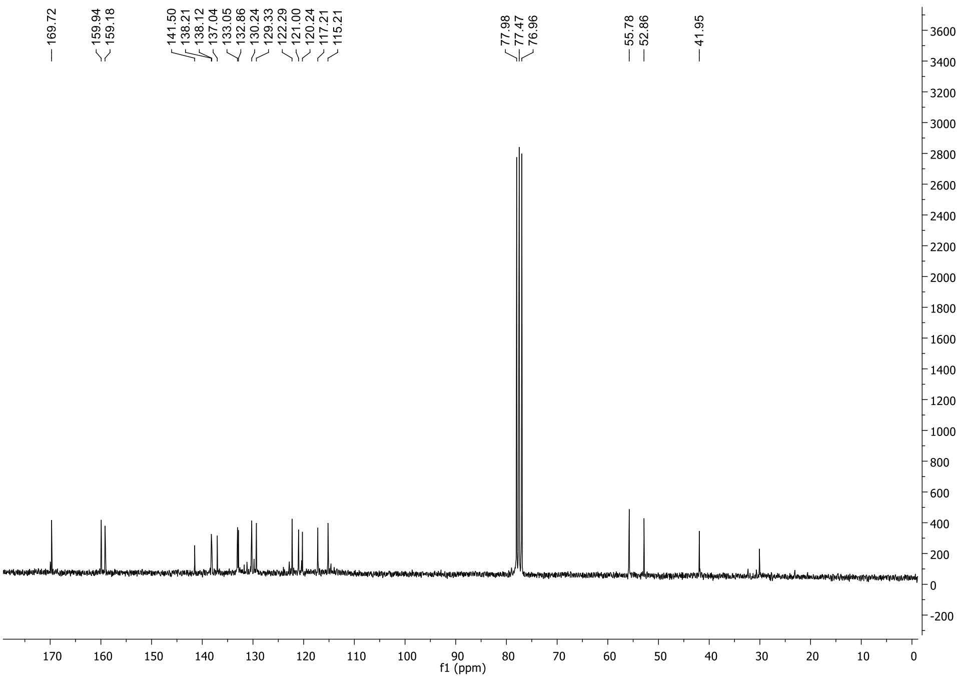
169.72
159.94
159.18
141.50
138.21
138.12
137.04
133.05
132.86
130.24
129.33
122.29
121.00
120.24
117.21
115.21
77.98
77.47
76.96
55.78
52.86
41.95
f1 (ppm)

Compound 4c

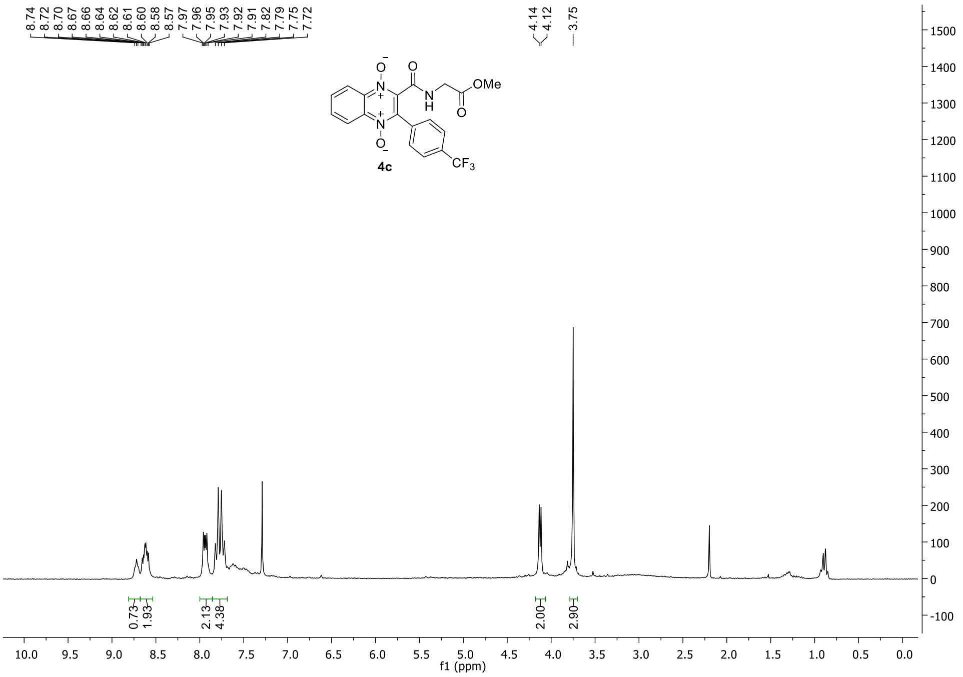
8.74 8.72 8.70 8.67 8.66 8.64 8.62 8.61 8.60 8.58 8.57 7.97 7.96 7.95 7.93 7.92 7.91 7.82 7.79 7.75 7.72
4.14 4.12
3.75
OMe
4c
CF3
0.73 1.93 2.13 4.38 2.00 2.90
f1 (ppm)

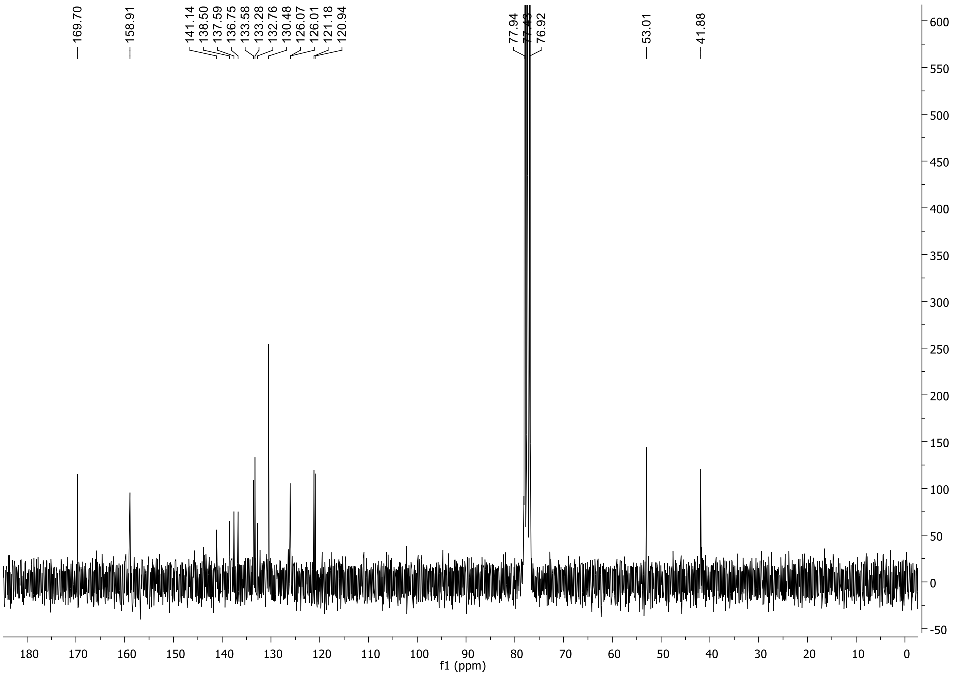
169.70
158.91
141.14 138.50 137.59 136.75 133.58 133.28 132.76 130.48 126.07 126.01 121.18 120.94
77.94 77.43 76.92
53.01
41.88
f1 (ppm)

Compound 4d

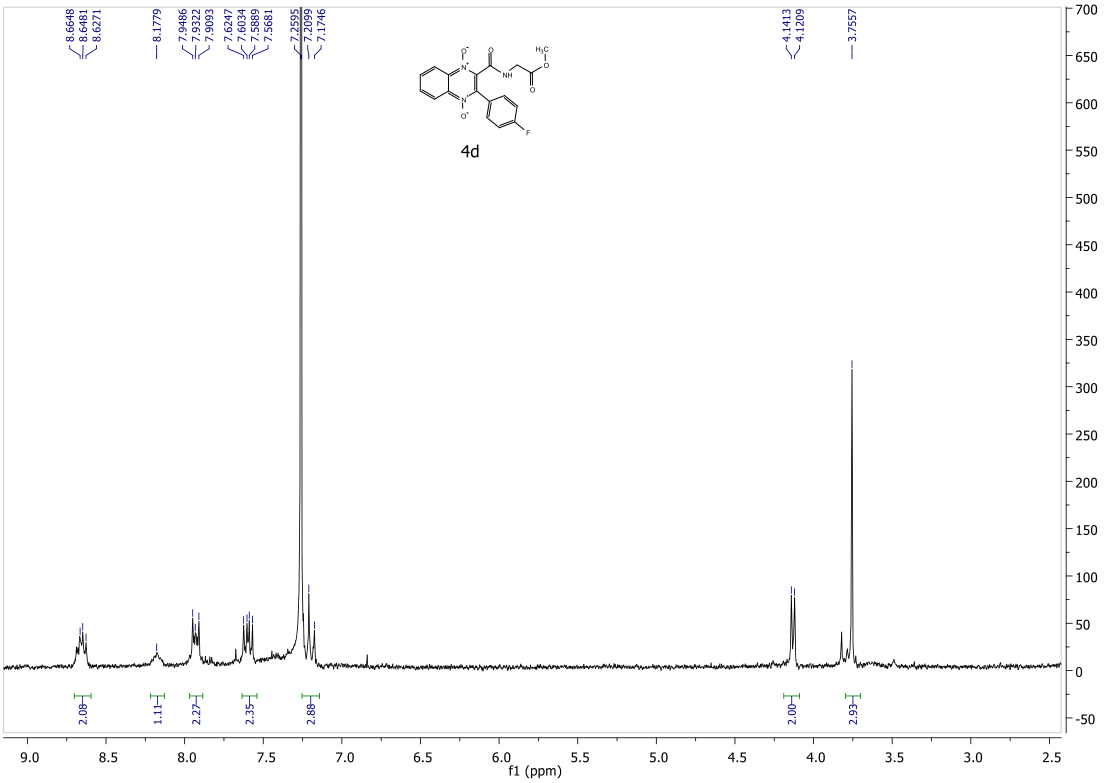

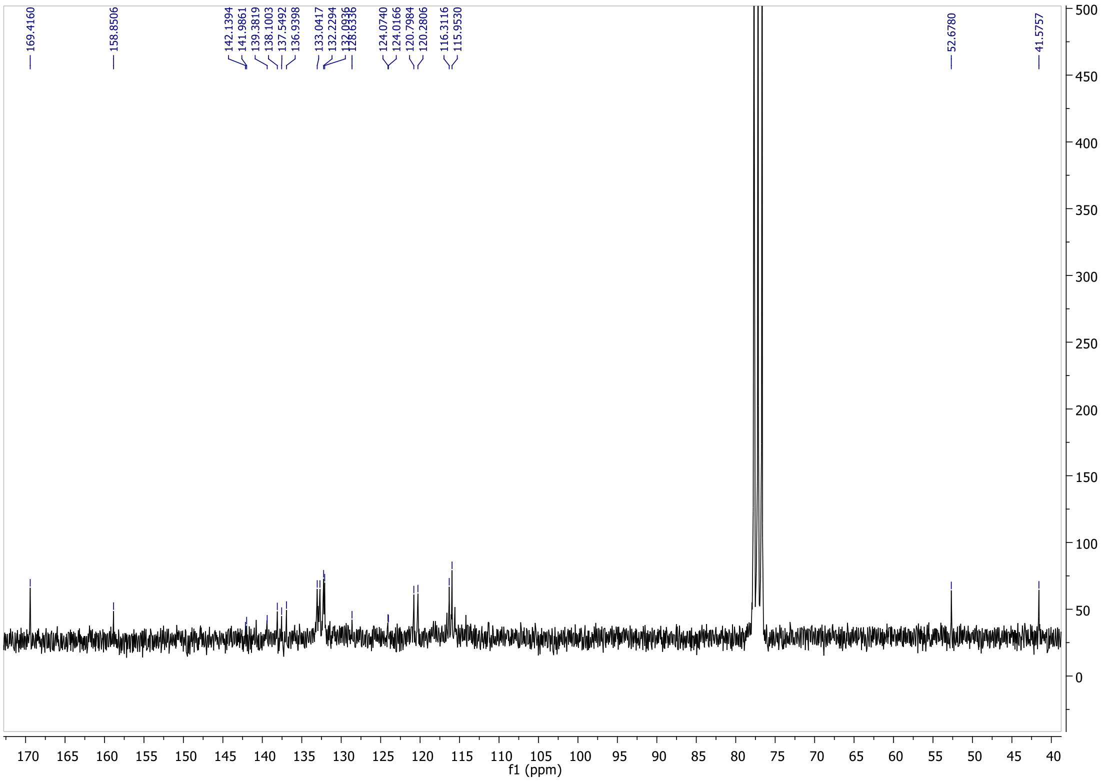

Compound 4e

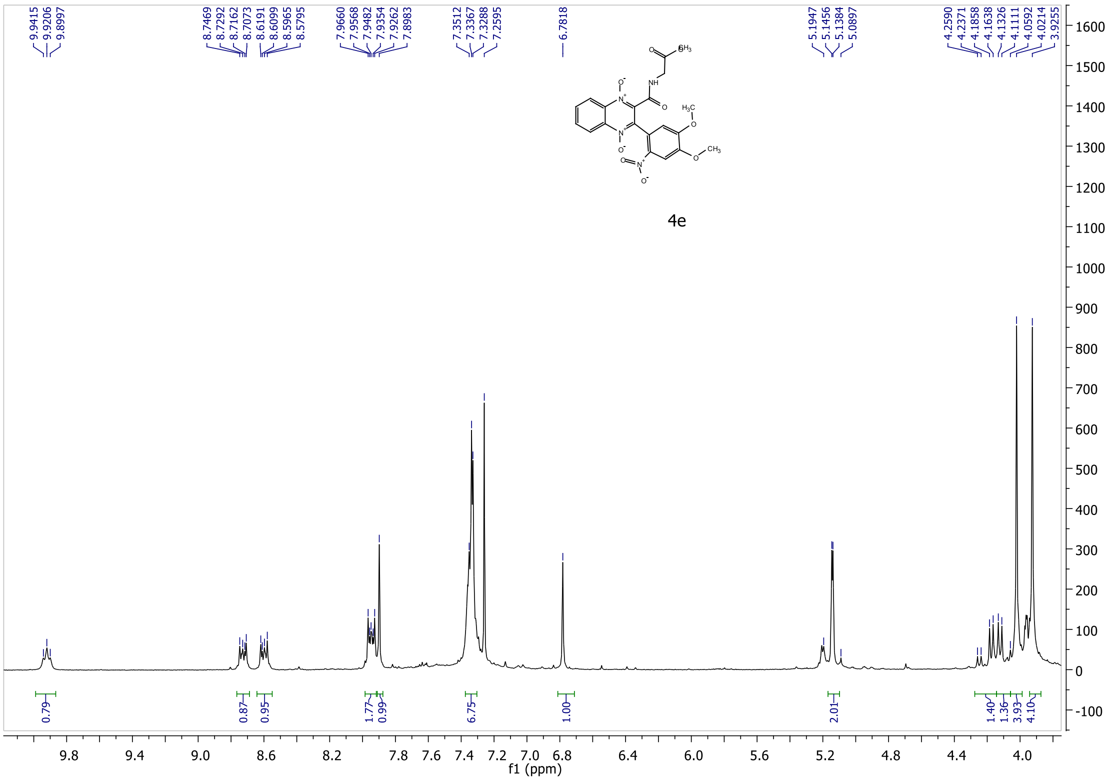

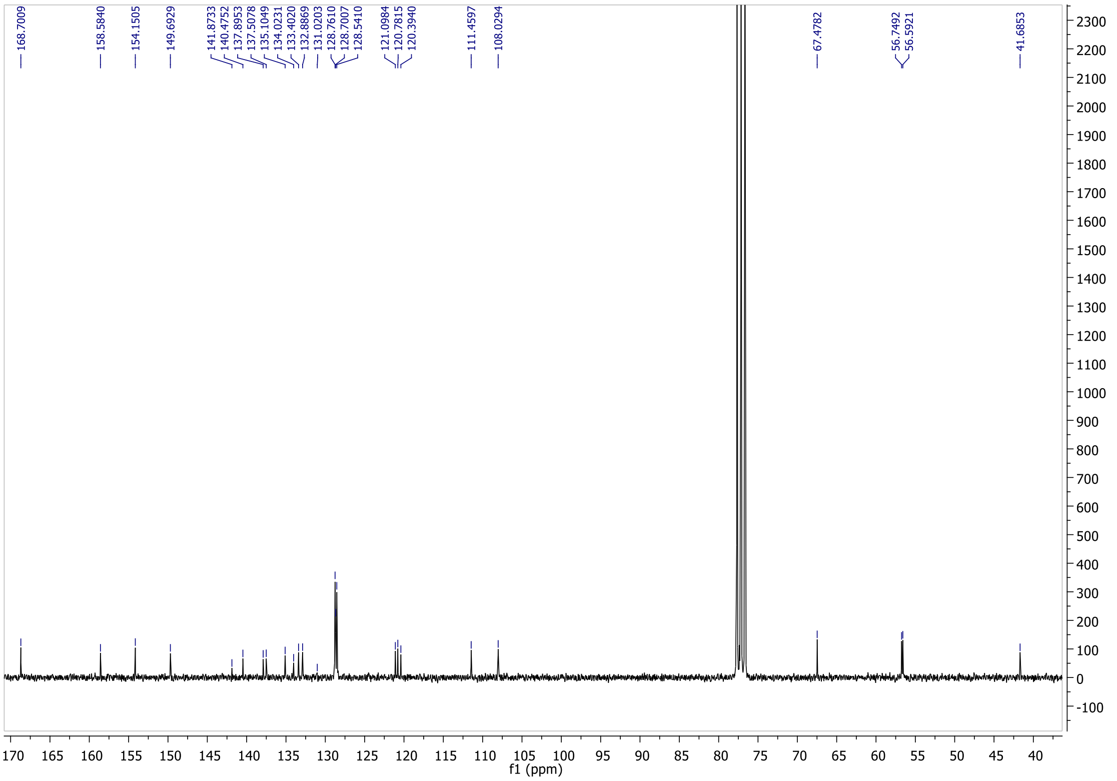

Compound 4f

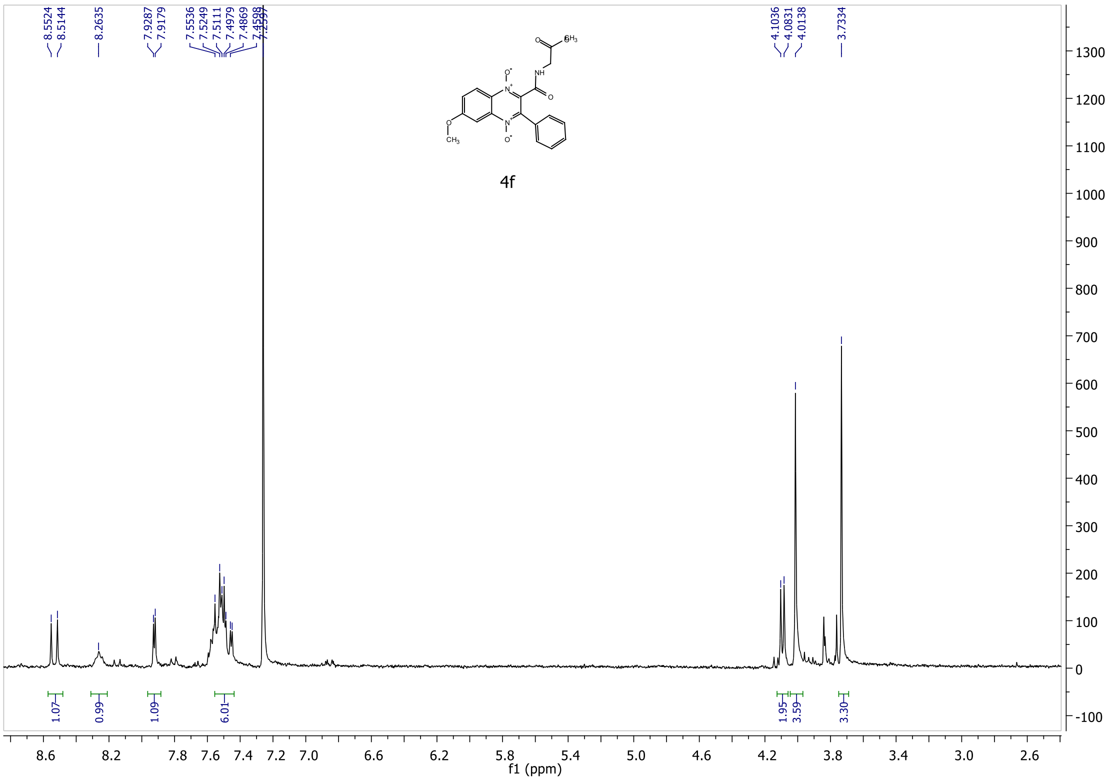
8.5524
8.5144
8.2635
7.9287
7.9179
7.5536
7.5249
7.5111
7.4979
7.4869
7.4598
7.2597
4.1036
4.0831
4.0138
3.7334
4f
1.07
0.99
1.09
6.01
1.95
3.59
3.30
f1 (ppm)

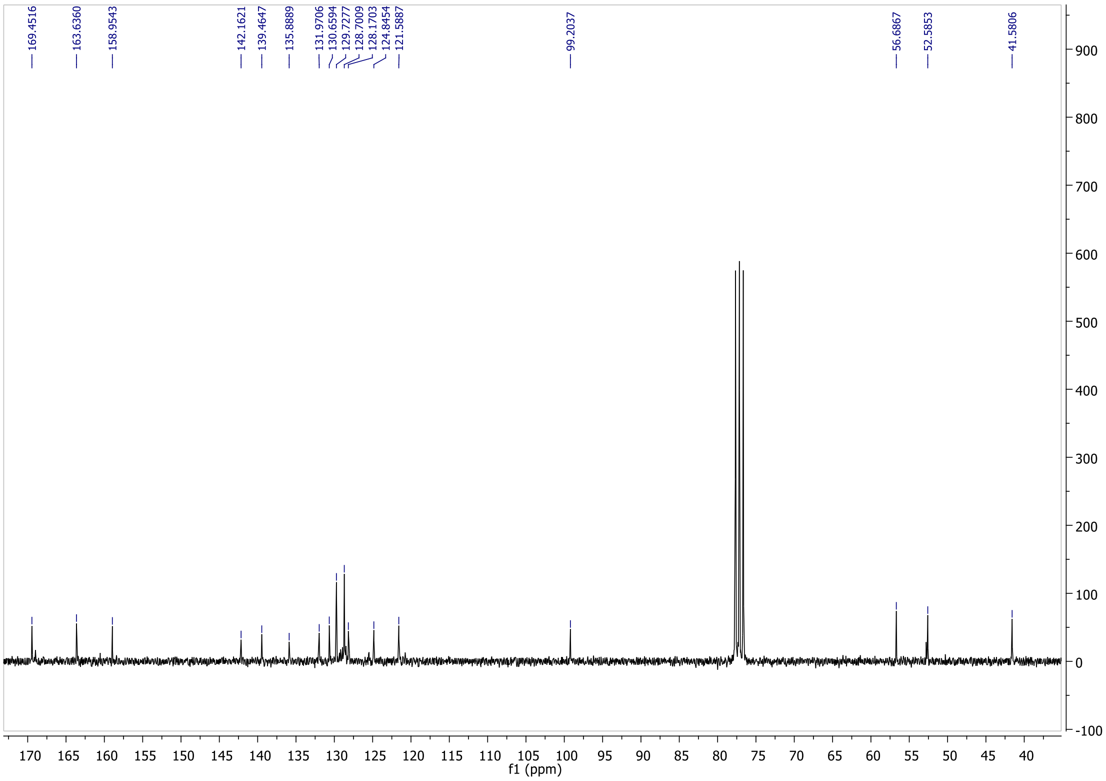
169.4516
163.6360
158.9543
142.1621
139.4647
135.8889
131.9706
130.6594
129.7277
128.7009
128.1703
124.8454
121.5887
99.2037
56.6867
52.5853
41.5806
f1 (ppm)

Compound 4g

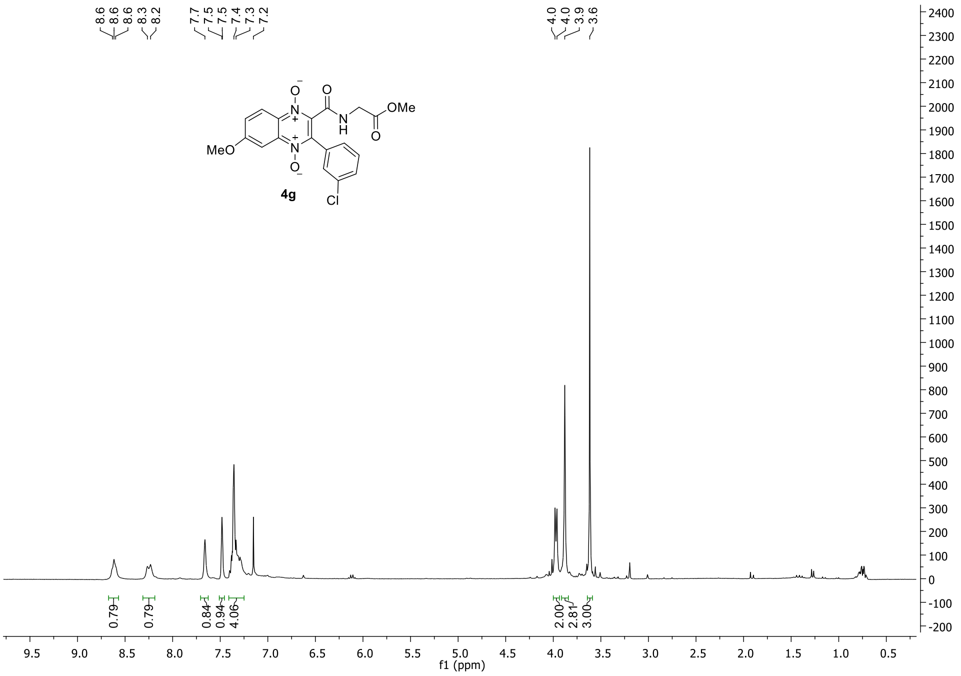
8.6
8.6
8.6
8.3
8.2
7.7
7.5
7.5
7.4
7.3
7.2
4.0
4.0
3.9
3.6
O
N
O
OMe
N
H
O
MeO
N
O
4g
Cl
0.79
0.79
0.84
0.94
4.06
2.00
2.81
3.00
f1 (ppm)

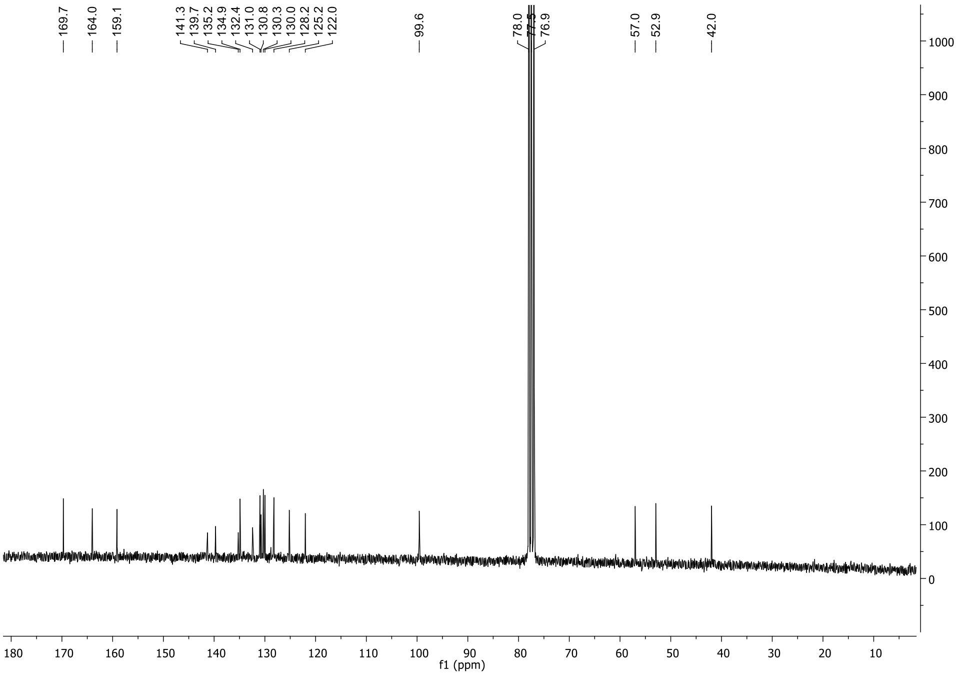
169.7
164.0
159.1
141.3
139.7
135.2
134.9
132.4
131.0
130.8
130.3
130.0
128.2
125.2
122.0
99.6
78.0
77.5
76.9
57.0
52.9
42.0
f1 (ppm)

Compound 4h

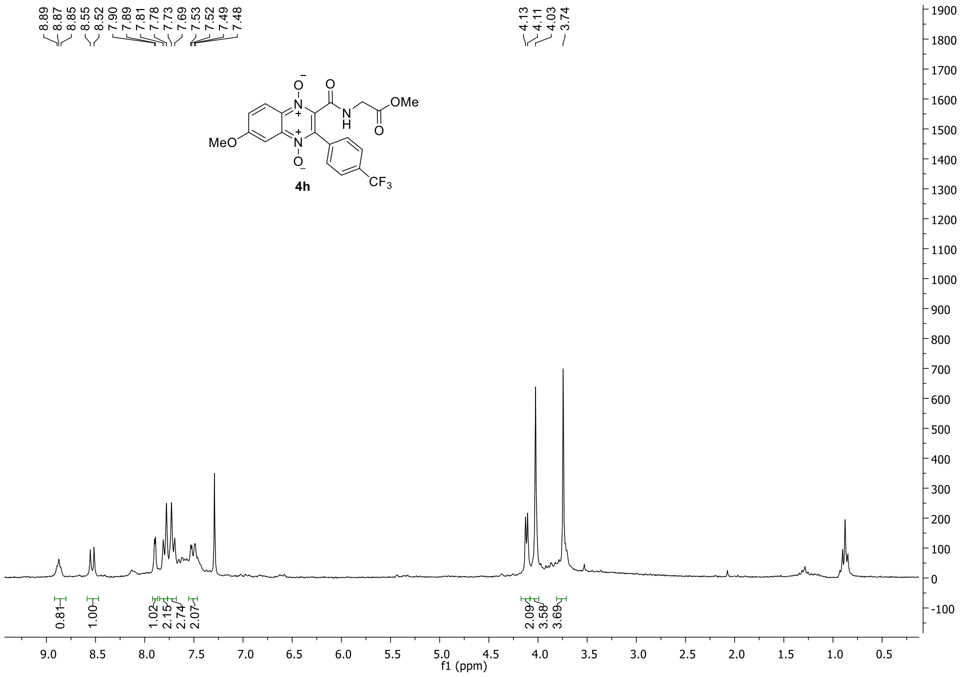

8.89 8.87 8.85 8.55 8.52 7.90 7.89 7.81 7.78 7.73 7.69 7.53 7.52 7.49 7.48
4.13 4.11 4.03 3.74
MeO
OMe
N
H
CF3
4h
0.81 1.00 1.02 2.15 2.74 2.07 2.09 3.58 3.69
f1 (ppm)

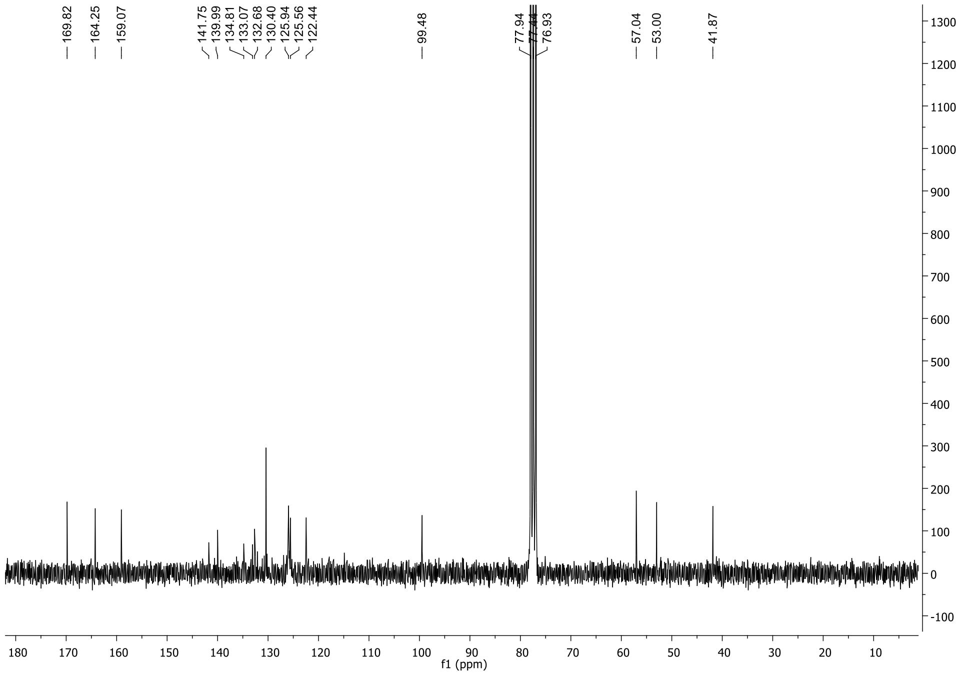

169.82 164.25 159.07 141.75 139.99 134.81 133.07 132.68 130.40 125.94 125.56 122.44 99.48 77.94 77.44 76.93 57.04 53.00 41.87
f1 (ppm)

Compound 4i

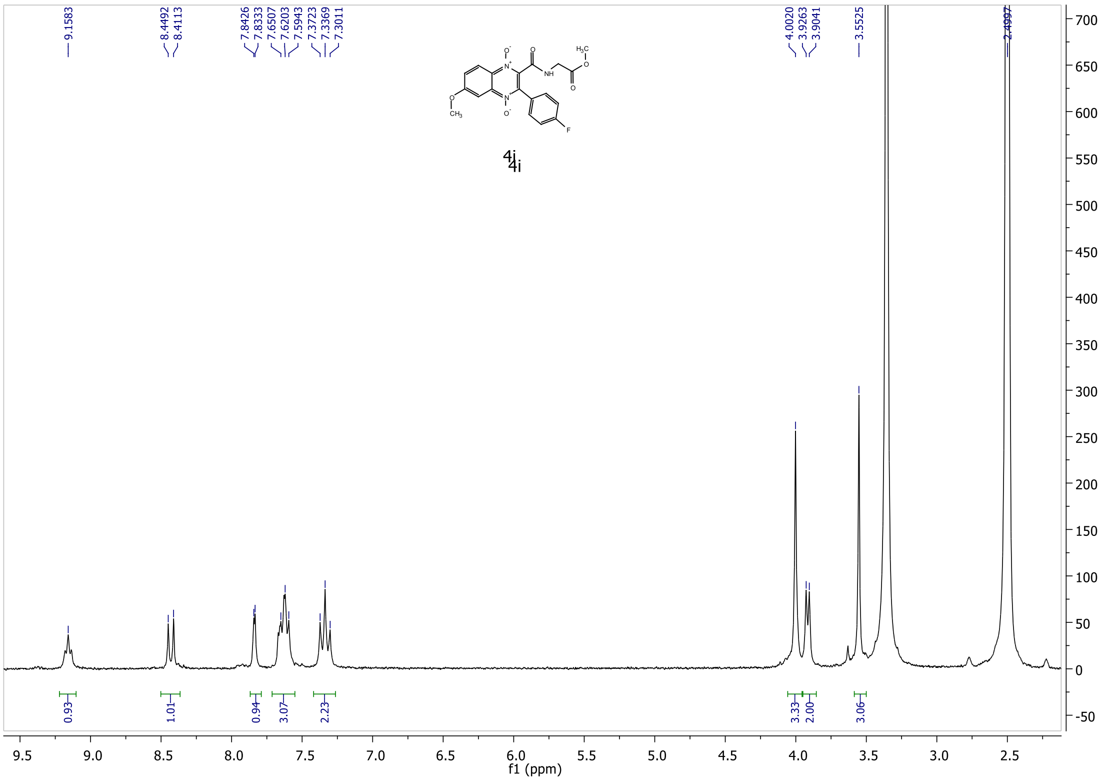

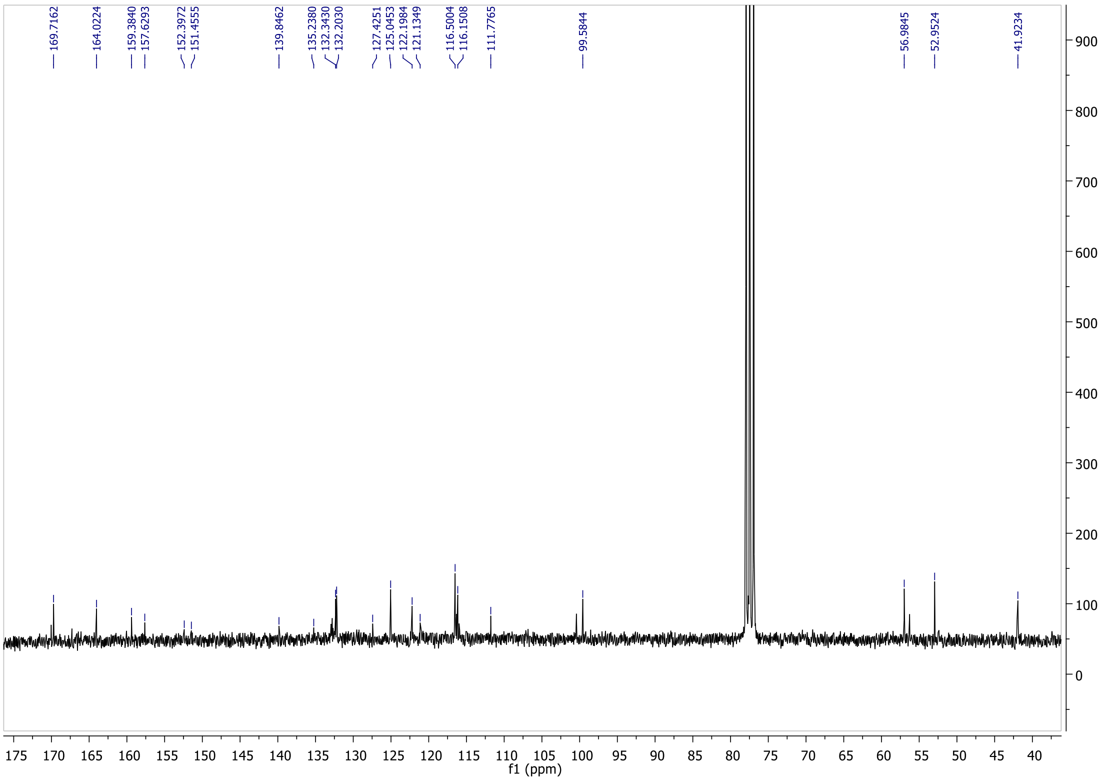

Compound 4j

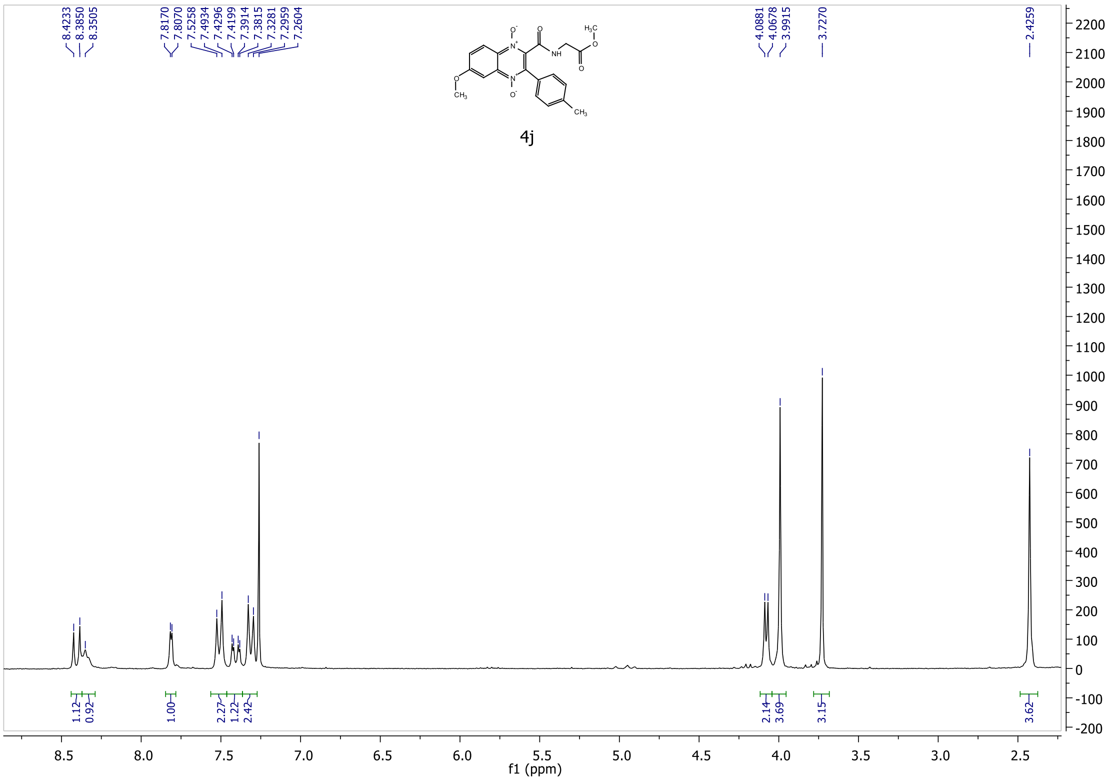
8.4233
8.3850
8.3505
7.8170
7.8070
7.5258
7.4934
7.4296
7.4199
7.3914
7.3815
7.3281
7.2959
7.2604
4.0881
4.0678
3.9915
3.7270
2.4259
4j
1.12
0.92
1.00
2.27
1.22
2.42
2.14
3.69
3.15
3.62
f1 (ppm)

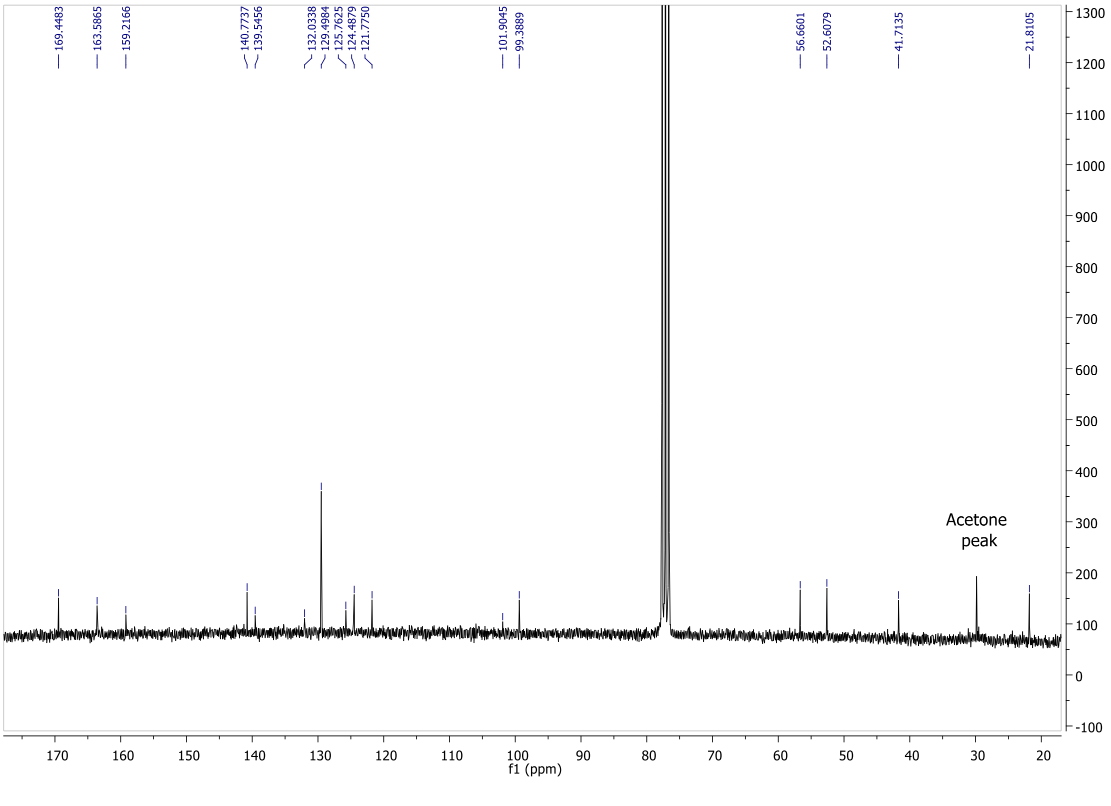
169.4483
163.5865
159.2166
140.7737
139.5456
132.0338
129.4984
125.7625
124.4879
121.7750
101.9045
99.3889
56.6601
52.6079
41.7135
21.8105
Acetone
peak
f1 (ppm)

Compound 4k

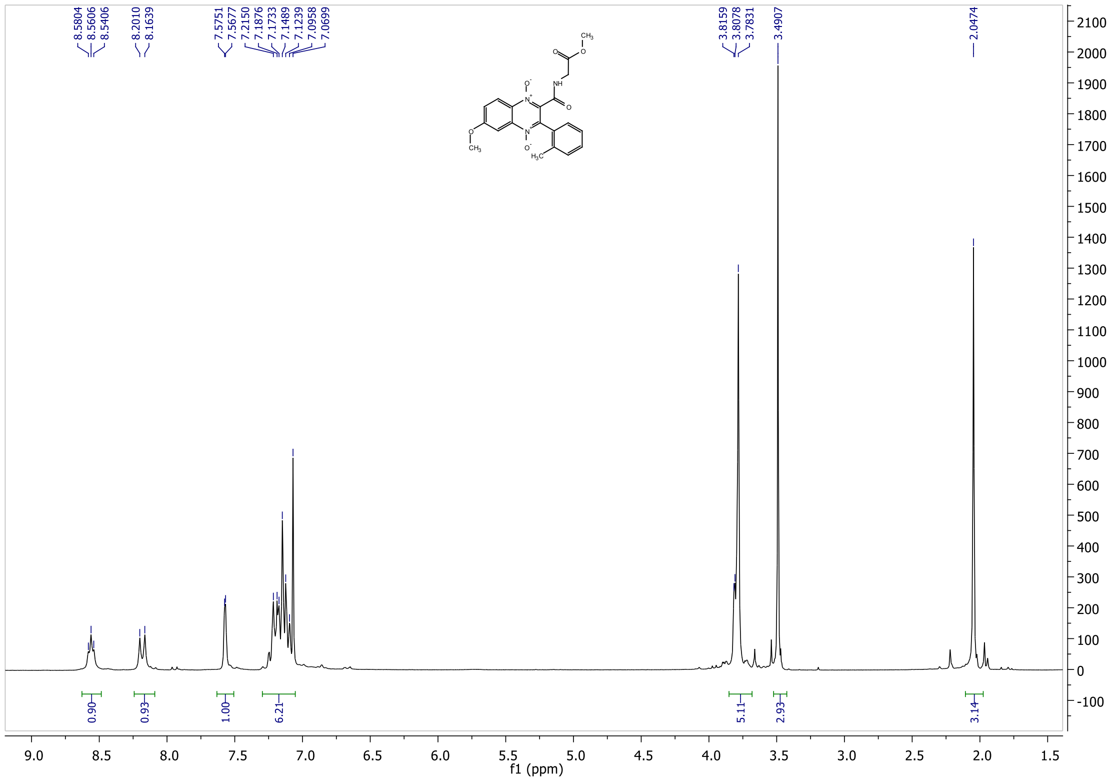

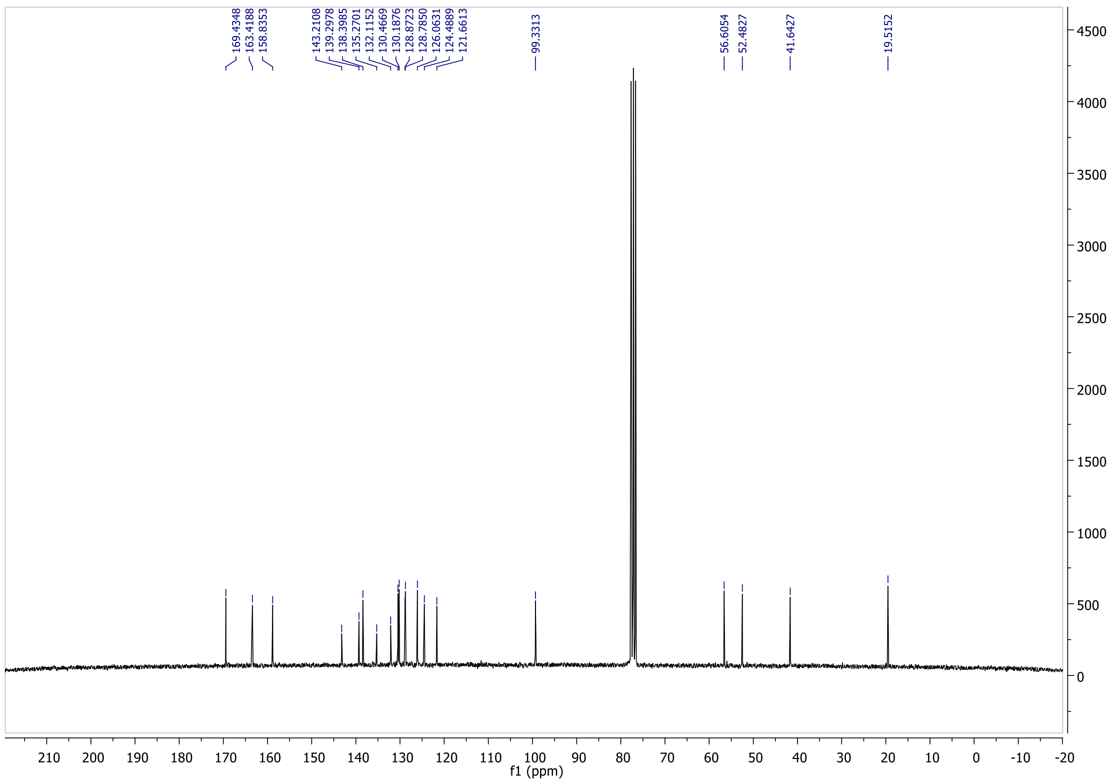

Compound 4l

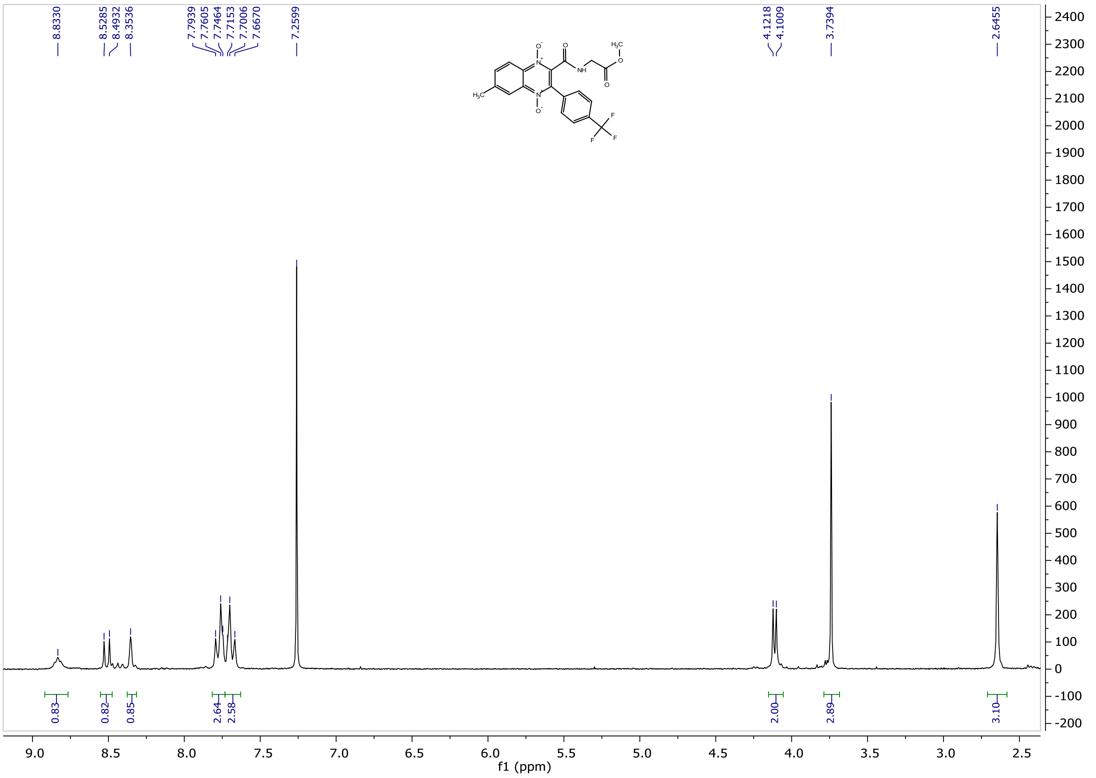

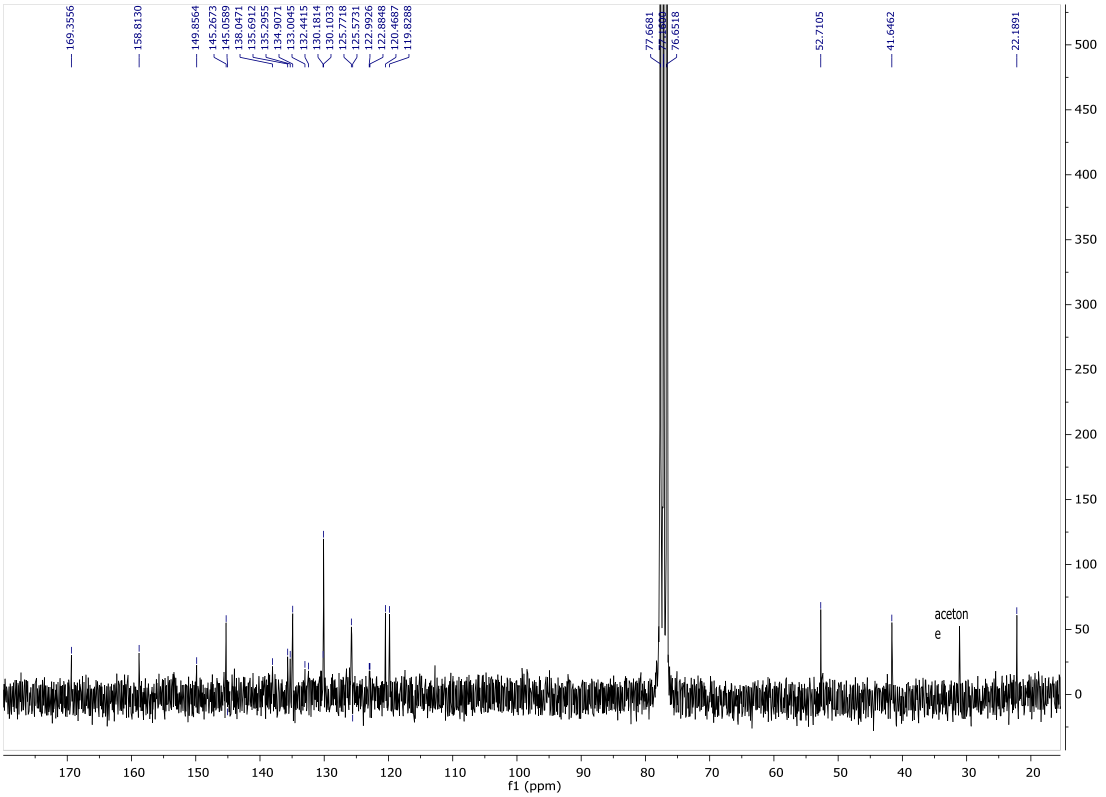

Compound 4m

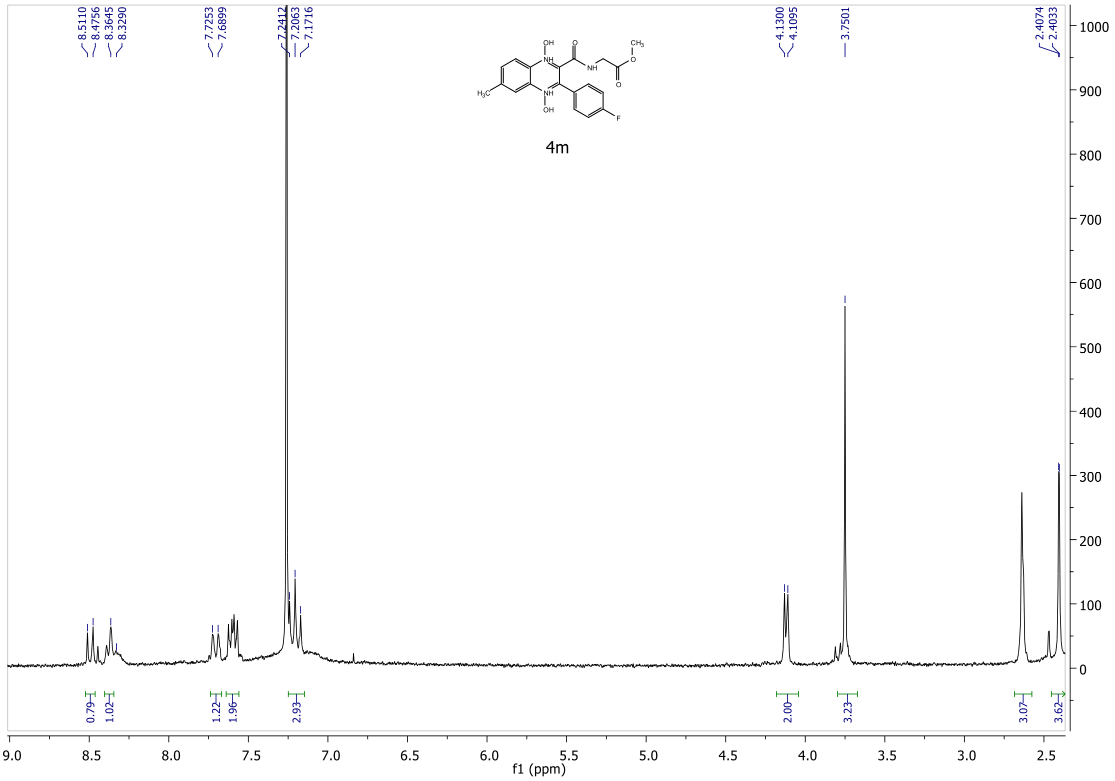

8.5110
8.4756
8.3645
8.3290
7.7253
7.6899
7.2412
7.2063
7.1716
4.1300
4.1095
3.7501
2.4074
2.4033
4m
0.79
1.02
1.22
1.96
2.93
2.00
3.23
3.07
3.62
f1 (ppm)

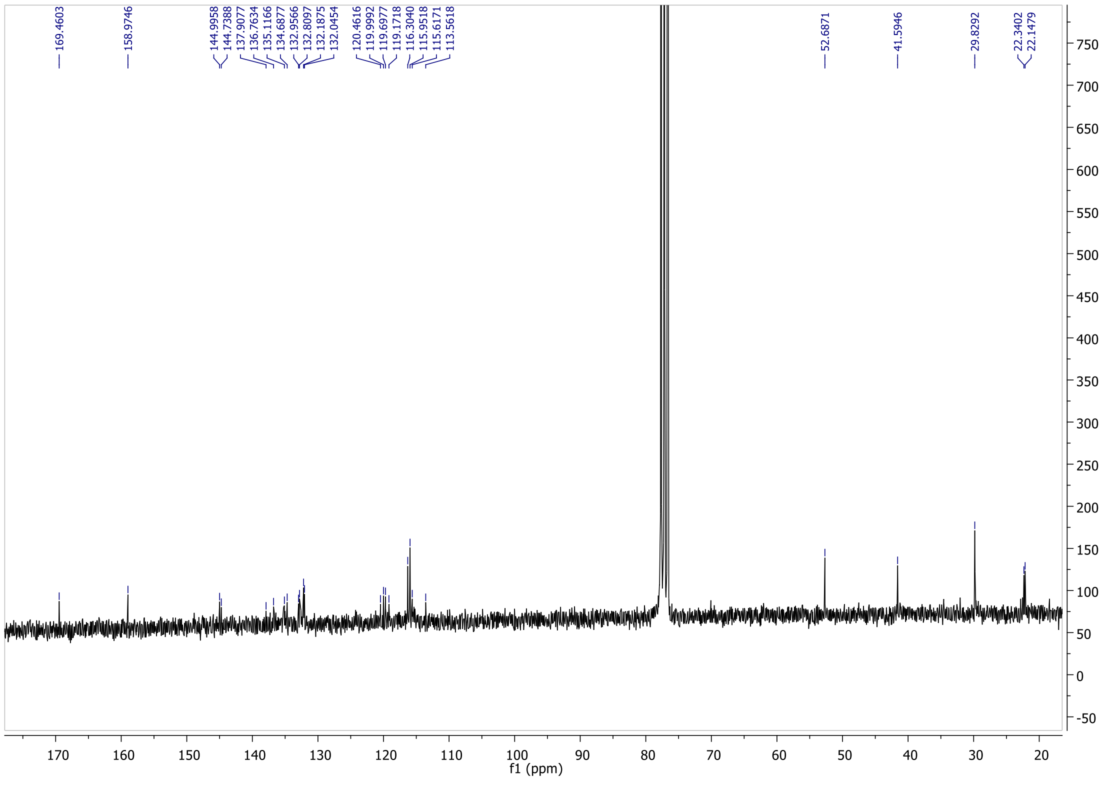

169.4603
158.9746
144.9958
144.7388
137.9077
136.7634
135.1166
134.6877
132.9566
132.8097
132.1875
132.0454
120.4616
119.9992
119.6977
119.1718
116.3040
115.9518
115.6171
113.5618
52.6871
41.5946
29.8292
22.3402
22.1479
f1 (ppm)

Compound 4n

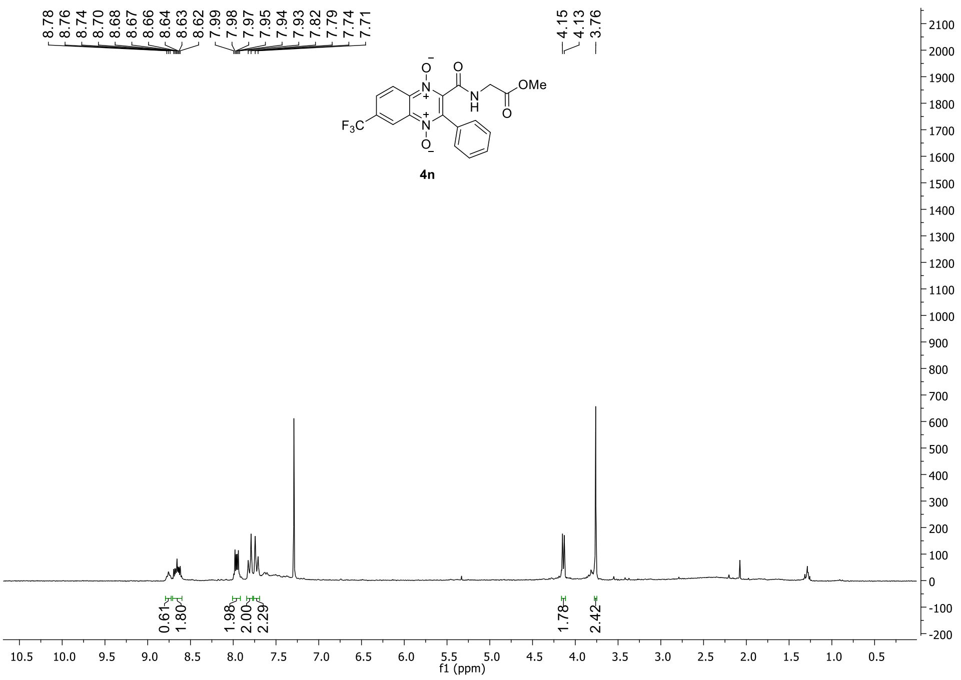

8.78
8.76
8.74
8.70
8.68
8.67
8.66
8.64
8.63
8.62
7.99
7.98
7.97
7.95
7.94
7.93
7.82
7.79
7.74
7.71
4.15
4.13
3.76
4n
0.61
1.80
1.98
2.00
2.29
1.78
2.42
f1 (ppm)

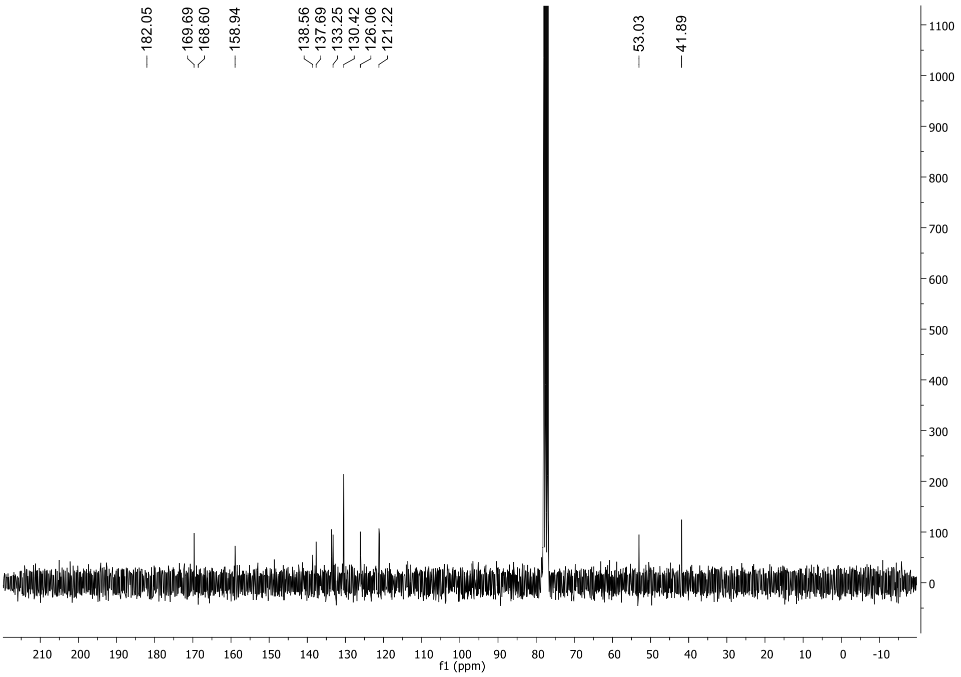

182.05
169.69
168.60
158.94
138.56
137.69
133.25
130.42
126.06
121.22
53.03
41.89
f1 (ppm)

Compound 5d

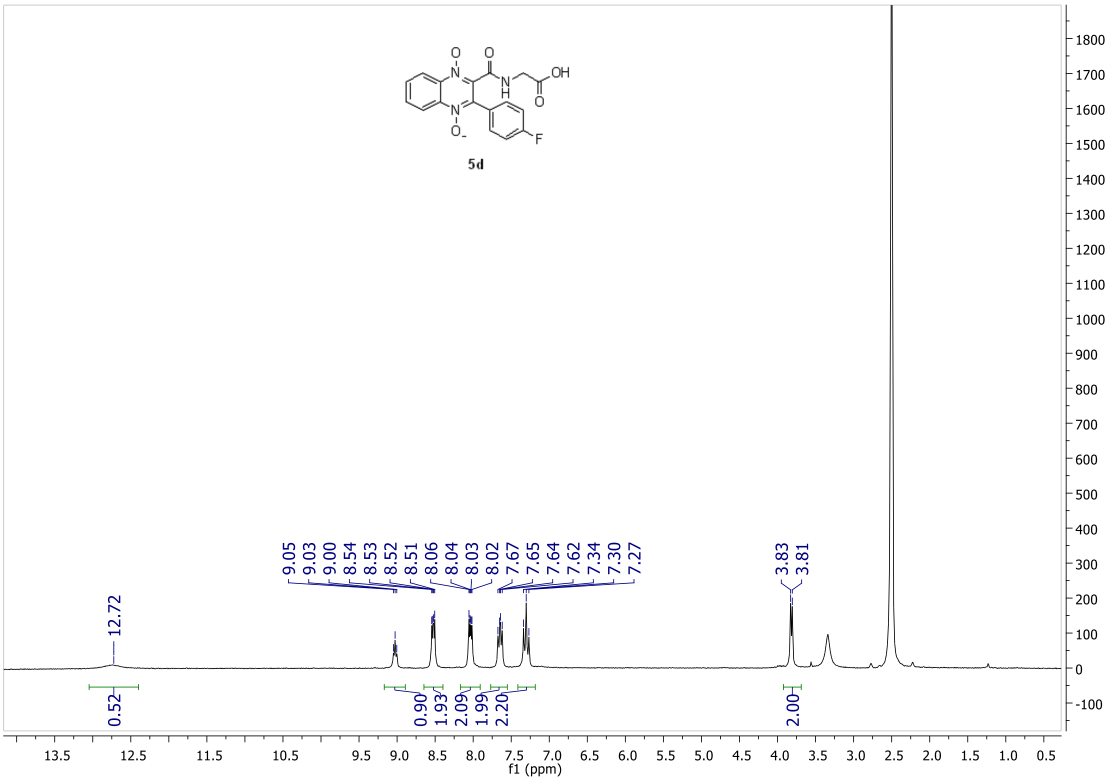

5d
12.72
9.05
9.03
9.00
8.54
8.53
8.52
8.51
8.06
8.04
8.03
8.02
7.67
7.65
7.64
7.62
7.34
7.30
7.27
3.83
3.81
0.52
0.90
1.93
2.09
1.99
2.20
2.00
f1 (ppm)

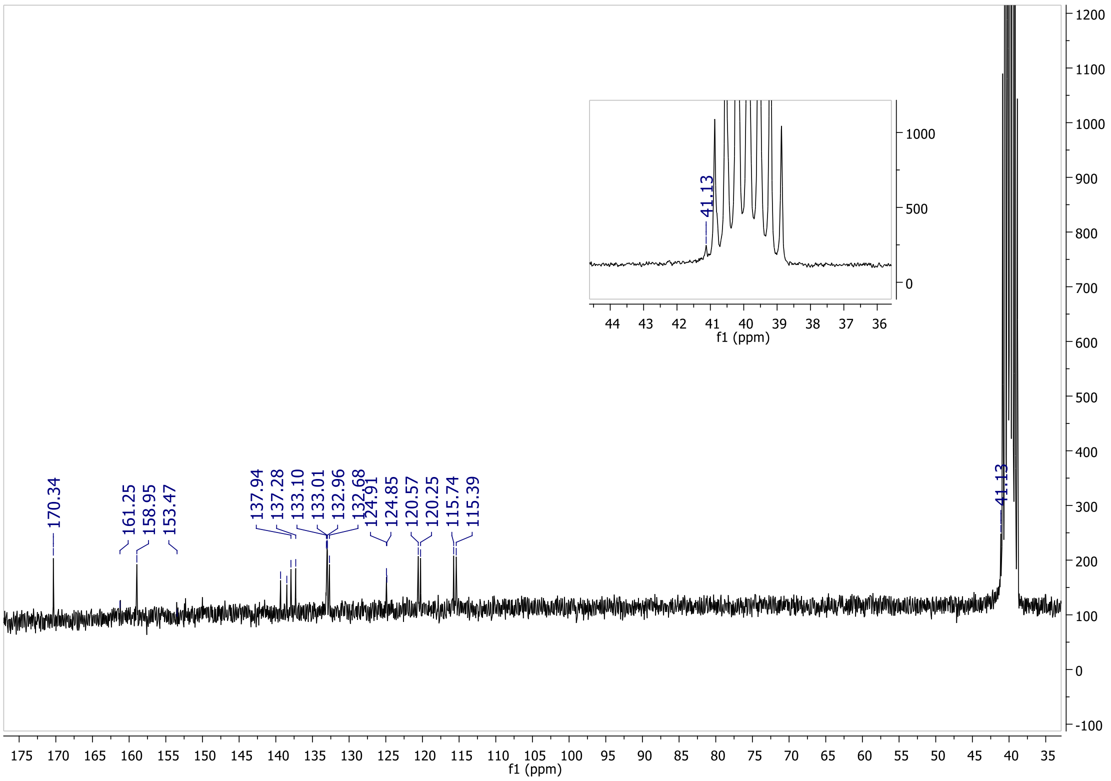

170.34
161.25
158.95
153.47
137.94
137.28
133.10
133.01
132.96
132.68
124.91
124.85
120.57
120.25
115.74
115.39
41.13
f1 (ppm)

Compound 5f

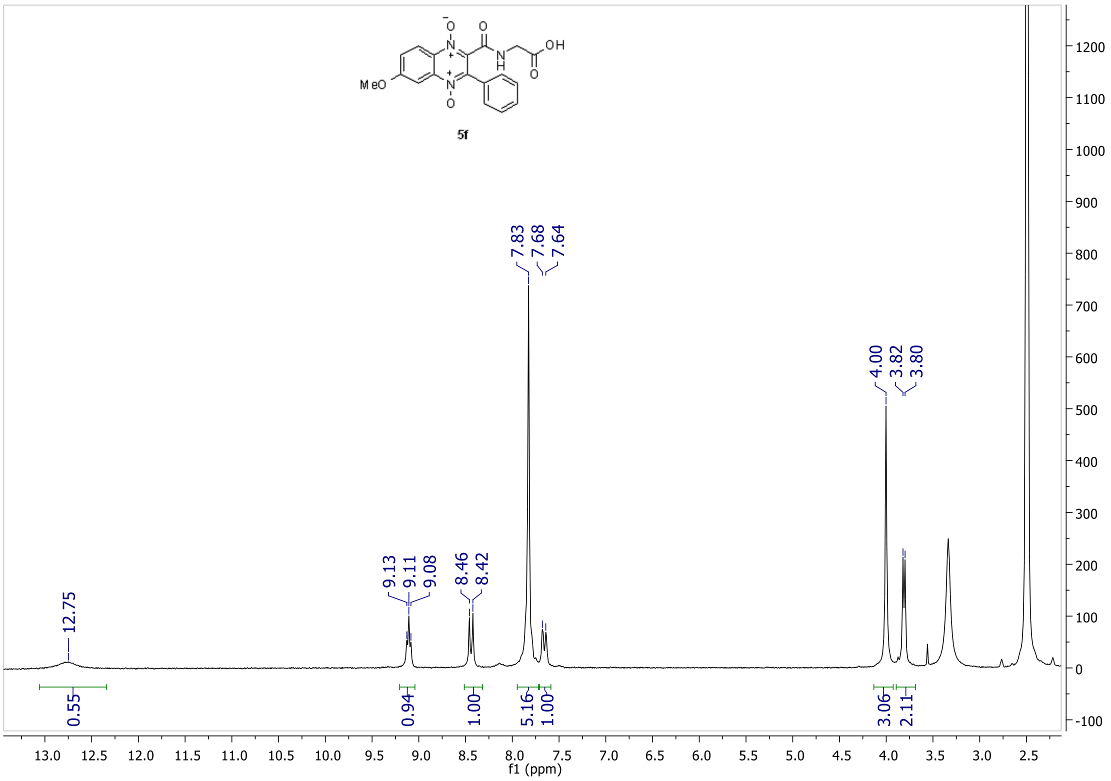

MeO
5f
12.75
9.13
9.11
9.08
8.46
8.42
7.83
7.68
7.64
4.00
3.82
3.80
0.55
0.94
1.00
5.16
1.00
3.06
2.11
f1 (ppm)

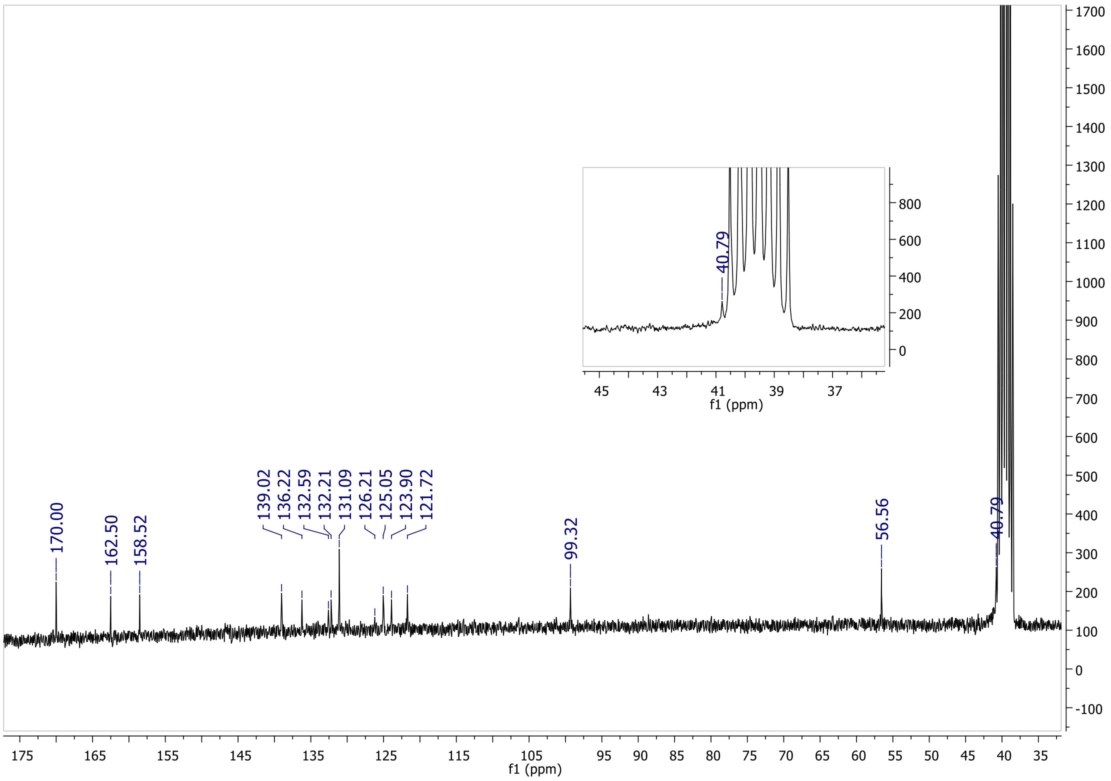

170.00
162.50
158.52
139.02
136.22
132.59
132.21
131.09
126.21
125.05
123.90
121.72
99.32
56.56
40.79
f1 (ppm)

Compound 5h

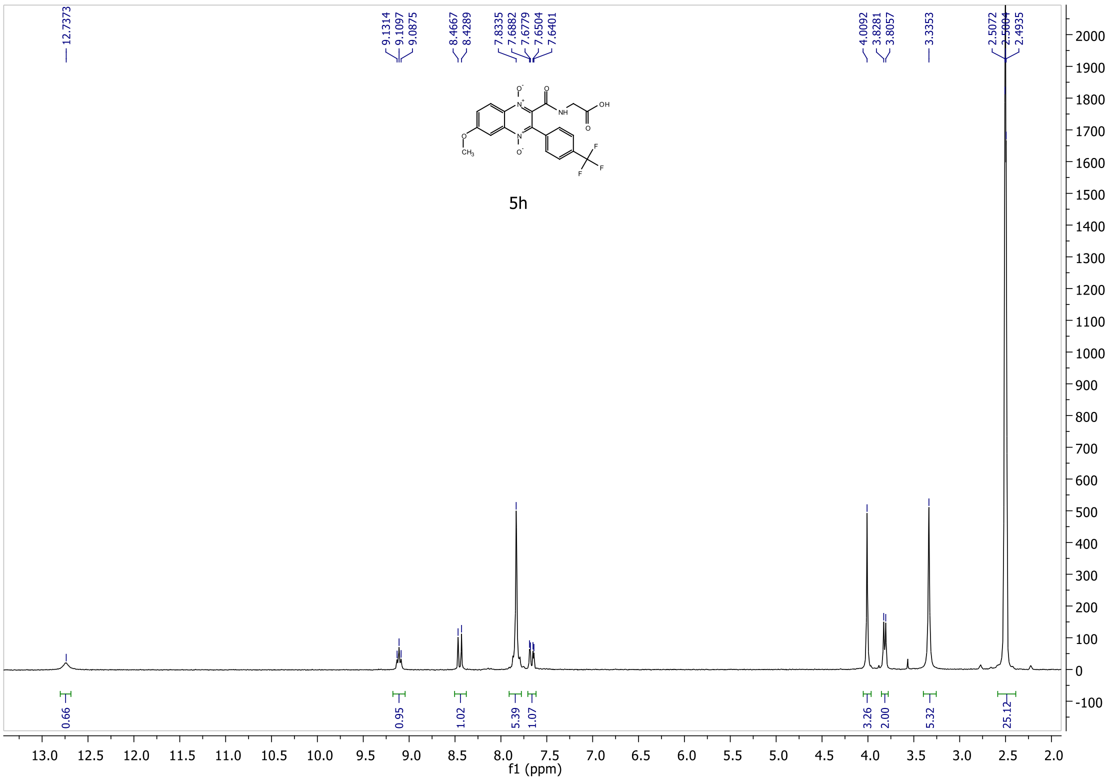

12.7373
9.1314
9.1097
9.0875
8.4667
8.4289
7.8335
7.6882
7.6779
7.6504
7.6401
4.0092
3.8281
3.8057
3.3353
2.5072
2.5004
2.4935
5h
0.66
0.95
1.02
5.39
1.07
3.26
2.00
5.32
25.12
f1 (ppm)

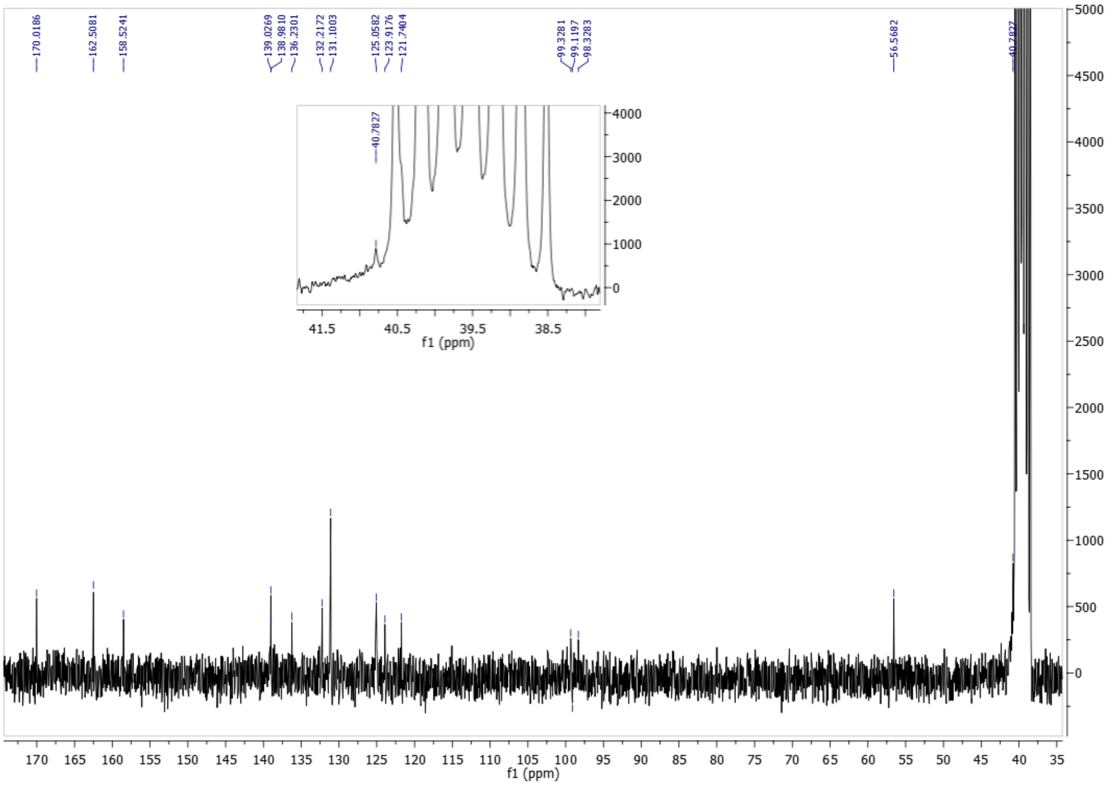

170.0186
162.5081
158.5241
139.0269
138.9810
136.2301
132.2172
131.1003
125.0582
123.9176
121.7404
99.3281
99.1197
98.3283
56.5682
40.7827
f1 (ppm)

Compound 5m

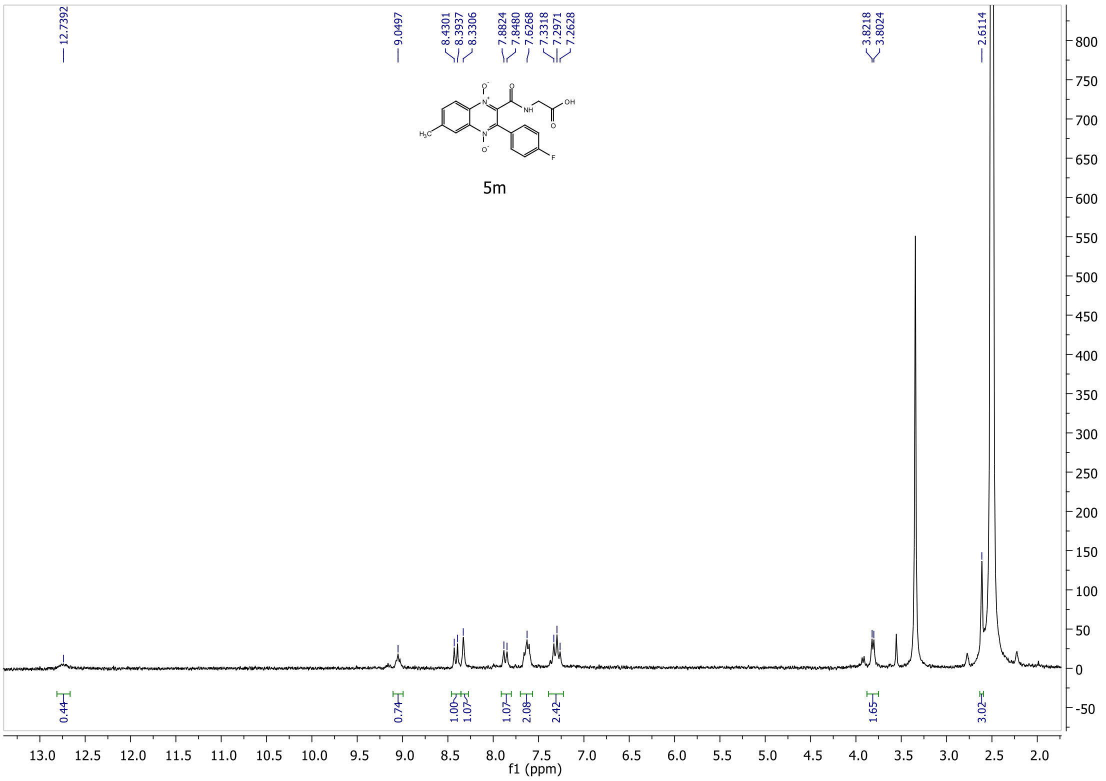

12.7392
9.0497
8.4301
8.3937
8.3306
7.8824
7.8480
7.6268
7.3318
7.2971
7.2628
3.8218
3.8024
2.6114
5m
0.44
0.74
1.00
1.07
1.07
2.08
2.42
1.65
3.02
f1 (ppm)

Compound 6

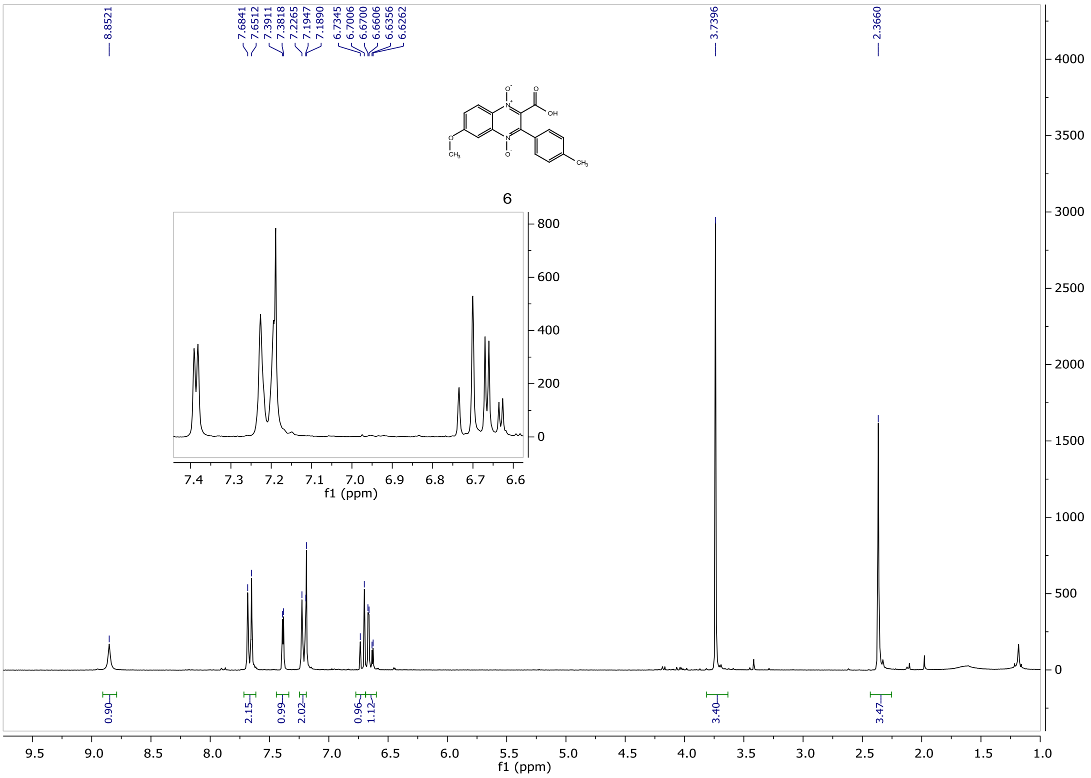

8.8521
7.6841
7.6512
7.3911
7.3818
7.2265
7.1947
7.1890
6.7345
6.7006
6.6700
6.6606
6.6356
6.6262
3.7396
2.3660
6
0.90
2.15
0.99
2.02
0.96
1.12
3.40
3.47
f1 (ppm)

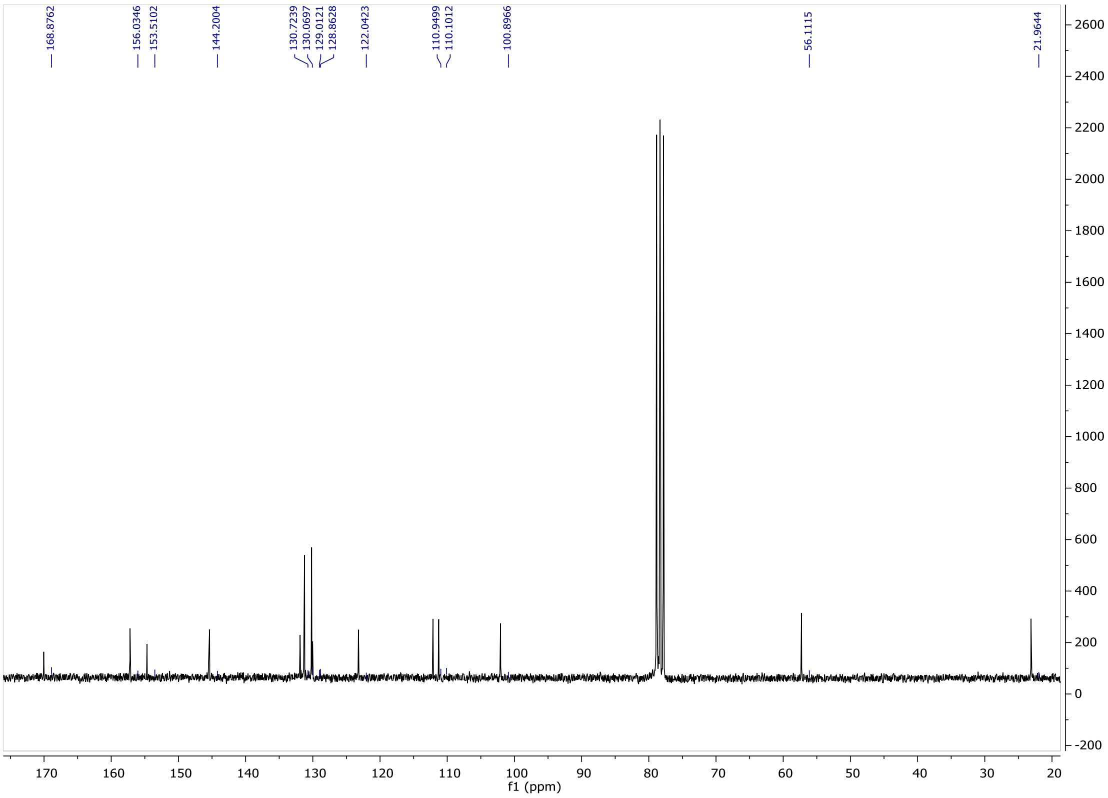

168.8762
156.0346
153.5102
144.2004
130.7239
130.0697
129.0121
128.8628
122.0423
110.9499
110.1012
100.8966
56.1115
21.9644
f1 (ppm)
